# Triazoloquinoxalines-based DNA intercalators-Topo II inhibitors: design, synthesis, docking, ADMET and anti-proliferative evaluations

**DOI:** 10.1080/14756366.2022.2080205

**Published:** 2022-05-29

**Authors:** Alaa Elwan, Helmy Sakr, Abdel-Ghany A. El-Helby, Ahmed El-morsy, Mohamed A. Abdelgawad, Mohammed M. Ghoneim, Mohamed El-Sherbiny, Khaled El-Adl

**Affiliations:** a Pharmaceutical Medicinal Chemistry & Drug Design Department, Faculty of Pharmacy (Boys), Al-Azhar University, Cairo, Egypt; b Pharmaceutical Chemistry Department, College of Pharmacy, The Islamic University, Najaf, Iraq; c Department of Pharmaceutical Chemistry, College of Pharmacy, Jouf University, Sakaka, Saudi Arabia; d Department of Pharmacy Practice, Faculty of Pharmacy, AlMaarefa University, Ad Diriyah, Saudi Arabia; e Department of Basic Medical Sciences, College of Medicine, AlMaarefa University, Riyadh, Saudi Arabia; f Department of Anatomy, Faculty of Medicine, Mansoura University, Mansoura, Egypt; g Chemistry Department, Faculty of Pharmacy, Heliopolis University for Sustainable Development, Cairo, Egypt

**Keywords:** Triazoloquinoxaline, docking, DNA intercalators, Topo II inhibitors

## Abstract

Sixteen [1, 2, 4]triazolo[4,3-a]quinoxalines as DNA intercalators-Topo II inhibitors have been prepared and their anticancer actions evaluated towards three cancer cell lines. The new compounds affected on high percentage of MCF-7. Derivatives **7e**, **7c** and **7b** exhibited the highest anticancer activities. Their activities were higher than that of doxorubicin. Molecular docking studies showed that the HBA present in the chromophore, the substituted distal phenyl moiety and the extended linkers enable our derivatives to act as DNA binders. Also, the pyrazoline moiety formed six H-bonds and improved affinities with DNA active site. Finally, **7e**, **7c** and **7b** exhibited the highest DNA affinities and act as traditional intercalators of DNA. The most active derivatives **7e**, **7c, 7b, 7g** and **6e** were subjected to evaluate their Topo II inhibition and DNA binding actions. Derivative **7e** exhibited the highest binding affinity. It intercalates DNA at IC_50_ = 29.06 µM. Moreover, compound **7e** potently intercalates DNA at an IC_50_ value of 31.24 µM. Finally, compound **7e** demonstrated the most potent Topo II inhibitor at a value of 0.890 µM. Compound **7c** exhibited an equipotent IC_50_ value (0.940 µM) to that of doxorubicin. Furthermore, derivatives **7b, 7c, 7e** and **7g** displayed a high ADMET profile.

## 1. Introduction

**Figure 1.**
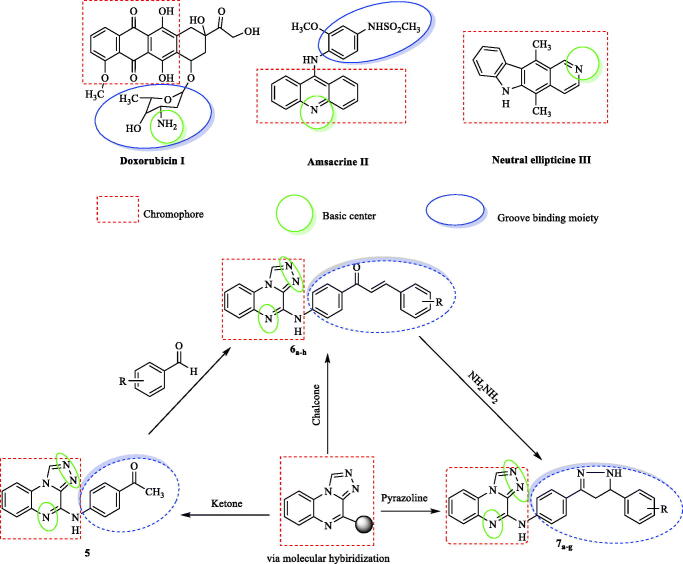
Approved DNA intercalators derivatives main pharmacophoric groups.

DNA is the main aim for hallmark genetic diseases, such as cancer, demonstrates an important role in many diversity of cellular processes^1^. Intercalators reversibly act on the DNA double helix^2^. Many anticancer DNA intercalators are clinically used^2^^,^^3^. Intercalators were transferred to the hydrophobic region between two neighbouring DNA base pairs^3^^,^^4^. There has been a lot of research concentrated on the new prepared compounds' action when bound to DNA non-covalently^5^. The target actions can lead to cellular death due to disrupting replication and/or transcription. Accordingly, anticancer agents that bind to DNA have potential applications. The binding of the intercalators with DNA may be through insertion between DNA base pairs, minor or major groove binding and/or electrostatic reactions^6^. DNA intercalators have three main structural groups, i) Chromophore (planar polyaromatic rings) that binds to DNA^3^^,^^7^. ii) Cationic species (e.g. protonated amino gp) interact with the phosphate-sugar DNA region^8^. iii) Side chain that can inhibit DNA minor groove^9–11^ (Figure 1).

Anticancer drugs binding have three principally different ways. First, the anticancer medicines react with the DNA bounded protein so control transcription factors and polymerases. The second is through interfering with transcriptional activity, where RNA binds to DNA to perform triple helical DNA or DNA-RNA hybrids. The third is by minor groove binders where derivatives non-covalently bind to DNA^12^^,^^13^.

The chromophores are placed between nearby DNA base pairs forming strong non-covalent interactions^14^^,^^15^. These interactions lead to DNA distortion and uncoiling^14^, also interfering with the detection and function of the associated proteins or enzymes leading to the failure of DNA repair systems, transcription processes, and replication of DNA^14^.

In addition, the placing of chromophores between DNA bases results in DNA lengthening and decreasing DNA helical twists. The groove binding ligands may be considered like standard key and lock models. Unlike intercalation, groove binders do not make huge DNA conformational changes. In addition, they are usually semi-circular-shaped ligands that bind to the DNA minor groove^13^.

Intercalators cab be classified into two types classical (mono-intercalators) and threading intercalators^1^. The threading intercalation occurs if there are two groove binding side chains. One side chain is directed to the major groove and the other to the minor groove^14^. DNA intercalators as anticancer are already applied or still under clinical trials (e.g. doxorubicin I^16^, amsacrine II^17^, ellipticine III^9^) (Figure 1).

**Figure 2.**
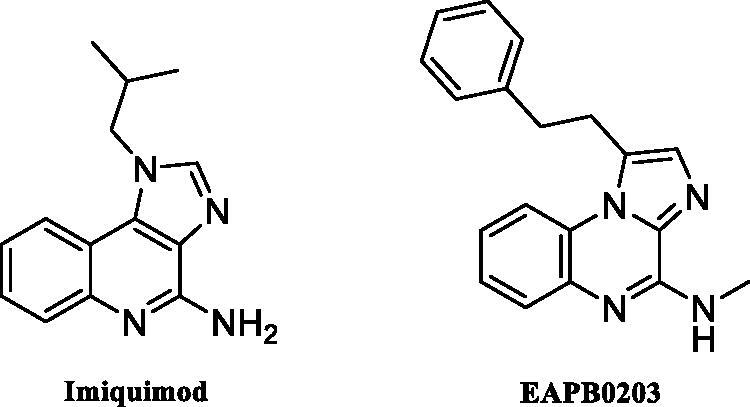
Structures of imiquimod and EAPB0203.

The imidazoquinoline, imiquimod (Figure 2) is effective in the treatment of skin and breast cancer of different types. Also, its effectiveness in other cancer types of treatment is demonstrated^18^. EAPB0203 (Figure 2) was recognised to have 45 and 110 fold more active against melanoma A375 cancer cells than imiquimod and fotemustine respectively^19^. Moreover, it was confirmed to have anticancer activities against leukaemia in different types^20^. Anastrozole as triazole containing drug was established to have anticancer activity against breast cancer^20^.

Quinoxaline structure is the scaffold of numerous DNA intercalators^21–26^. The novel anticancer discovering or developing DNA intercalators is one of the extremely significant objectives in medicinal chemistry^27^. Quinoxaline derivatives were reported to have high anticancer activities through intercalation of DNA^28^ e.g. echinomycin. It exhibited high activities against a variety of cancers in phases I and II clinical trials^29^.

Depending on the mentioned facts, and the extension of producing new anti-cancers^30–36^, especially that intercalators for DNA^21–24^^,^^37–40^, it was reported herein modifications of EAPB0203 through hybridisation with privileged heterocyclic fragments as potent anticancer agents against MCF-7, HepG2 and HCT-116. Inhibition of DNA topoisomerase II, induction of apoptosis, cell cycle arrest, and inhibition of cancer cell proliferation are the main hallmarks applied to estimate potent chemotherapies for their anticancer activities^41^.

There is a strong relation between apoptosis, inhibition of topoisomerase II and induced cell cycle arrest, in HepG2 Cells (Human Liver Cancer)^42^. Topoisomerase II expression in MCF-7 has been allied with HER2/neu protein overexpression and cell proliferation^43^. Moreover, human topoisomerase II catalytic inhibitors, inhibit DNA synthesis resulting in attenuation of cancer cell proliferation and DNA damage in HCT116 cells^44^. DNA-Topo II binding and docking evaluations of our novel derivatives were carried out. According to the main of DNA intercalators-topo II inhibitor pharmacophores, the new derivatives were designed.

### 1.1. Rationale and structure-based design

Our derivatives were obtained as quinoxaline chromophores having only a single side chain. Synthesis of our derivatives was performed by fusion of quinoxaline and triazole rings and joining of chalcones or pyrazole moieties to obtain the main chromophore with one side chain at position-4 as minor groove binder.

The new derivatives represent the chief structure requirements to intercalate DNA and also to inhibit the topo II enzyme. The triazoloquinoxaline chromophore is placed between DNA bases. Additionally, all designed derivatives contain basic nitrogen as cationic centres that enhance the selectivity and affinity towards DNA. Lastly, all derivatives have a single side chain to bind with the minor groove enhancing affinities. The selection of various substituents at different positions in the benzene ring was built on their relatively lipophilicity with different electron withdrawing or/and electron donating effects to enable us to investigate the final target SAR.

Overall, the designed derivatives were *in vitro* evaluated against MCF-7, HCT-166 and HepG2 for their anti-proliferative activities. The results provoked us to carry out further investigations into the mechanism of action of our derivatives. The most potent candidates were assessed for their capability to combine with DNA through DNA/methyl green and Topo II assay. Additionally, *in silico* studies were done to assess their affinities towards the active site of DNA.

## 2. Results and discussion

### 2.1. Chemistry

**Scheme 1.**
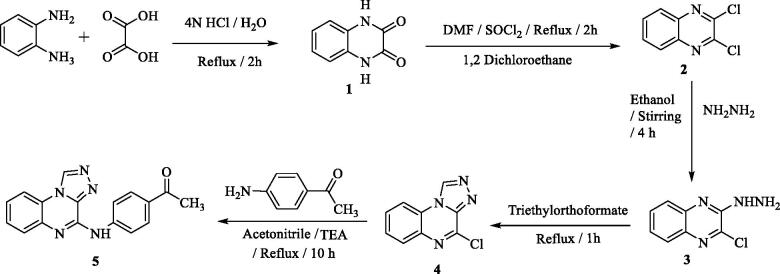
Target compounds **1–5** synthetic pathways.

**Scheme 2.**
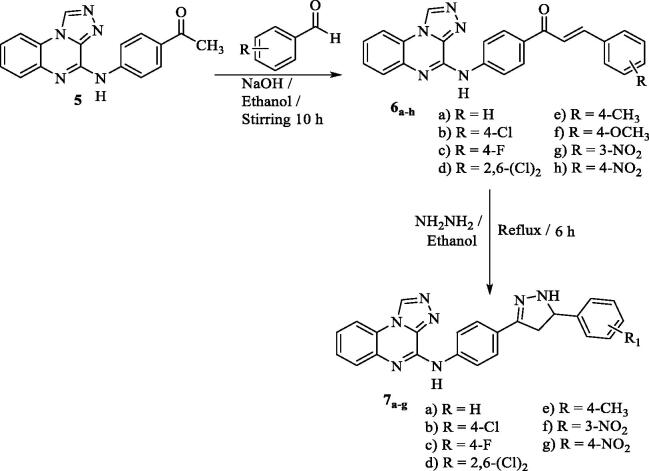
Target compounds **6** and **7a–g** synthetic pathway.

The reaction sequence for syntheses of our compounds is demonstrated in Schemes 1 and 2. Starting with the heating of benzene-1,2-diamine compounds **1–4** were obtained in agreement with reported methods following the reaction sequence mentioned in Schemes 1 and 2^21^^,^^22^. The heating of compound **4** with 4-aminoacetophenone under reflux afforded the acetyl derivative **5** (Scheme 1).

IR spectrum of **5** showed absorption bands at 3243, 2965, and 1725 cm^−1^ indicating NH, C-H aliphatic and C = O respectively. ^1^H NMR spectrum revealed new signals at δ 3.47 and 10.33 (D_2_O exchangeable) indicted CH_3_ and NH respectively. Heating the ketone derivative **5** with the appropriate aromatic aldehydes afforded the corresponding chalcones **(6a–h)**. On the other hand, cyclisation of the formed chalcones with hydrazine hydrate produced pyrazoles **7a–g** (Scheme 2). IR of compound **6f** displayed absorption bands at 1660 and 3111 cm^−1^ indicating the C = O group of α,β-unsaturated ketone and NH. ^1^H NMR proved the presence of OCH_3_ at *δ* 3.85 ppm. Furthermore, it confirmed the NH group at *δ* 10.67 ppm which disappeared when using D_2_O.

Also, IR of **7e** displayed C = O band disappearance and appearance of 2 NH bands at 3200 cm^−1^. The ^1^H NMR confirmed the presence of CH_3_ peak at *δ* 2.28. Also, two D_2_O exchangeable singlet peaks appeared at *δ* 10.24 and 10.36 ppm indicating 2NH.

### 2.2. Docking studies

**Figure 3.**
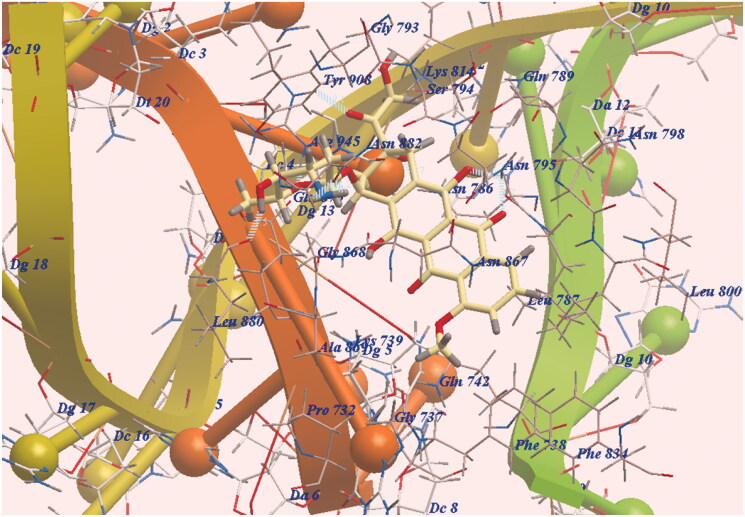
DNA- doxorubicin binding; H-B are illustrated with dashed lines (blue).

Molsoft program was used for docking our derivatives and doxorubicin on the binding site of DNA. It used top II complexes with DNA receptors (4G0U)^45^. The binding energy (ΔG) was presented in Table 1. The doxorubicin binding proposed mode showed exothermic energy = −100.31 kcal/mol and formed ten H-bonding interactions. The chromophore was placed in the hydrophobic groove formed by Ala869, Arg945, Asn786, Asn795, Asn867, Asn882, Gln742, Gln789, Gln870, Gly737, Gly868, Luc880, Lys739 and Phe738. It also formed two H-bonds with Asn795, one H-bond with Asn867 and one H-bond with Asn786. The sugar side chain was tilted towards DNA minor groove and formed one H-bond with Leu880 and two H-bonds with Asn882 and three H-bonds with Arg945 (Figure 3).

**Table 1.** Ligands binding affinity (ΔG in Kcal/mole).

Comp.	ΔG [kcal mol^−1^]	Comp.	ΔG [kcal mol^−1^]
**5**	−76.76	**7a**	−92.86
**6a**	−87.77	**7b**	−93.96
**6b**	−84.56	**7c**	−94.82
**6c**	−87.33	**7d**	−90.92
**6d**	−83.69	**7e**	−97.12
**6e**	−90.38	**7f**	−88.79
**6f**	−87.69	**7g**	−93.11
**6g**	−88.33	**Doxorubicin**	−100.31
**6h**	−87.94		

**Figure 4.**
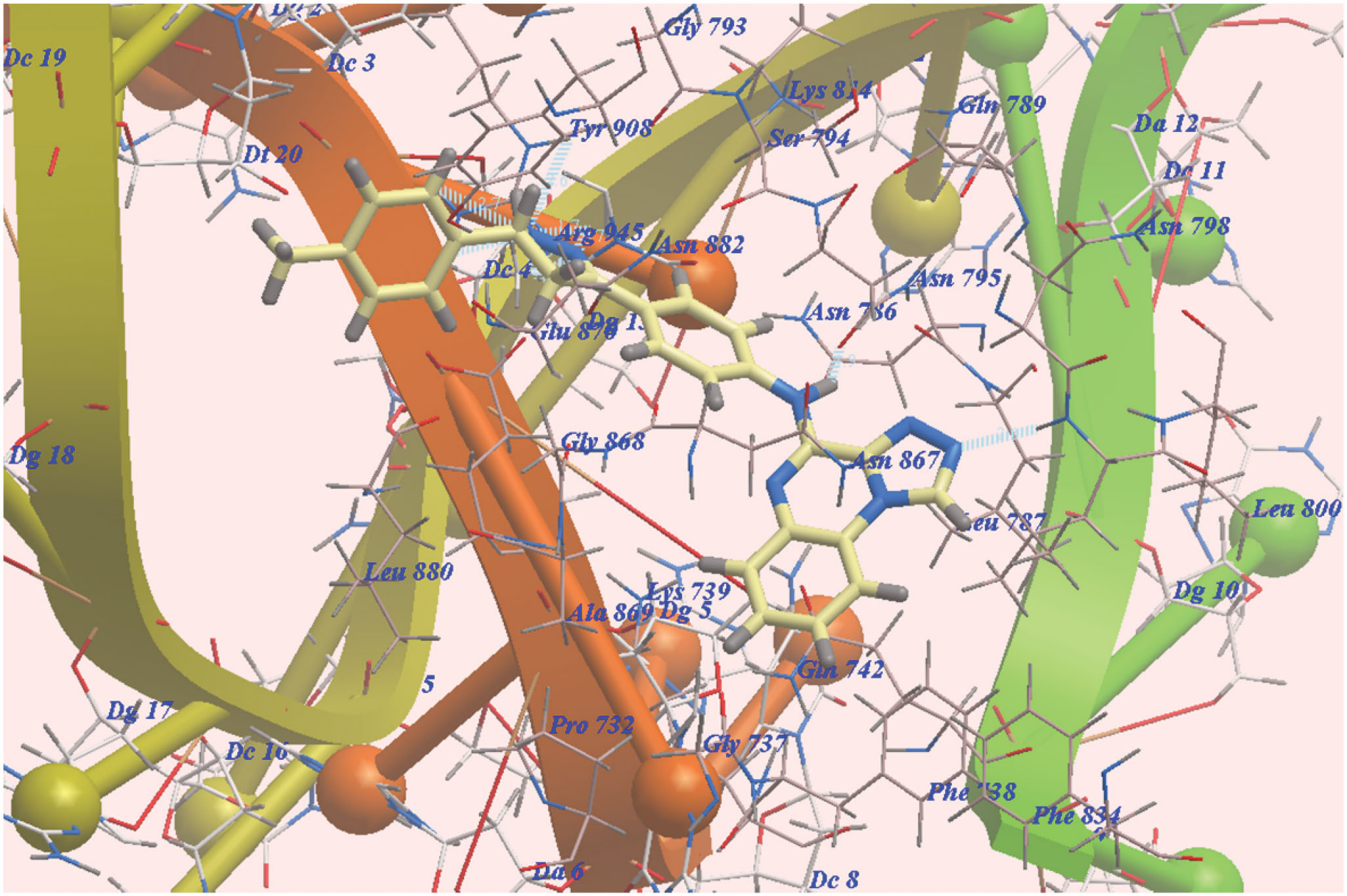
DNA-Topo II and **7e** expected binding mode.

**Figure 5.**
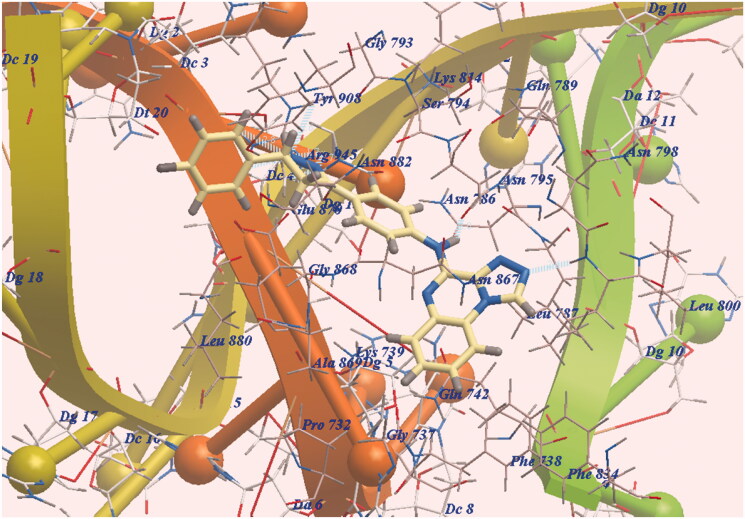
DNA-Topo II and **7c** expected binding mode.

**Figure 6.**
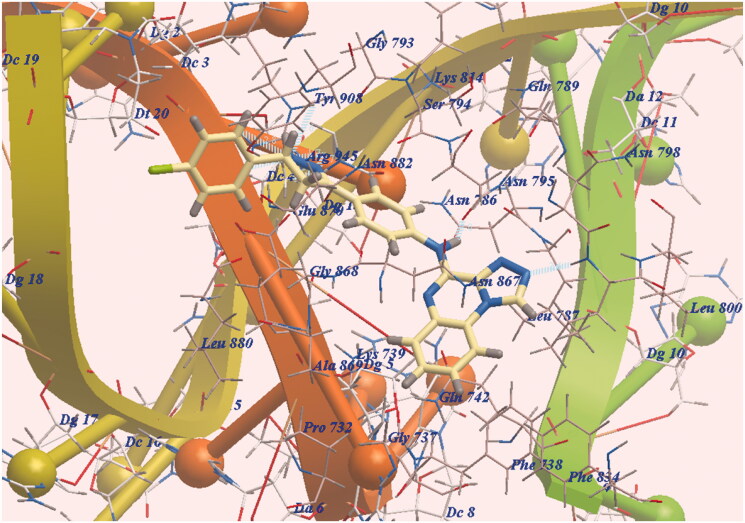
DNA-Topo II and **7b** expected binding mode.

The new derivative **7e** was docked in the same orientation as doxorubicin (−97.12 kcal/mol and eight Hydrogen bonds). The pharmacophore was presented in the same lipophilic channel as in the case of doxorubicin. Two H-bonds were formed with Leu799 and Asn795. The side chain was directed towards DNA minor groove and six H-bonds were formed with Arg945 (Figure 4). Additionally, the expected binding modes of **7c** (−94.82 kcal/mol and 8 H-bonding interactions (Figure 5)) and **7b** (−93.96 kcal/mol and H-bonding interactions (Figure 6)) have the same orientation and position as that of **7e**.

As scheduled, the chromophore HBA, the substituted distal phenyl and the long linkers enable our derivatives to act as DNA binders. Also, the pyrazoline moiety formed six H-bonds improving affinities with DNA active site. Finally, **7e**, **7c** and **7b** exhibited the highest DNA affinities and act as traditional intercalators of DNA.

### 2.3. MTT assessment

Assessment of cell multiplication inhibition action of quinoxaline derivatives **5, 6a–h** and **7a–g** were examined by means of MTT colorimetric assay against MCF-7, HCT-116 and HepG2^46–48^. Doxorubicin was used as a reference. The results were summarised in Table 2. New derivatives have the highest potent effect mainly on MCF-7. Compounds **7e** (IC_50_ = 6.15, 5.75, 3.41 µM), **7c (**IC_50_ = 6.33, 6.22, 4.45 µM) and **7b** (IC_50_ = 7.46, 6.90, 5.88 µM) displayed the greatest anticancer actions against HepG2, HCT116 and MCF-7 cell lines correspondingly and higher than doxorubicin, (IC_50_ = 7.94, 8.07 and 6.75 µM correspondingly).

**Table 2.** New derivatives in *vitro* cell growth inhibitory action.

Compound	IC_50_ (µM)^a^			
	HepG2	HCT116	MCF-7	VERO
**5**	12.74 ± 0.13	11.42 ± 1.4	10.30 ± 1.28	^b^ NT
**6a**	14.06 ± 0.13	11.17 ± 1.2	10.11 ± 1.31	^b^ NT
**6b**	35.22 ± 3.3	31.22 ± 1.4	25.82 ± 2.80	^b^ NT
**6c**	11.28 ± 1.1	9.53 ± 0.93	8.61 ± 0.62	^b^ NT
**6d**	16.93 ± 1.4	19.44 ± 1.7	14.61 ± 1.57	^b^ NT
**6e**	10.91 ± 1.9	10.16 ± 1.7	9.95 ± 0. 59	^b^ NT
**6f**	17.99 ± 1.6	16.41 ± 1.7	13.16 ± 1.39	^b^ NT
**6g**	17.17 ± 1.5	13.70 ± 1.7	12.16 ± 1.03	^b^ NT
**6h**	15.57 ± 1.2	12.40 ± 1.1	11.72 ± 1.68	^b^ NT
**7a**	20.33 ± 1.9	18.16 ± 1.6	15.36 ± 1.42	^b^ NT
**7b**	7.46 ± 0.13	6.90 ± 0.95	5.88 ± 0.74	46.32 ± 0.20
**7c**	6.33 ± 0.14	6.22 ± 0.63	4.45 ± 0.14	48.11 ± 0.20
**7d**	11.98 ± 1.2	10.19 ± 0.22	7.56 ± 0.92	^b^ NT
**7e**	6.15 ± 1.2	5.75 ± 0.37	3.41 ± 0.43	55.09 ± 0.16
**7f**	17.28 ± 1.9	16.24 ± 1.6	14.53 ± 1.03	^b^ NT
**7g**	9.51 ± 1.1	8.96 ± 0.37	8.62 ± 0.95	38.77 ± 0.16
**Doxorubicin**	7.94 ± 0.6	8.07 ± 0.8	6.75 ± 0.4	^b^ NT

^a^Three experiments were used to obtain the mean ± SD (IC_50_).

^b^NT = Not tested.

With respect to the HepG2 cell line, compound **7g** exhibited exceptional anticancer activities (IC_50_ = 9.51 µM). Compounds **5**, **6a**, **6c–h, 7d** and **7f** displayed very good anticancer activities (IC_50_ from 10.91 to 17.99 µM). Derivative **7a** (IC_50_ = 20.33 µM), demonstrated potent cytotoxic effect. However **6b** (IC_50_ = 35.22 µM) demonstrated moderate cytotoxic action.

HCT-116 cytotoxicity evaluation discovered that compounds **7g** and **6c** showed significant cytotoxic effects against HCT-116 (IC_50_ = 8.96 and 9.53 and µM respectively. Also, **5**, **6a**, **6d–h, 7a**, **7d** and **7f** displayed very good anticancer actions (IC_50_ ranging from 10.16 to 19.44 µM). However **6b** (IC_50_ = 31.22 µM) demonstrated medium cytotoxic effect.

MCF-7 cytotoxicity assessment showed that compounds **7d, 6c, 7g** and **6e** presented potent anticancer actions (IC_50_ = 7.56, 8.61, 8.62 and 9.95 µM). Compounds **5**, **6a**, **6d, 6f, 6g, 6h, 7a** and **7f** displayed very good anticancer effects (IC_50_ from 10.11 to 15.36 µM). While, compound **6b** with IC_50_ = 25.82 µM, displayed good cytotoxicity.

Finally, the four most potent derivatives **7b, 7c, 7e** and **7g** were assessed for their cytotoxicity against VERO normal cell lines. The results discovered that the new derivatives displayed low toxicity against VERO normal cells with IC_50_ values = 38.77–55.09 µM. The cytotoxicity of these compounds against the cancer cell lines was from 3.41 to 9.51 µM. Derivatives **7b, 7c, 7e** and **7g** are respectively, 7.88, 10.81, 16.16 and 4.50 fold safer in VERO normal cells compared to breast cancer cell lines (MCF-7, the most sensitive cells).

### 2.4. *In vitro* DNA binding evaluation

The extremely potent **6e, 7b, 7c, 7e** and **7g** were further assessed for their DNA-binding according to the reported procedure using methyl green dye^21^^,^^22^^,^^49^. DNA-binding affinities results were presented as IC_50_ and briefed in Table 3. All results were compared to doxorubicin.

**Table 3.** The most potent derivatives; Topoisomerase II inhibitory activity and DNA binding affinity.

Compound	DNA binding IC_50_ (µM)	Topo-II assay IC_50_ (µM)
**6e**	38.00 ± 0.40	1.275 **±** 0.40
**7b**	32.49 ± 3.0	1.050 **±** 0.40
**7c**	31.24 ± 2.9	0.940 **±** 0.40
**7e**	29.06 ± 2.2	0.890 ± 0.40
**7g**	36.50 ± 0.40	1.220 **±** 0.40
**Doxorubicin**	31.27 ± 1.8	0.940 **±** 0.40

^a^IC_50_ values are the mean ± SD of three separate experiments.

Our new derivatives elicit excellent to very good effects as DNA binders. **7e** is the highly potent one. It intercalates nucleic acid at lower IC_50_ (29.06 µM). Moreover, compound **7e** potently intercalates DNA, at an IC_50_ value of 31.24 µM compared to doxorubicin (31.27 µM). Also **6e**, **7b** and **7g** bind to DNA with high affinities at IC_50_ values of 38.00, 32.49 and 36.50 µM, respectively.

### 2.5. *In vitro* topoisomerase II inhibitory activity

The greatest active compounds **6e, 7b, 7c, 7e** and **7g** also were evaluated as Topo II enzyme inhibitors agreeing with the stated procedure^21^^,^^22^. All results were compared to the reference drug doxorubicin (Table 3). All derivatives exhibited excellent or very good inhibition activities (IC_50_ range 0.890–1.275 µM) in comparison with doxorubicin (IC_50_ = 0.94 µM). The obtained results were matched with molecular docking studies, DNA binding and *in vitro* cytotoxicity activities. Compound **7e** was found to be the most potent derivative at IC_50_ value of 0.890 µM. Also, compound **7c** exhibited equipotent IC_50_ = 0.940 µM to that of doxorubicin, while compounds **6e**, **7b** and **7g** displayed significant Topo II inhibitory activities with IC_50_ of 1.275, 1.050 and 1.220 µM, correspondingly.

### 2.6. SAR (structure activity relationship)

The SAR has concentrated on the impact of length and type of linkers, position of the substituents at benzene ring electronic and hydrophobic nature. All derivatives showed variable activity levels with characteristic MCF-7 selectivity. The distal hydrophobic phenyls attached to [1, 2, 4]triazolo[4,3-a]quinoxalines chromophore through the novel linkers; prop-2-en-1-one and/or pyrazoline linkers containing (HBA-HBD). These linkers, the substituents lipophilicity and their electronic nature exhibited an essential role in anticancer activity as DNA intercalators. The pyrazoline linker as in **7a–g** showed higher activities than the prop-2-en-1-one linker as in **6a–h**.

The tested derivatives are classified into two groups. The first one is compounded **6a–h**. In this group, prop-2-en-1-one linker was used. Compound **6e** containing distal phenyl moiety substituted with hydrophobic, electron donating (+I) methyl group exhibited higher anticancer activity than **6d** that substituted with hydrophobic, electron withdrawing fluoro group with + M and -I and **6a** that unsubstituted against HepG2 cell lines, while compound **6d** displayed higher anticancer activity than **6c** and **6a** against the two MCF-7 and HCT116 cell lines respectively. Compound **6h** substituted with 4-nitro group (−M, −I) exhibited higher activities than **6g** that 3-substituted one against the three HepG2, HCT116 and MCF-7 cell lines. This indicated that the 4-position is essential for higher activity. Derivative **6d** having 2,6-dichloro (+M, −I) exhibited higher activities than mono substituted one **6b** against MCF-7, HCT116 and HepG2. **6f** that containing the 4-methoxy (+M, +I) group displayed higher activities than **6b** with 4-chloro (+M, −I) one against the three cancer lines.

**7a–g** derivatives constitute the second group. **6e** with 4-methyl substituent showed higher activities than **6d** with 4-fluoro group (+M, −I) and **6b** with 4-chloro (+M, −I) substituent against the three cancer lines. **7d** with 2,6-dichloro substituents resulted in inferior activities to that of 7**b** with a mono substituent. Compound **6h** with a 4-nitro group (−M, −I) exhibited higher activities than **6g** with 3-nitro one against the three cancer cell lines. **6a** containing unsubstituted phenyl group showed the lowest anticancer activities against the three cell lines. These findings are consistent with the parabolic relationship of the Hansch equation.

### 2.7. *In silico* ADMET calculations

Compounds **7b, 7c, 7e** and **7g** were exposed to a computational study to determine the physicochemical properties according to the rule of Lipinski^50^. He recommended good absorption of a ligand if it at least accomplishes three rules of the following: (1) Hydrogen bond donors are not more than five; (2) Hydrogen bond acceptors are not more than 10; (3) Molecular weight less than 500; (4) Partition coefficient (logP) is not more than 5. In the present study, while doxorubicin missed three rules, only compound **7b** exceeds the rule of molecular weight by a small fraction. ADMET prediction was performed online using the algorithm protocol of the pkCSM descriptor (http://biosig.unimelb.edu.au/pkcsm/prediction)^51^. Evaluation of the ADMET properties of **7b, 7c, 7e** and **7g** (Table 4) displayed better absorption (91.581–97.215) compared to doxorubicin (62.3). This preference may be attributed to the high degree of hydrophobicity of our derivatives^52^. Moreover, **7b, 7c, 7e** and **7c** showed good CNS penetration (−1.707 to −2.037), compared to the inability of doxorubicin to cross CNS (<−4.0). On the other hand **7b, 7c, 7e** and **7g** can inhibit CYP3A4 metabolic enzymes but doxorubicin cannot. Calculation of excretion for our derivatives exhibited lower rates in comparison to doxorubicin. So it showed longer duration of action. Regarding the humans' maximum tolerated dose, our quinoxaline derivatives **7b, 7c, 7e** and **7g** showed 0.336, 0.329, 0.332 and 0.299, respectively while 0.081 for doxorubicin. So our directives have a broad therapeutic window. It is also indicated by higher LD_50_ of our derivatives (2.617–2.660) in comparison to 2.408 for doxorubicin.

**Table 4.** *In silico* ADMET calculations.

Parameter	7b	7c	7e	7g	Doxorubicin
**Molecular properties**					
Mol. Weight	439.91	423.455	419.492	450.462	543.525
LogP	5.1132	4.5989	4.76822	4.368	0.0013
Rotatable bonds	4	4	4	5	5
Acceptors	7	7	7	9	12
Donors	2	2	2	2	6
Surface area	188.330	182.193	184.392	192.680	222.081
**Absorption**					
Water solubility	−3.901	−3.864	−3.92	−3.868	−2.915
Human Intest. absorption	91.581	92.483	93.039	97.215	62.372
Permeability throughout skin	−2.736	−2.736	−2.736	−2.735	−2.735
**Distribution**					
Permeability throughout BBB	−0.727	−0.759	−0.552	−0.82	−1.379
Permeability to CNS	−1.707	−1.861	−1.748	−2.037	−4.307
**Metabolism**					
Inhibition of CYP2C9	+	+	+	+	−
Inhibition of CYP2D6	−	−	−	−	−
Inhibition of CYP3A4	+	+	+	+	−
**Excretion**					
Clearance	0.058	−0.062	0.144	0.083	0.987
**Toxicity**					
AMES toxicity	+	+	+	+	−
Hum. Maximum tol. dose	0.336	0.329	0.332	0.299	0.081
Acute toxic activity	2.659	2.660	2.662	2.617	2.408
Chronic toxic activity	1.73	1.812	1.683	2.391	3.339
Hepatotoxic effect	+	+	+	+	+
Minnow toxic activity	−0.066	0.267	0.151	−1.055	4.412

## 3. Conclusion

In summary, new series of DNA intercalators and Topo II inhibitors derived from quinoxalines have been synthesised. Their anti-proliferative activities were estimated against three different types of cancer. A docking study was carried out to evaluate their DNA-binding activity. Docking data was highly related to that biological testing.

MCF-7 was the most affected one by our derivatives influence. Compounds **7e (**IC_50_ = 6.15, 5.75, 3.41 µM**)**, **7c (**IC_50_ = 6.33, 6.22, 4.45 µM**)** and **7b** (IC_50_ = 7.46, 6.90, 5.88 µM) demonstrated the highest anti-proliferative actions against HepG2, HCT116 and MCF-7 correspondingly. These compounds presented higher activities than that of doxorubicin, (IC_50_ = 7.94, 8.07 and 6.75 µM correspondingly). Compounds **7g** and **6e** revealed very high anti- proliferative activities against HepG2, HCT116 and MCF-7 cancers with (IC_50_ = 9.51, 8.96 and 8.62 µM) and (IC_50_ = 10.91, 10.16 and 9.95 µM) respectively. The greatest active compounds **7e**, **7c, 7b, 7g** and **6e** were estimated for their DNA-binding and Topo II inhibition activities. Compound **7e** displayed the highest binding affinity. This compound potently intercalates DNA at decreased IC_50_ value (29.06 µM). Finally, compound **7e** showed the greatest potency as a Topo II inhibitor at IC_50_ = 0.890 µM. Docking results concluded that our derivatives **7e**, **7c** and **7b** demonstrated the highest activities as classical DNA intercalators. The pyrazoline moiety formed six H-bonds and increased affinities towards DNA active site. Furthermore, our derivatives **7b, 7c, 7e** and **7g** displayed wonderful *in Silico* predicted ADMET profile.

## 4. Experimental

### 4.1. Chemistry

#### 4.1.1. General

Derivatives **1–4** were prepared according to the reported methods^21^^,^^22^. ^1^H NMR and ^13 ^C NMR for all derivatives were done on a Bruker at 400 and 100 MHz using DMSO-d_6_ solvent and represented on the *δ* ppm scale at Cairo university Microanalytical unit. Thin layer chromatography (TLC) was used to monitor the reactions.

##### 4.1.1.1. 1-{4-([1, 2, 4]Triazolo[4,3-a]quinoxalin-4-ylamino)phenyl}ethan-1-one (5)

Derivative **4** (4.08 g, 0.02 mol) and 4-aminoacetophenone (3.02 g, 0.02 mol) were heated under reflux in CH_3_CN (20 ml) with 0.5 ml of TEA for 10 h. The precipitated product was filtered and washed with *n*-hexane then dried.

Yield, 91%; m.p. 171–172 °C; IR_νmax_ (cm^−1^): 3243 (NH), 3090 (C-H aromatic), 2965 (C-H aliphatic), 1725 (CO); ^1^H NMR at 2.50 (s, 3H, CH_3_), 7.45 (dd, 1H, quin H-7), 7.51 (dd, 1H, quin H-8), 7.74 (d, 1H, quin H-9), 7.94 (dd, 2H, phenyl H-5,3), 8.21 (d, 1H, quin H-6.), 8.34 (dd, 2H, phenyl H-6,2), 10.01 (s, 1H, CH triazolo), 10.58 (s, 1H, Ph-NH, D_2_O exchangeable); ^13 ^C NMR, 26.91 (C, CH_3_), 116.72 (CH, quin C-9), 120.03 (phenyl C-3, 5), 123.22 (quin C-1, 10), 125.83 (quin C-9), 127.60 (quin C-9), 128.19 (quin C-7), 129.66 (phenyl C-2, 6)), 131.61 (phenyl C-4), 136 (quin C-3), 138.78 (triazolo C-3), 143.32 (phenyl C-1), 144 0.64 (quin C-4), 196.92 (C = O amidic); MS (m/z): 305.02 (M^+^+2, 6.32%), 304.03 (M^+^+1, 24.15%), 303 (M^+^, base beak, 100%), 302 (64.34%), 287 (78.78%), 89 (87.43%), 75 (67.97%); Anal. Calcd. for C_17_H_13_N_5_O_2_ (303.33): C, 67.32; H, 4.32; N, 23.09. Found: C, 67.53; H, 4.46; N, 23.24.

#### 4.1.2. *General methods for preparation of target derivatives* (6a–h)

Ketone **5** (1.0 g, 0.003 mol) and the appropriate aromatic aldehyde (0.0045 mol) were heated under reflux in ethanol (10 ml), ethanolic NaOH (10 ml, 10%) was added dropwise within 15 min. The reaction mixture was stirred for 10 h, the precipitate was filtered, air dried and crystallised from ethyl alcohol to give the corresponding chalcones **6a–h**.

##### 4.1.2.1. 1-{4-([1, 2, 4]Triazolo[4,3-a]quinoxalin-4-ylamino)phenyl}-3-phenylprop-2-en-1-one (6a)

Yield, 80%; m.p. 275–277 °C; IR_νmax_ (cm^−1^): 3280 (NH), 3101 (C-H aromatic), 1630 (CO of α,β-unsaturated ketone); ^1^H NMR 7.46–7.56 (m, 5H, H-2′, 3′,4′, 5′,6′), 7.56–7.59 (m, 1H, quin H-7.), 7.71–7.74 (m, 1H, quin H-8), 7.79–7.81 (d, 1H, quin H-9), 7.99–8.03 (m, 2H, Phenyl H-3,5), 8.22–8.24 (d, 1H, quin H-6), 8.28 (dd, 2H, Phenyl H-3,6), 8.4 (dd, 2H, CO-CH = CH-ph), 10.08 (s, 1H, triazolo CH), 10.69 (s, 1H, NH-ph) (D_2_O exchangeable); ^13 ^C NMR 116.73 (quin C-9), 120.40 (phenyl C-3,5), 122.63 (quin C1,10), 123.20 (CH = CH-ph), 125.57 (quin C-8), 127.46 (quin C-6), 128.21 (quin C-7), 129.27 (phenyl C-4), 129.38 (phenyl C-2,6), 130.14 (5 CH, C-2′, 3′, 4′, 5′, 6′), 130.88 (C, C-1′), 135.37 (quin C-3), 138.71 (triazole C-3), 143.36 (phenyl C-1), 143.73 (quin C-4), 187.84 (C, C = O amidic); MS (m/z): 392 (M^+^, 31.52%), 390 (56.03%), 305 (31.33%), 303 (63.12%), 126 (base beak, 100%); Anal. Calcd. for C_24_H_17_N_5_O (391.43): C, 73.64; H, 4.38; N, 17.89. Found: C, 73.87; H, 4.59; N, 18.12.

##### 4.1.2.2. 1-{4-([1, 2, 4]Triazolo[4,3-a]quinoxalin-4-ylamino)phenyl}-3–(4-chlorophenyl)prop-2-en-1-one (6b)

Yield, 85%; m.p. 240–243 °C; IR_νmax_ (cm^−1^): 3197 (NH), 3031 (C-H aromatic), 1643 (CO of α,β-unsaturated ketone); ^1^H NMR 7.50 (dd, 2H, H-3′,5′), 7.65–7.68 (m, 1H, quin H-7.), 7.69–7.71 (m, 1H, quin H-8.), 7.74 (d, 1H, quin H-9), 7.90–7.92 (m, 2H, Phenyl H-3, 5), 7.99 (d, 1H, quin H-6), 8.01–8.03 (m, 2H, phenyl H-2,6), 8.20–8.22 (m, 2H, H-2′, 6′), 8.39–8.41 (m, 2H, CO-CH = CH-ph), 10.05 (s, 1H, triazole CH), 10.66 (s, 1H, NH-ph, D_2_O exchangeable); MS (m/z): 427 (M^+^+2, 7.51%), 425 (M^+^, 22.63%), 370 (94.93%), 304 (92.81%), 299 (74.16%), 238 (71.42%), 69.01 (base beak, 100%); Anal. Calcd. for C_24_H_16_ClN_5_O (425.88): C, 67.69; H, 3.79; N, 16.44. Found: C, 68.01; H, 3.85; N, 16.72.

##### 4.1.2.3. 1-{4-([1, 2, 4]Triazolo[4,3-a]quinoxalin-4-ylamino)phenyl}-3–(4-fluorophenyl)prop-2-en-1-one (6c)

Yield, 85%; m.p. 247–249 °C; IR_νmax_ (cm^−1^): 3237 (NH), 3071 (C-H aromatic), 1632 (CO of α,β-unsaturated ketone); ^1^H NMR 7.29–7.31 (m, 2H, H-3′, 5′), 7.47–7.50 (m, 1H, quin H-7.), 7.51–7.53 (m, 1H, quin H-8), 7.55 (d, 1H, quin H-9), 7.74–7.76 (m, 2H, phenyl H-3,5), 7.96–7.98 (m, 2H, phenyl H-2,6), 8.02 (d, 1H, quin H-6), 8.21–8.23 (m, 1H, H-2′,6′), 8.43–8.45 (dd, 2H, CO-CH = CH-ph), 10.07 (s, 1H, triazole CH), 10.67 (s, 1H, NH-phenyl); Anal. Calcd. for C_24_H_16_FN_5_O (409.42): C, 70.41; H, 3.94; N, 17.11. Found: C, 70.35; H, 4.12; N, 17.38.

##### 4.1.2.4. 1-{4-([1, 2, 4]Triazolo[4,3-a]quinoxalin-4-ylamino)phenyl}-3–(2,6-dichlorophenyl)prop-2-en-1-one (6d)

Yield, 90%; m.p. 260–263 °C; IR_νmax_ (cm^−1^): 3288 (NH), 3100 (C-H aromatic), 1638 (CO of α,β-unsaturated ketone); ^1^H NMR 7.42 (t, 1H, H-4′), 7.47–7.50 (m, 1H, quin H-7.),7.52–7.55 (m, 1H, quin H-8), 7.57 (d, 1H, quin H-9), 7.75–7.77 (m, 2H, phenyl H-3,5), 7.82 (d, 1H, quin H-6.), 8.08 (dd, 2H, H phenyl H-2,6), 8.21 (dd, 2H, H-3′, 5′), 8.41 (dd, 2H, CO-CH = CH-phenyl), 10.04 (s, 1H, triazole CH), 10.69 (s, 1H, NH-phenyl, D_2_O exchangeable); Anal. Calcd. for C_24_H_15_Cl_2_N_5_O (460.32): C, 62.62; H, 3.28; N, 15.21. Found: C, 62.87; H, 3.41; N, 15.38.

##### 4.1.2.5. 1-{4-([1, 2, 4]Triazolo[4,3-a]quinoxalin-4-ylamino)phenyl}-3-(p-tolyl)prop-2-en-1-one (6e)

Yield, 80%; m.p. 245–247 °C; IR_νmax_ (cm^−1^): 3280 (NH), 3111 (C-H aromatic), 2995 (C-H aliphatic), 1639 (CO of α,β-unsaturated ketone); ^1^H NMR 2.36 (s, 3H, CH_3_), 7.25–7.29 (m, 2H, H-3′,5′), 7.46–7.50 (m, 2H, H-2′, 6′), 7.63–7.66 (m, 1H, quin H-7), 7.67–7.70 (m, 1H, quin H-8), 7.74 (d, 1H, quin H-9), 7.90–7.92 (m, 2H, phenyl H-3,5), 7.94 (d, 1H, quin H-6), 8.19 (dd, 2H, phenyl H-2,6), 8.40 (dd, 2H, CO-CH = CH-phenyl), 10.05 (s, 1H, triazole CH), 10.64(s, 1H, NH, D_2_O exchangeable); Anal. Calcd. for C_25_H_19_N_5_O (405.46): C, 74.06; H, 4.72; N, 17.27. Found: C, 74.28; H, 4.83; N, 17.59.

##### 4.1.2.6. 1-{4-([1, 2, 4]Triazolo[4,3-a]quinoxalin-4-ylamino)phenyl}-3–(4-methoxyphenyl)prop-2-en-1-one (6f)

Yield, 75%; m.p. 250–253 °C; IR_νmax_ (cm^−1^): 3111(NH), 3002 (C-H aromatic), 2900 (C-H aliphatic), 1660 (CO of α,β-unsaturated ketone); ^1^H NMR 3.85 (s, 3H, CH_3_), 7.01–7.05 (m, 2H, H-3′, 5′), 7.46–7.49 (m, 1H, quin H-7),7.51–7.54 (m, 1H, quin H-8), 7.58 (d, 1H, quin H-9), 7.73–7.75 (m, 2H, phenyl H-3,5), 7.89 (d, 1H, quin H-6.), 8.21 (dd, 2H, phenyl H-2,6), 8.27 (dd, 2H, H-2′, 6′), 8.41 (dd, 2H CO-CH = CH-phenyl), 10.06 (s, 1H,triazole CH), 10.67 (s, 1H, NH-phenyl, D_2_O exchangeable); MS (m/z): 421.57 (M^+^, 31.87%), 376.34 (71.52%), 301.94 (59.70%), 274.80 (39.02%), 165.38 (M^+^, base beak, 100%), 135.62 (55.11%); Anal. Calcd. for C_25_H_19_N_5_O_2_ (421.46): C, 71.25; H, 4.54; N, 16.62. Found: C, 71.52; H, 4.69; N, 16.91.

##### 4.1.2.7. 1-{4-([1, 2, 4]Triazolo[4,3-a]quinoxalin-4-ylamino)phenyl}-3–(3-nitrophenyl)prop-2-en-1-one (6g)

Yield, 80%; m.p. 257–259 °C; IR_νmax_ (cm^−1^): 3220 (NH), 3001 (C-H aromatic), 1643 (CO of α,β-unsaturated ketone); ^1^H NMR 7.53 (dd, 2H, H-4′, 5′), 7.72–7.75 (m, 1H, quin H-7),7.78–7.81 (m, 1H, quin H-8), 7.83 (d, 1H, quin H-9), 8.18 (d, 1H, H-6′), 8.24 (dd, 2H, phenyl H-3,5), 8.27 (d, 1H, quin H-6.), 8.32 (dd, 2H, phenyl H-2,6), 8.45 (dd, 2H, CO-CH = CH-phenyl), 8.77 (d, 1H, H-2′), 10.07 (s, 1H, triazole CH), 10.69 (s, 1H, NH-phenyl); ^13 ^C NMR, 116.79 (quin C-9.), 120.13 (phenyl C-3,5), 123.26 (quin C-1,10), 124.86 (CH = CH-phenyl), 125.24 (C-2′), 125.76 (quin C-8), 127.50 (quin C-6), 128.17 (quin C-7.), 129.77 (phenyl C-4), 130.33 (phenyl C-2,6), 130.71 (C-4′), 131.76 (C-1′), 135.50 (quin. C-3), 136.14 (C-3′), 137.22 (C-5′), 138.87 (triazole C-3), 143.37 (phenyl C-1), 145.26 (quin C-4), 148.84 (C-6′), 187.56 (C = O amidic); Anal. Calcd. for C_24_H_16_N_6_O_3_ (436.43): C, 66.05; H, 3.70; N, 19.26. Found: C, 66.32; H, 3.89; N, 19.43.

##### 4.1.2.8. 1-{4-([1, 2, 4]Triazolo[4,3-a]quinoxalin-4-ylamino)phenyl}-3–(4-nitrophenyl)prop-2-en-1-one (6h)

Yield, 90%; m.p. 261–263 °C; IR_νmax_ (cm^−1^): 3120 (NH), 3000 (C-H aromatic), 1633 (CO of α,β-unsaturated ketone); ^1^H NMR 7.50 (dd, 2H, H-3′, 5′), 7.77–7.80 (m, 1H, quin H-7),7.81–7.84 (m, 1H, quin H-8.), 7.86 (d, 1H, quin H-9), 8.19 (dd, 2H, phenyl H-3,5), 8.25 (d, 1H, quin H-6), 8.28 (dd, 2H, phenyl H-2,6), 8.31 (dd, 2H, H-2′, 6′), 8.46 (dd, 2H, CO-CH = CH-phenyl), 10.09 (s, 1H, triazole CH), 10.72 (s, 1H, NH phenyl, D_2_O exchangeable); Anal. Calcd. for C_24_H_16_N_6_O_3_ (436.43): C, 66.05; H, 3.70; N, 19.26. Found: C, 65.97; H, 3.86; N, 19.57.

#### 4.1.3. *General method for preparation of target derivatives* 7a–g

Chalcones **6a–e,g,h** (0.001 mol) and hydrazine hydrate 80% (0.5 g, 0.01 mol) in ethanol (15 ml) were heated under reflux for 6 h, then left at rt for 12 h. The precipitate was washed several times with water, dried and crystallised from ethanol to afford the corresponding pyrazoles **7a–g.**

##### 4.1.3.1. *N*-{4–(5-Phenyl-4,5-dihydro-1*H*-pyrazol-3-yl)phenyl}-[1, 2, 4]triazolo[4,3-a]quinoxalin-4-amine (7a)

Yield, 75%; m.p. 150–152 °C; IR_νmax_ (cm^−1^): 3299 (2NH), 3069 (C-H aromatic), disappearance of the absorption band for CO of chalcone); ^1^H NMR 2.85–2.87 (m, 2H, pyrazole CH_2_), 4.84 (t, 1H, pyrazole CH), 7.16–8.44 (m, 13H, aromatic protons), 10.04 (s, 1H, triazole CH), 10.24 (s, 1H, NH pyrazole), 10.61 (s, 1H, NH-phenyl, D_2_O exchangeable); MS (m/z): 405 (M^+^, 23.58%), 392 (19.74%), 311 (75.84%), 262 (base beak, 100%), 180 (56.48%); Anal. Calcd. for C_24_H_19_N_7_ (405.47): C, 71.09; H, 4.72; N, 24.18. Found: C, 71.23; H, 4.89; N, 24.39.

##### 4.1.3.2. *N*-{4-[5–(4-Chlorophenyl)-4,5-dihydro-1*H*-pyrazol-3-yl]phenyl}-[1, 2, 4]triazolo[4,3-a]quinoxalin-4-amine (7b)

Yield, 80%; m.p. 160–162 °C; IR_νmax_ (cm^−1^): 3209 (2NH), 3060 (C-H aromatic), disappearance of the absorption band for CO of chalcone); MS (m/z): 441 (M^+^ +2, 3.35%), 439.55 (M^+^, 9.06%), 319 (44.71%), 289 (68.98%), 287 (base beak, 100%), 66.15 (28.80%); Anal. Calcd. for C_24_H_18_ClN_7_ (439.91): C, 65.53; H, 4.12; N, 22.29. Found: C, 66.01; H, 3.90; N, 22.01.

##### 4.1.3.3. *N*-{4-[5–(4-Fluorophenyl)-4,5-dihydro-1*H*-pyrazol-3-yl] phenyl}-[1, 2, 4]triazolo[4,3-a]quinoxalin-4-amine (7c)

Yield, 80%; m.p. 186–188 °C; IR_νmax_ (cm^−1^): 3200 (2NH), 3009 (C-H aromatic), disappearance of the absorption band for CO of chalcone); ^1^H NMR 2.84–286 (m, 2H, pyrazole CH_2_), 4.85 (t, 1H, pyrazole CH), 7.17–8.44 (m, 12H, aromatic protons), 10.05 (s, 1H, triazole CH) 10.24 (s, 1H, NH pyrazole), 10.63 (s, 1H, NH-phenyl, D_2_O exchangeable); MS (m/z): 423 (M^+^, C_24_H_18_FN_7_, 31.31%), 369 (35.70%), 327 (base beak, 100%), 305 (74.25%), 148 (62.75%), 55 (98.86%); Anal. Calcd. for C_24_H_18_FN_7_ (423.46): C, 68.07; H, 4.28; N, 23.15. Found: C, 68.24; H, 4.39; N, 23.53.

##### 4.1.3.4. *N*-{4-[5–(2,6-Dichlorophenyl)-4,5-dihydro-1*H*-pyrazol-3-yl] phenyl}-[1, 2, 4]triazolo[4,3-a]quinoxalin-4-amine (7d)

Yield, 85%; m.p. 176–178 °C; IR_νmax_ (cm^−1^): 3180 (2NH), 3050 (C-H aromatic), disappearance of the absorption band for CO of chalcone); ^1^H NMR 3.24–3.26 (m, 1H, CH_2_ pyrazole), 3.48–3.50 (m, 1H, CH pyrazole), 5.59 (t, 1H, pyrazole CH), 7.31–8.26 (m, 11H, aromatic protons), 10.06 (s, 2H, triazole CH & NH pyrazole, D_2_O exchangeable), 10.38 (s, 1H, **NH**-phenyl, D_2_O exchangeable); Anal. Calcd. for C_24_H_17_Cl_2_N_7_ (474.35): C, 60.77; H, 3.61; N, 20.67. Found: C, 60.50; H, 3.42; N, 20.99.

##### 4.1.3.5. *N*-{4-[5-(*p*-Tolyl)-4,5-dihydro-1*H*-pyrazol-3-yl]phenyl}-[1, 2, 4]triazolo[4,3-a]quinoxalin-4-amine (7e)

Yield, 75%; m.p. 190–192 °C; IR_νmax_ (cm^−1^): 3200 (2NH), 3069 (C-H aromatic), 2927 (C-H aliphatic), disappearance of the absorption band for CO of chalcone); ^1^H NMR 2.28 (s, 3H, CH_3_), 2.84–2.86 (m, 2H, CH_2_ pyrazole), 4.80 (t, 1H, pyrazole, CH), 7.15–8.23 (m, 12H, aromatic proton), 10.04 (s, 1H, CH triazole CH), 10.24 (s, 1H, NH pyrazole D_2_O exchangeable), 10.36 (s, 1H, NH phenyl, D_2_O exchangeable); Anal. Calcd. for C_25_H_21_N_7_ (419.49): C, 71.58; H, 5.05; N, 23.37. Found: C, 71.79; H, 5.11; N, 23.52.

##### 4.1.3.6. *N*-{4-[5–(3-Nitrophenyl)-4,5-dihydro-1*H*-pyrazol-3-yl]phenyl} -[1, 2, 4]triazolo[4,3-a]quinoxalin-4-amine (7f)

Yield, 80%; m.p. 195–197 °C; IR_νmax_ (cm^−1^): 3259 (2NH), 3087 (C-H aromatic), disappearance of the absorption band for CO of chalcone); ^1^H NMR 2.99–3.01 (m, 2H, CH_2_ pyrazole), 4.29 (t, 1H, pyrazole CH), 7.46–8.26 (m, 12H, aromatic proton), 10.06 (s, 1H, CH triazole CH) 10.28 (s, 1H, 2NH); Anal. Calcd. for C_24_H_18_N_8_O_2_ (450.46): C, 63.99; H, 4.03; N, 24.88. Found: C, 64.35; H, 4.25; N, 25.12.

##### 4.1.3.7. *N*-{4-[5–(4-Nitrophenyl)-4,5-dihydro-1*H*-pyrazol-3-yl]phenyl} -[1, 2, 4]triazolo[4,3-a]quinoxalin-4-amine (7g)

Yield, 85%; m.p. 203–205 °C; IR_νmax_ (cm^−1^): 3277 (2NH), 3039 (C-H aromatic), disappearance of the absorption band for CO of chalcone); ^1^H NMR 3.05–307 (m, 2H, CH_2_ pyrazole), 4.29 (t, 1H, pyrazole CH), 7.45–8.23 (m, 12H, aromatic proton), 10.04 (s, 1H, triazole CH) 10.26 (s, 2H, NH pyrazole & NH-phenyl, D_2_O exchangeable); Anal. Calcd. for C_24_H_18_N_8_O_2_ (450.46): C, 63.99; H, 4.03; N, 24.88. Found: C, 64.34; H, 4.12; N, 25.07.

### 4.2. Docking studies

Docking experiments were done using molsoft program. Each experiment used DNA-Top II (https://www.rcsb.org/structure/4G0U) downloaded from Protein Databank. The reference ligand used is doxorubicin.

### 4.3. *In vitro* anti-proliferative activity

The cytotoxicity assays were performed at Al-Azhar University, Pharmacology & Toxicology Department, Cairo, Egypt. Cancer cells from different cancer cell lines HCT-116, HepG2 and MCF-7, were purchased from ATCC, Manassas, USA and grown on the appropriate growth medium Roswell Park Memorial Institute medium (RPMI 1640) supplemented with 100 mg/mL of streptomycin, 100 units/mL of penicillin and 10% of heat-inactivated foetal bovine serum in a humidified, 5% (v/v) CO_2_ atmosphere at 37 °C Cytotoxicity assay by 3-[4,5-dimethylthiazole-2-yl]-2,5-diphenyltetrazolium bromide (MTT).

Cancer cell lines were trypsinized, counted and seeded into 96-well microtiter plates. Cells then were incubated for 24 h in a humidified atmosphere at 37 °C. Then exposed to different concentrations of derivatives (0.1, 10, 100 and 1000 µM) for 72 h. Then the viability of treated cells was determined using the MTT technique^46–48^.

### 4.4. *In vitro* DNA/methyl green assay

Methyl green dye can bind DNA to form coloured DNA/methyl green reversible complex. These complexes at neutral pH are still stable. The methyl green is displaced from DNA upon intercalating agents addition. Colourless carbinol was formed by the addition of H_2_O to the dye, leading to a dramatic decrease in spectrophotometric absorbance. ΔA value (the difference between DNA/methyl green complex and free cabinol) provides the simplest means for detecting the DNA-binding affinity and relative binding strength. IC_50_ values were determined using the GraphPadPrism 5.0 software^21^^,^^22^^,^^49^.

### 4.5. *In vitro* topoisomerase II inhibitory activity

A mixture of human Topo II (2 µl), substrate super coiled pHot1 DNA (0.25 µg), 50 µg/ml test compound (2 µl), and assay buffer (4 µl). The reaction started upon incubation of the mixture for 30 min at 37 °C. The reaction was terminated by the addition of proteinase K (50 µg/ml) and 10% sodium dodecylsulphate (2 µl) for 15 min at 37 °C. followed by incubation at 37 °C for 15 min. Then, the DNA was run for 1–2 h on 1% agarose gel in BioRad gel electrophoresis system followed by staining with GelRedTM stain for 2 h and destained for 15 min with TAE buffer. The gel was imaged *via* BioRad’s Gel DocTMEZ system. Both supercoiled and linear strands of DNA were incorporated into the gel as markers for DNA-Topo II intercalators. By using the GraphPad Prism version 5.0, the values of IC_50_ were calculated. Each reaction was performed in duplicate, and at least three independent determinations of each IC_50_ were made.

The data is available in a supplementary file.

## Supplementary Material

Supplemental Material
