# Supplementary material for: Triazoloquinoxalines-based DNA intercalators-Topo II inhibitors: design, synthesis, docking, ADMET and anti-proliferative evaluations
Source: J Enzyme Inhib Med Chem. 2022 May 29;37(1):1556–67. doi: 10.1080/14756366.2022.2080205 (PMC9154796; doi:10.1080/14756366.2022.2080205)
Supplement: Supplemental Material [file IENZ_A_2080205_SM1260.pdf]

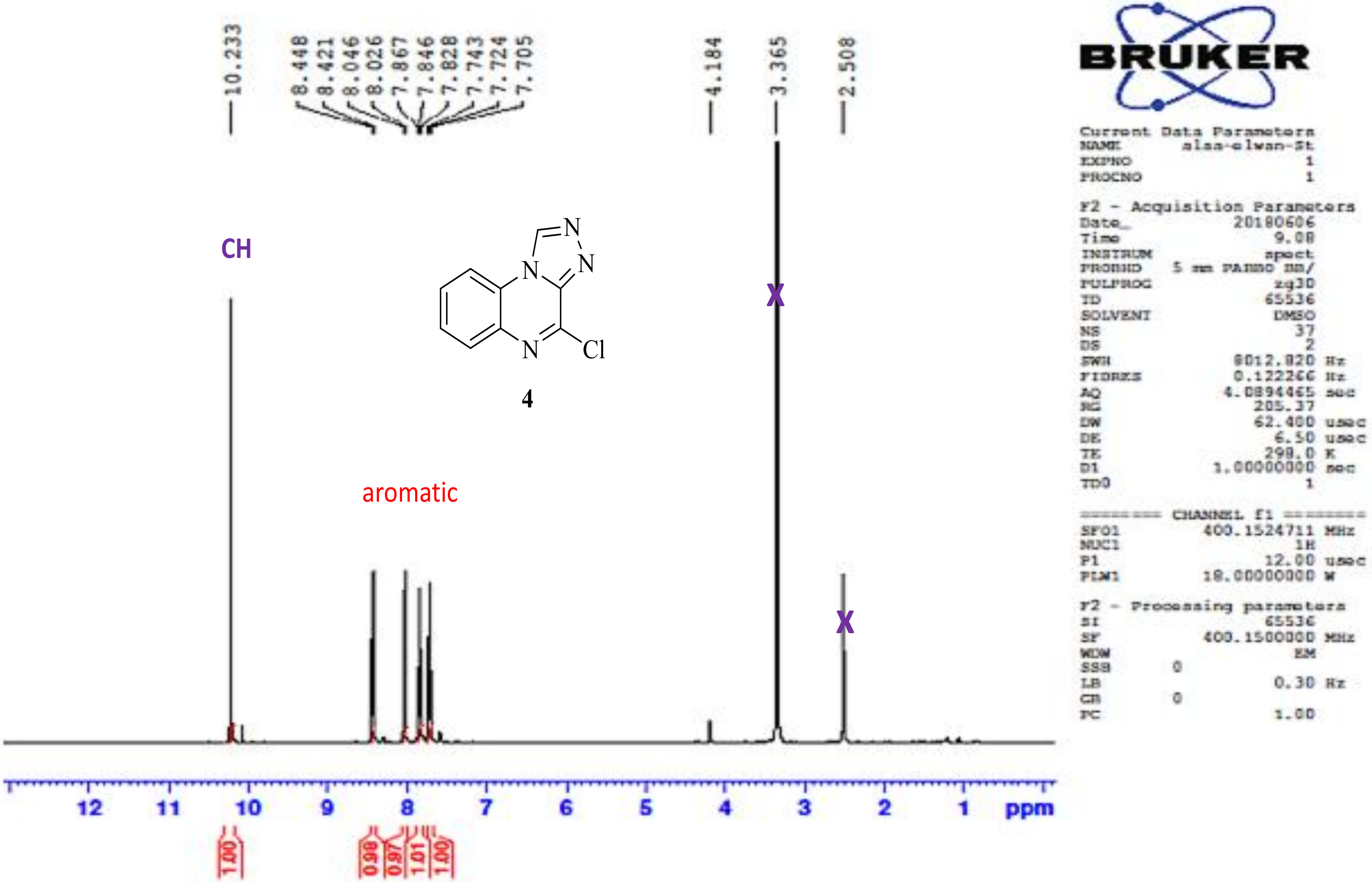

10.233
8.448
8.421
8.046
8.026
7.867
7.846
7.828
7.743
7.724
7.705
4.184
3.365
2.508
CH
aromatic
4
X
X
12 11 10 9 8 7 6 5 4 3 2 1 ppm
1.00
0.98
0.97
1.01
1.00
BRUKER
Current Data Parameters
NAME alaa-elwan-St
EXPNO 1
PROCNO 1
F2 - Acquisition Parameters
Date_ 20180606
Time 9.08
INSTRUM spect
PROBHD 5 mm PABBO BB/
PULPROG zg30
TD 65536
SOLVENT DMSO
NS 37
DS 2
SWH 8012.820 Hz
FIDRES 0.122266 Hz
AQ 4.0894465 sec
RG 205.37
DW 62.400 usec
DE 6.50 usec
TE 298.0 K
D1 1.00000000 sec
TD0 1
======== CHANNEL f1 ========
SFO1 400.1524711 MHz
NUC1 1H
P1 12.00 usec
PLW1 18.00000000 W
F2 - Processing parameters
SI 65536
SF 400.1500000 MHz
WDW EM
SSB 0
LB 0.30 Hz
GB 0
PC 1.00

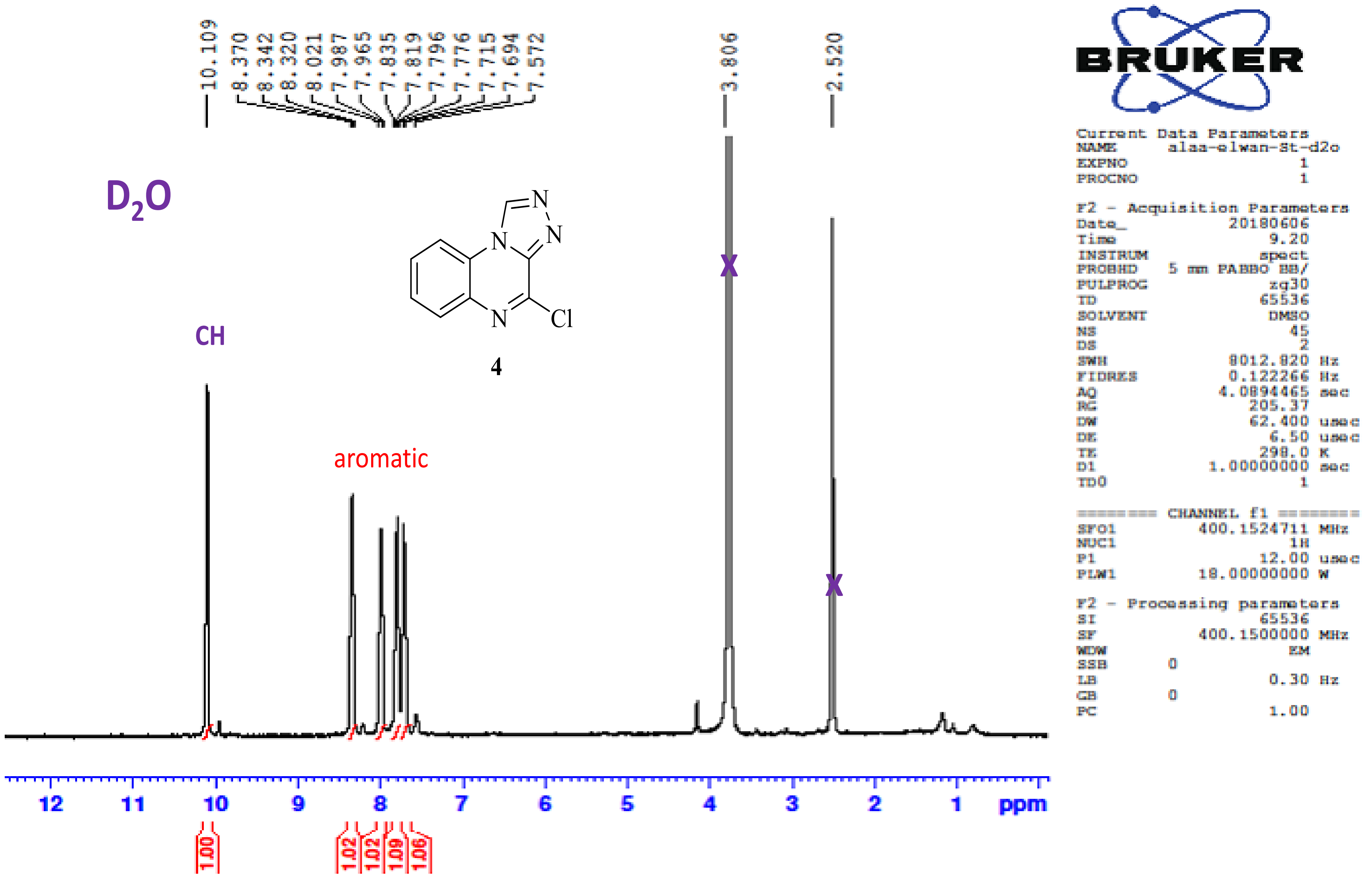
10.109
8.370
8.342
8.320
8.021
7.987
7.965
7.835
7.819
7.796
7.776
7.715
7.694
7.572
3.806
2.520
D2O
CH
aromatic
N
N
N
N
N
Cl
4
X
X
12 11 10 9 8 7 6 5 4 3 2 1 ppm
1.00
1.02
1.02
1.09
1.06
BRUKER
Current Data Parameters
NAME alaa-elwan-St-d2o
EXPNO 1
PROCNO 1
F2 - Acquisition Parameters
Date_ 20180606
Time 9.20
INSTRUM spect
PROBHD 5 mm PABBO BB/
PULPROG zg30
TD 65536
SOLVENT DMSO
NS 45
DS 2
SWH 8012.820 Hz
FIDRES 0.122266 Hz
AQ 4.0894465 sec
RG 205.37
DW 62.400 usec
DE 6.50 usec
TE 298.0 K
D1 1.00000000 sec
TD0 1
======== CHANNEL f1 ========
SFO1 400.1524711 MHz
NUC1 1H
P1 12.00 usec
PLW1 18.00000000 W
F2 - Processing parameters
SI 65536
SF 400.1500000 MHz
WDW EM
SSB 0
LB 0.30 Hz
GB 0
PC 1.00

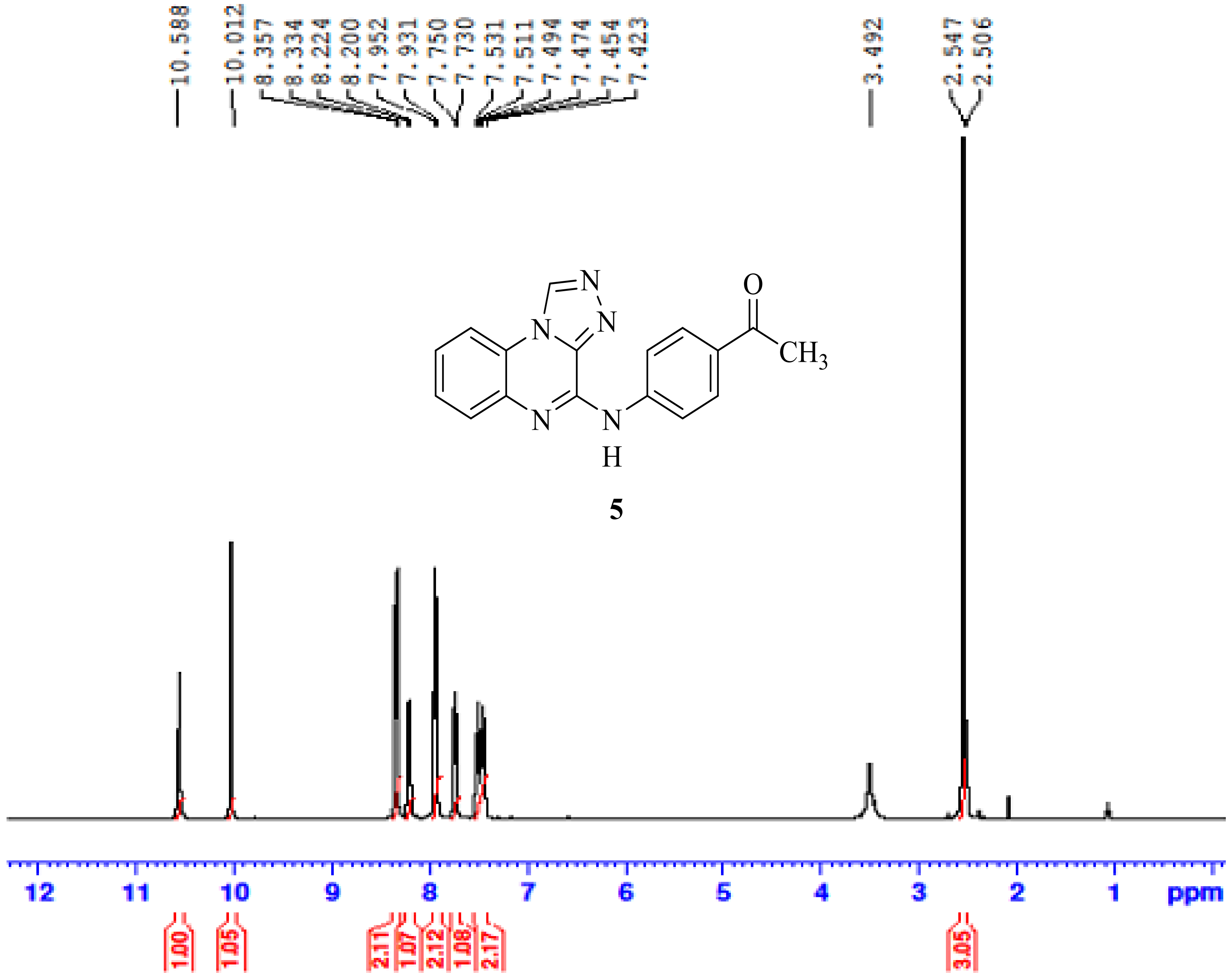

Current Data Parameters

NAME	alaa-P-A-AC
EXPNO	1
PROCNO	1

F2 - Acquisition Parameters

Date_	20180225
Time	9.49
INSTRUM	spect
PROBHD	5 mm PABBO BB/
PULPROG	zg30
TD	65536
SOLVENT	DMSO
NS	45
DS	2
SWH	8012.820 Hz
FIDRES	0.122266 Hz
AQ	4.0894465 sec
RG	205.37
DW	62.400 usec
DE	6.50 usec
TE	298.0 K
D1	1.00000000 sec
TD0	1

======== CHANNEL f1 ========

SFO1	400.1524711 MHz
NUC1	1H
P1	12.00 usec
PLW1	18.00000000 W

F2 - Processing parameters

SI	65536
SF	400.1500000 MHz
WDW	EM
SSB	0
LB	0.30 Hz
GB	0
PC	1.00

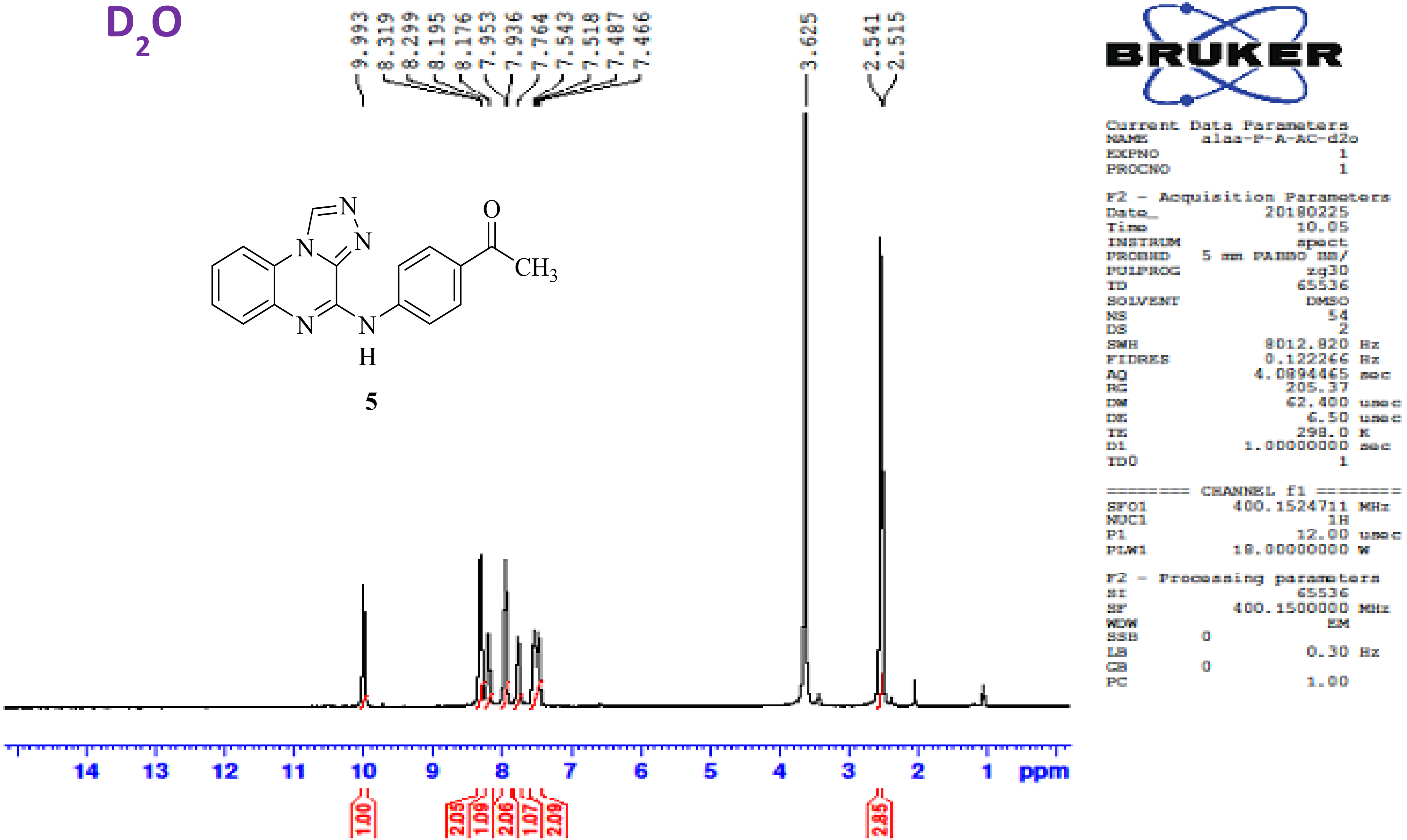

D2O
9.993
8.319
8.299
8.195
8.176
7.953
7.936
7.764
7.543
7.518
7.487
7.466
3.625
2.541
2.515
O
N
N
N
N
N
H
CH3
5
Current Data Parameters
NAME alaa-P-A-AC-d2o
EXPNO 1
PROCNO 1
F2 - Acquisition Parameters
Date_ 20180225
Time 10.05
INSTRUM spect
PROBHD 5 mm PABBO BB/
PULPROG zg30
TD 65536
SOLVENT DMSO
NS 54
DS 2
SWH 8012.820 Hz
FIDRES 0.122266 Hz
AQ 4.0894465 sec
RG 205.37
DW 62.400 usec
DE 6.50 usec
TE 298.0 K
D1 1.00000000 sec
TD0 1
======== CHANNEL f1 ========
SFO1 400.1524711 MHz
NUC1 1H
P1 12.00 usec
PLW1 18.00000000 W
F2 - Processing parameters
SI 65536
SF 400.1500000 MHz
WDW EM
SSB 0
LB 0.30 Hz
GB 0
PC 1.00
14 13 12 11 10 9 8 7 6 5 4 3 2 1 ppm
1.00
2.05
1.09
2.06
1.07
2.09
2.85

Alaa ElWan_C_P-AAC

Microanalytical Unit - FOPCU - NMR laboratory
www.pharma.cu.edu.eg dir-mau.fopcu@pharma.cu.edu.eg

BRUKER

```
Current Data Parameters
NAME        Alaa ElWan_C_P-AAC
EXPNO                     10
PROCNO                     1

F2 - Acquisition Parameters
Date_             20180726
Time                 20.42
INSTRUM              spect
PROBHD     5 mm PABBO BB/
PULPROG            zgpg30
TD                  65536
SOLVENT              DMSO
NS                   1200
DS                      4
SWH            24038.461 Hz
FIDRES          0.366798 Hz
AQ             1.3631488 sec
RG                 202.37
DW                 20.800 usec
DE                   6.50 usec
TE                  298.0 K
D1            2.00000000 sec
D11           0.03000000 sec
TD0                     1

======== CHANNEL f1 ========
SFO1         100.6379178 MHz
NUC1                 13C
P1                 10.00 usec
PLW1         45.00000000 W

======== CHANNEL f2 ========
SFO2         400.1916008 MHz
NUC2                  1H
CPDPRG[2          waltz16
```

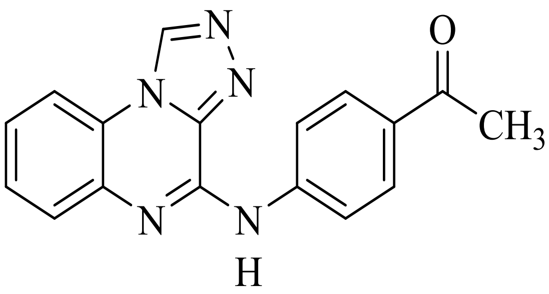

5

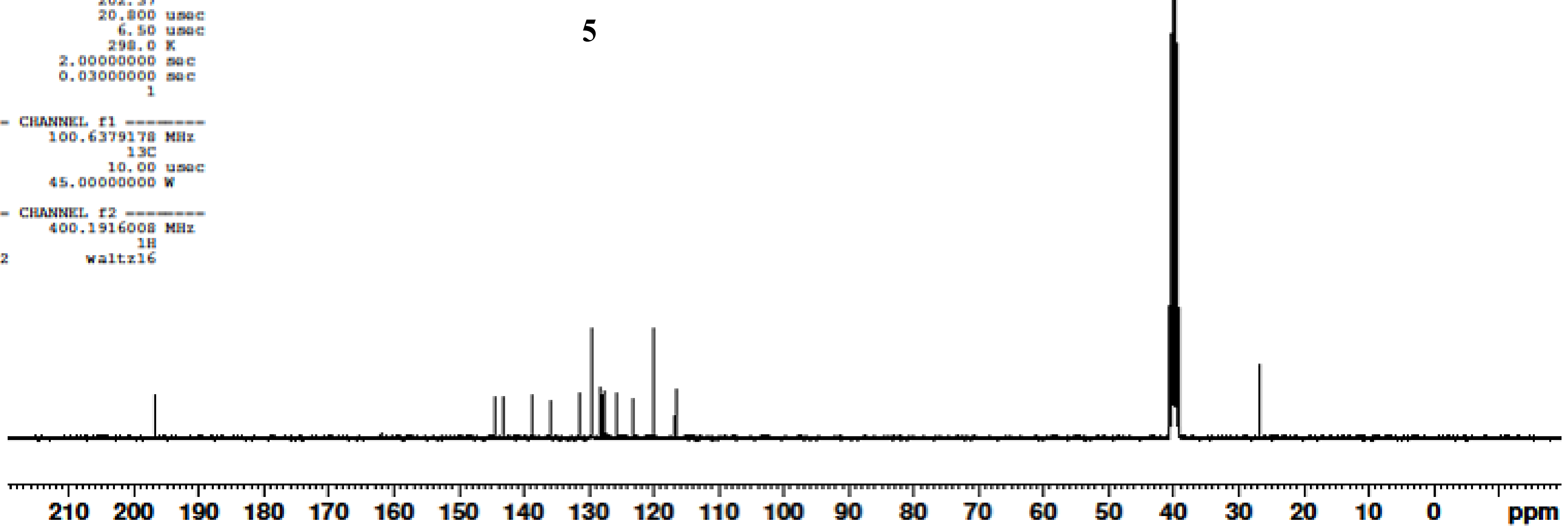

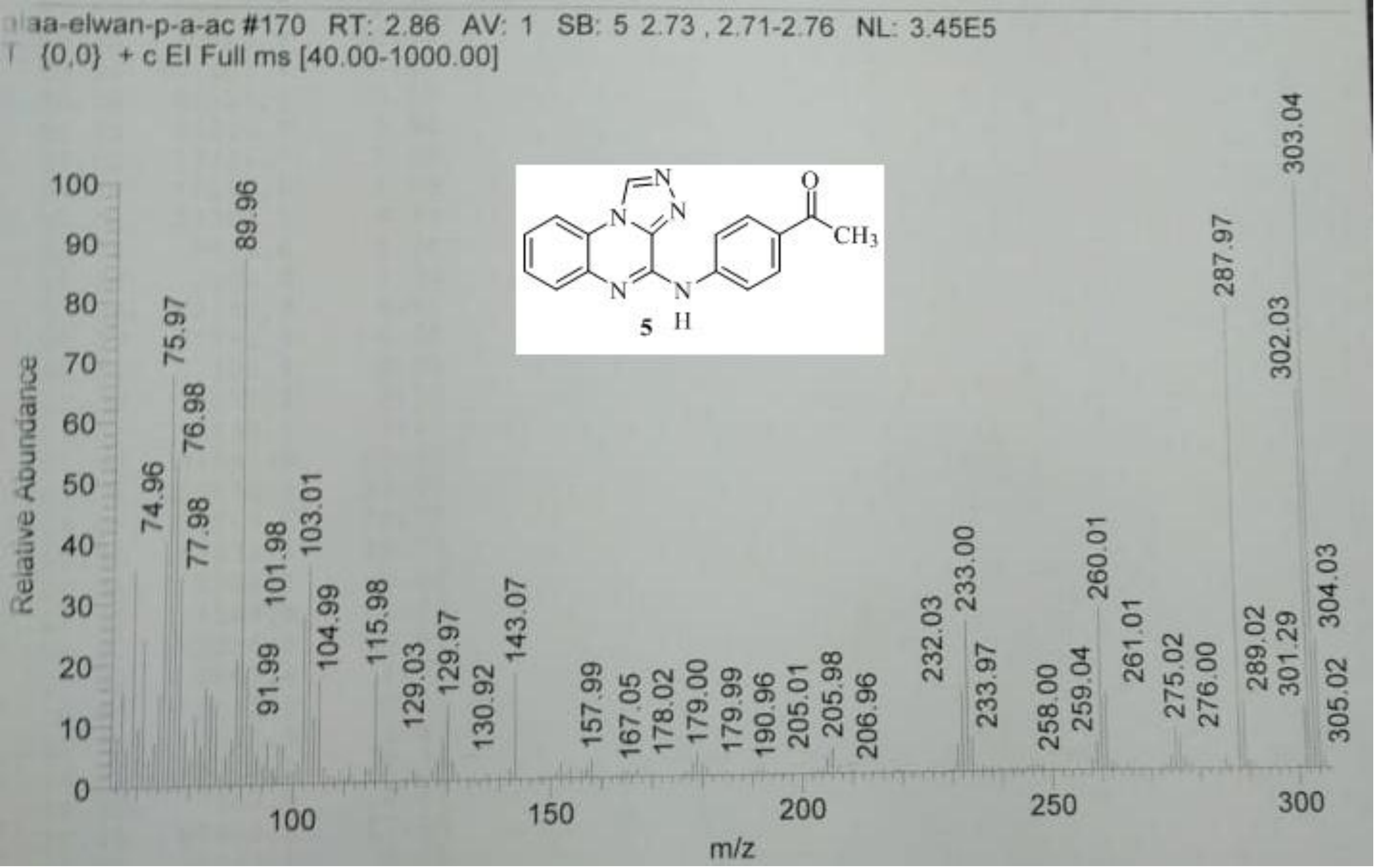
aa-elwan-p-a-ac #170 RT: 2.86 AV: 1 SB: 5 2.73 , 2.71-2.76 NL: 3.45E5
{0,0} + c EI Full ms [40.00-1000.00]
Relative Abundance
m/z
0
10
20
30
40
50
60
70
80
90
100
150
200
250
300
5
N
H
O
CH3
74.96
75.97
76.98
77.98
89.96
91.99
101.98
103.01
104.99
115.98
129.03
129.97
130.92
143.07
157.99
167.05
178.02
179.00
179.99
190.96
205.01
205.98
206.96
232.03
233.00
233.97
258.00
259.04
260.01
261.01
275.02
276.00
287.97
289.02
301.29
302.03
303.04
304.03
305.02

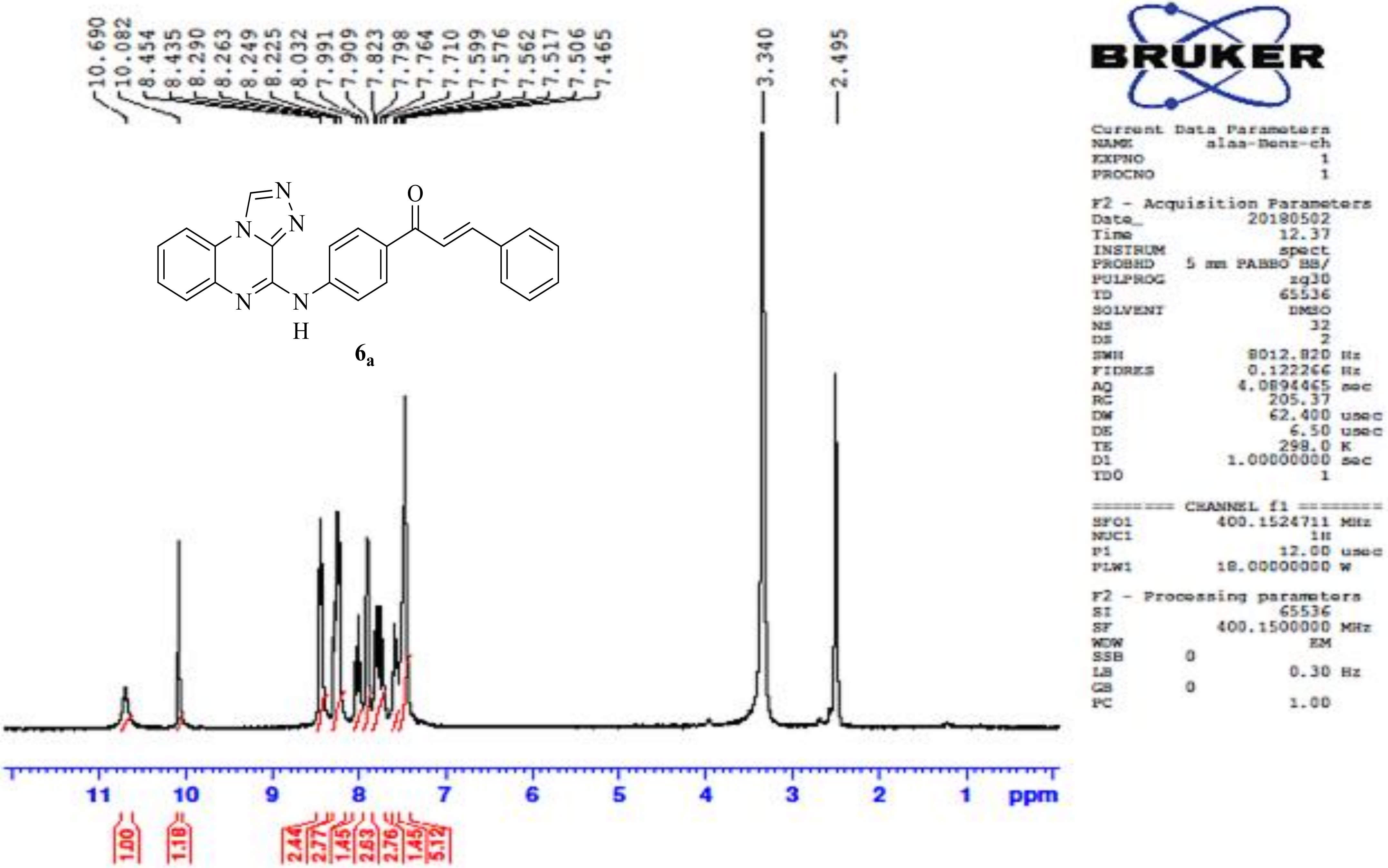
10.690
10.082
8.454
8.435
8.290
8.263
8.249
8.225
8.032
7.991
7.909
7.823
7.798
7.764
7.710
7.599
7.576
7.562
7.517
7.506
7.465
3.340
2.495
N
N
N
N
O
N
H
6a
BRUKER
Current Data Parameters
NAME alaa-Benz-ch
EXPNO 1
PROCNO 1
F2 - Acquisition Parameters
Date_ 20180502
Time 12.37
INSTRUM spect
PROBHD 5 mm PABBO BB/
PULPROG zg30
TD 65536
SOLVENT DMSO
NS 32
DS 2
SWH 8012.820 Hz
FIDRES 0.122266 Hz
AQ 4.0894465 sec
RG 205.37
DW 62.400 usec
DE 6.50 usec
TE 298.0 K
D1 1.00000000 sec
TD0 1
======= CHANNEL f1 =======
SFO1 400.1524711 MHz
NUC1 1H
P1 12.00 usec
PLW1 18.00000000 W
F2 - Processing parameters
SI 65536
SF 400.1500000 MHz
WDW EM
SSB 0
LB 0.30 Hz
GB 0
PC 1.00
11
10
9
8
7
6
5
4
3
2
1
ppm
1.00
1.18
2.44
2.77
1.45
2.63
2.76
1.45
5.12

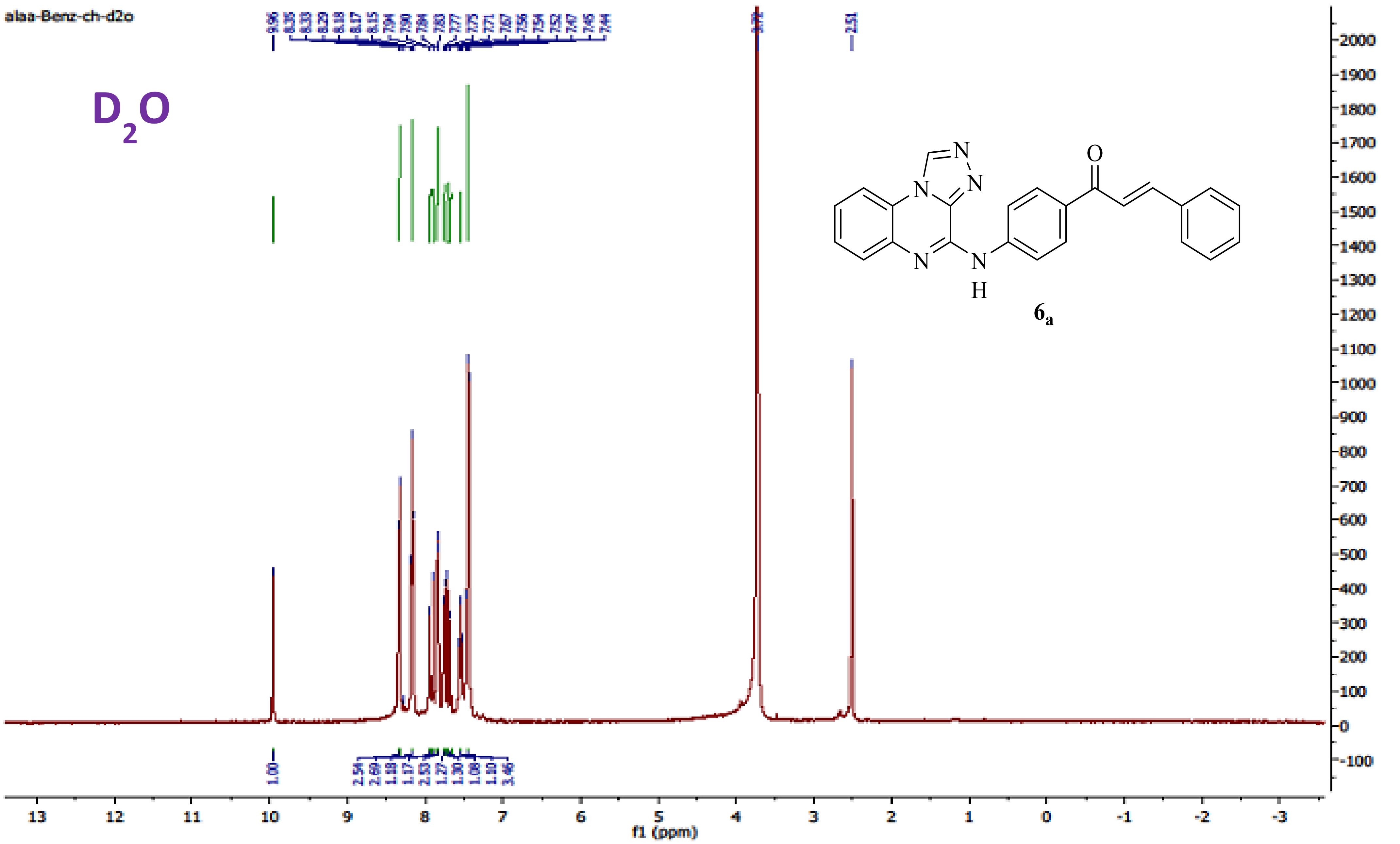
alaa-Benz-ch-d2o
D2O
6a
f1 (ppm)

Alaa ElWan_C_Benz-Ch

Microanalytical Unit - FOPCU - NMR laboratory
www.pharma.cu.edu.eg dir-mau.fopcu@pharma.cu.edu.eg

BRUKER

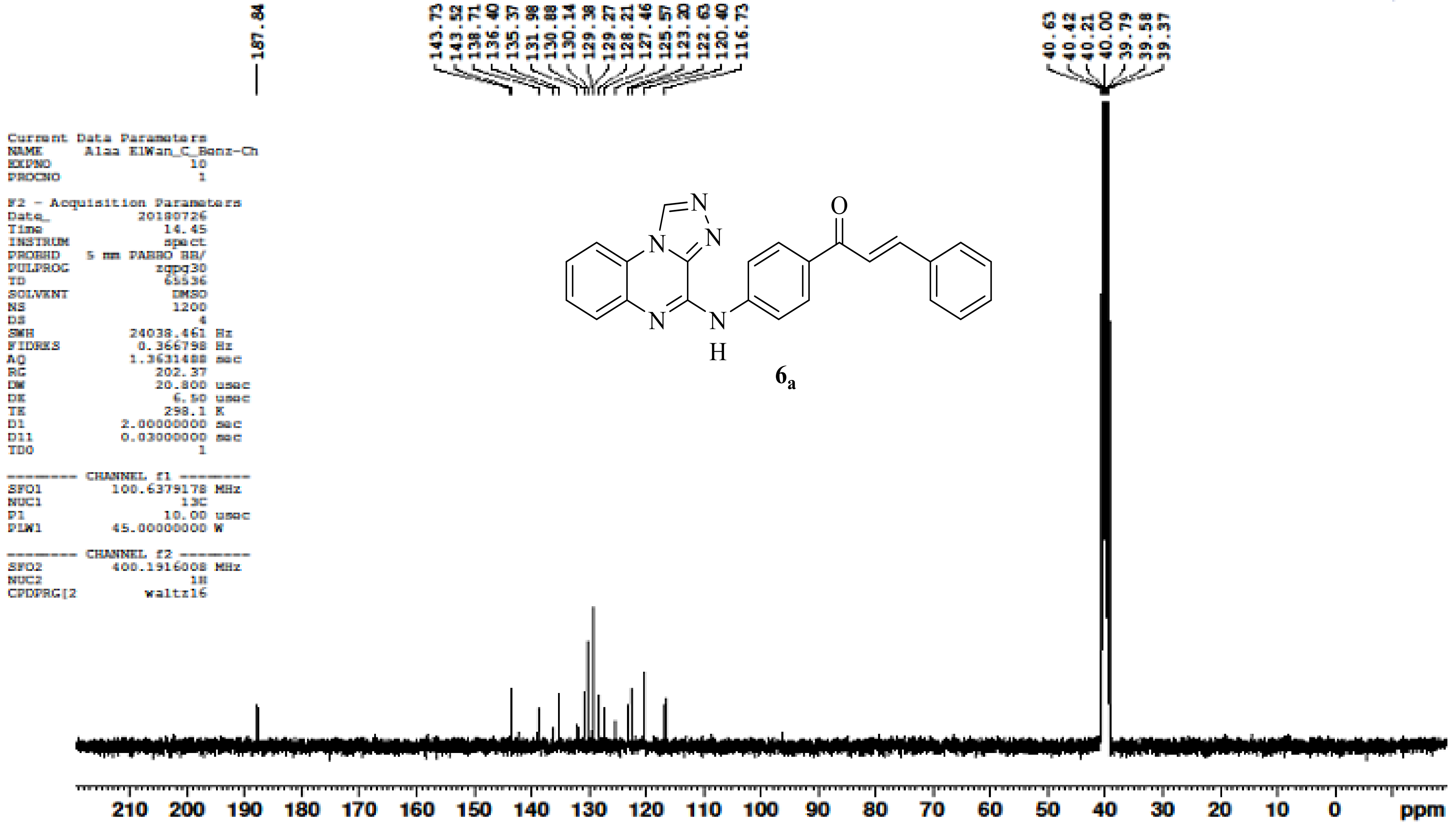

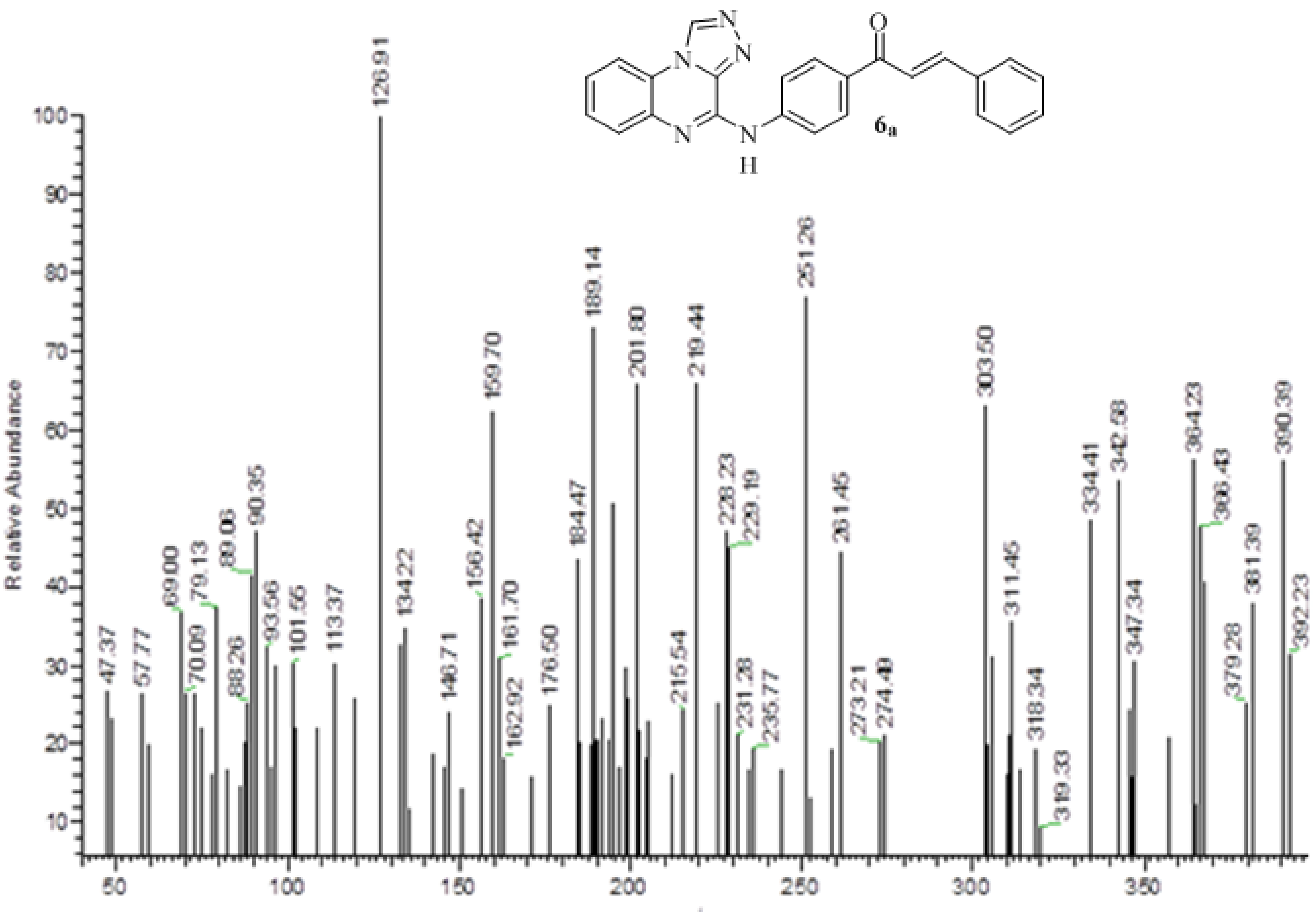
6a
Relative Abundance
126.91
251.26
189.14
201.80
219.44
303.50
159.70
364.23
390.39
342.58
334.41
90.35
228.23
261.45
311.45
347.34
381.30
392.23
379.28
318.34
319.33
274.49
273.21
235.77
231.28
215.54
184.47
176.50
162.92
161.70
156.42
146.71
134.22
113.37
101.56
93.56
88.26
79.13
70.00
69.00
57.77
47.37

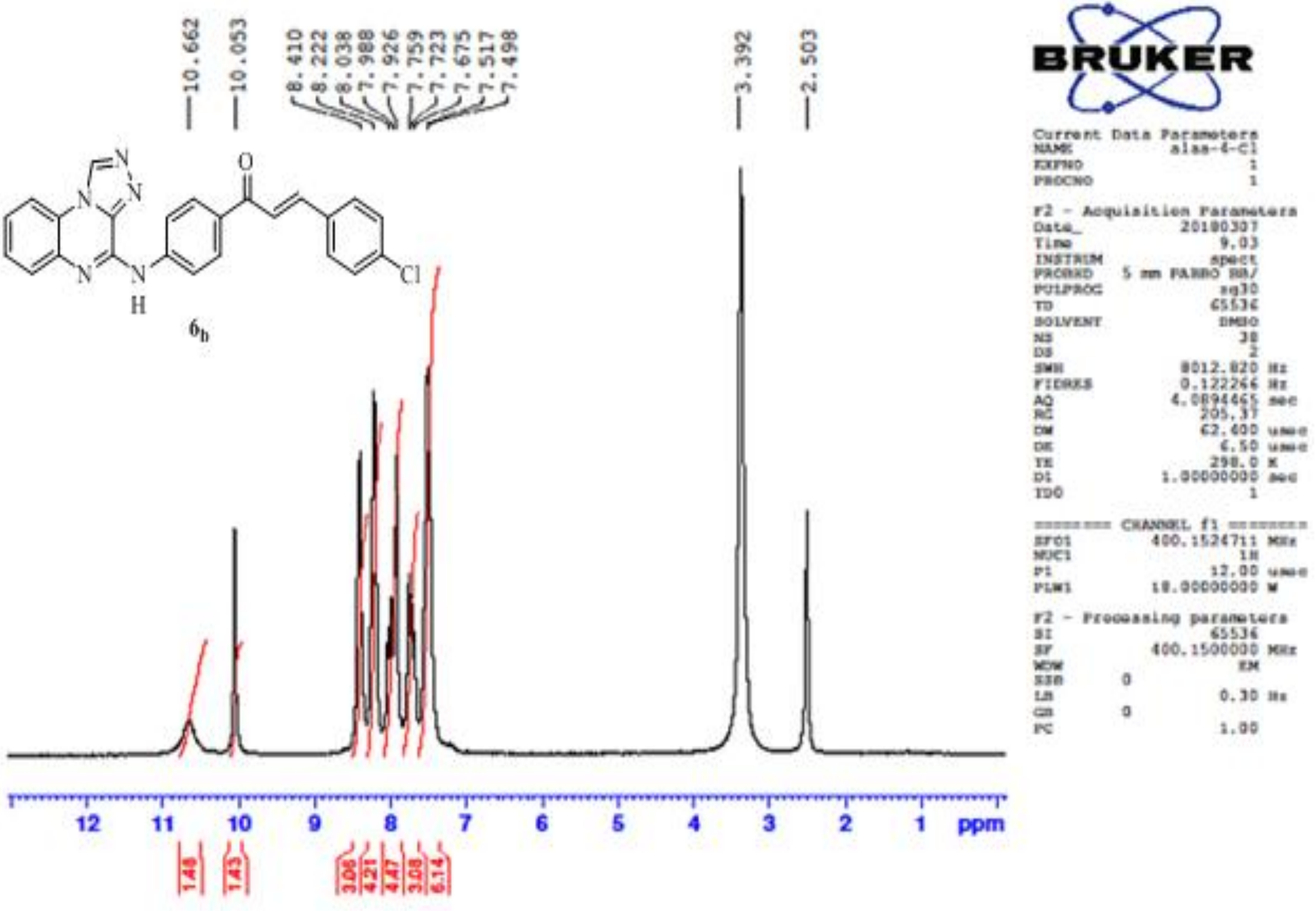
10.662
10.053
8.410
8.222
8.038
7.988
7.926
7.759
7.723
7.675
7.517
7.498
3.392
2.503
O
N
N
N
N
H
Cl
6b
BRUKER
Current Data Parameters
NAME alaa-4-Cl
EXPNO 1
PROCNO 1
F2 - Acquisition Parameters
Date_ 20180307
Time 9.03
INSTRUM spect
PROBHD 5 mm PABBO BB/
PULPROG zg30
TD 65536
SOLVENT DMSO
NS 38
DS 2
SWH 8012.820 Hz
FIDRES 0.122266 Hz
AQ 4.0894465 sec
RG 205.37
DW 62.400 usec
DE 6.50 usec
TE 298.0 K
D1 1.00000000 sec
TD0 1
======== CHANNEL f1 ========
SFO1 400.1524711 MHz
NUC1 1H
P1 12.00 usec
PLW1 18.00000000 W
F2 - Processing parameters
SI 65536
SF 400.1500000 MHz
WDW EM
SSB 0
LB 0.30 Hz
GB 0
PC 1.00
12
11
10
9
8
7
6
5
4
3
2
1
ppm
1.48
1.43
3.08
4.21
4.47
3.08
6.14

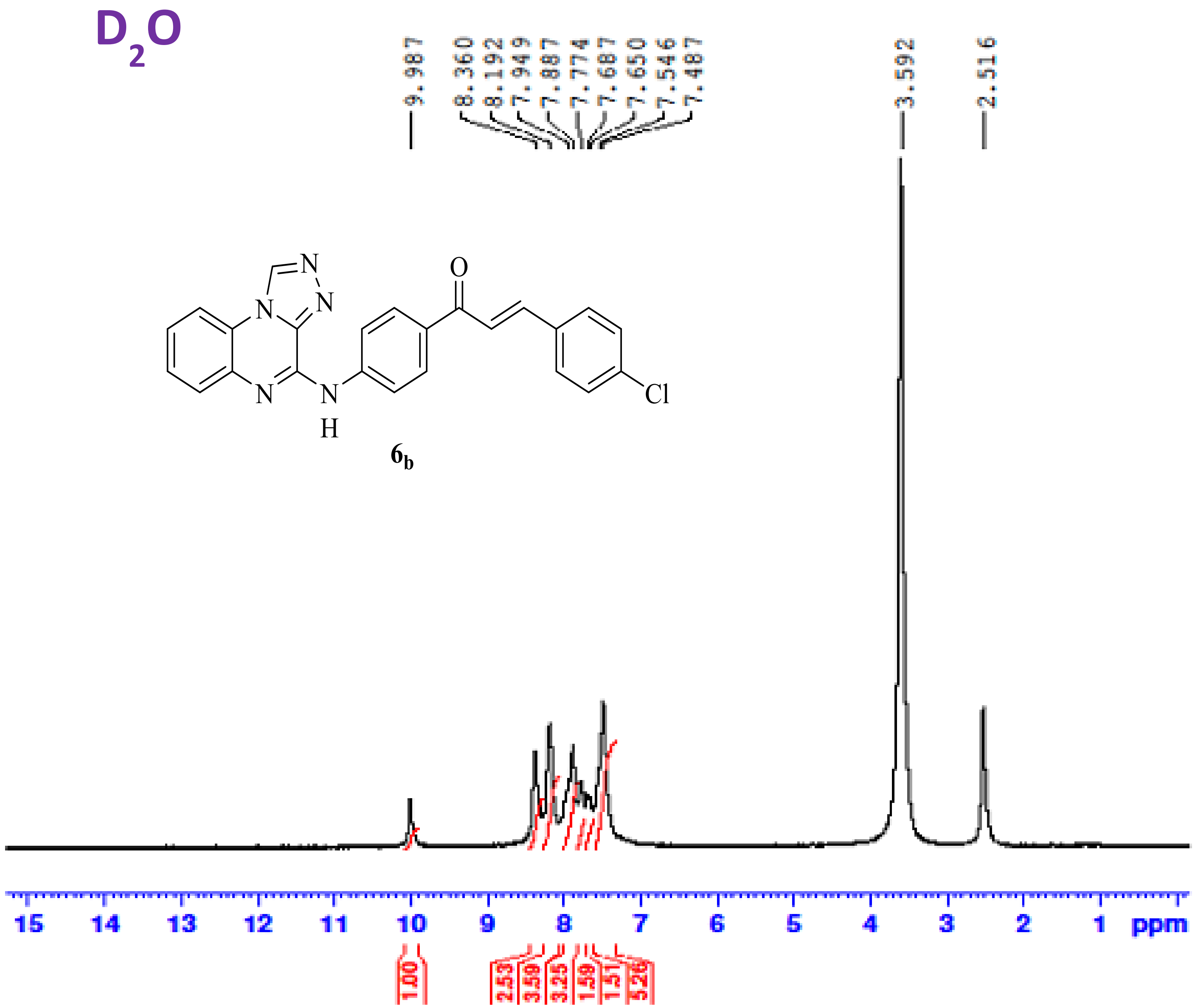

BRUKER

Current Data Parameters
NAME alaa-4-Cl-d2o
EXPNO 1
PROCNO 1

F2 - Acquisition Parameters
Date_ 20180307
Time 9.17
INSTRUM spect
PROBHD 5 mm PABBO BB/
PULPROG zg30
TD 65536
SOLVENT DMSO
NS 37
DS 2
SWH 8012.820 Hz
FIDRES 0.122266 Hz
AQ 4.0894465 sec
RG 205.37
DW 62.400 usec
DE 6.50 usec
TE 298.9 K
D1 1.00000000 sec
TD0 1

======== CHANNEL f1 ========
SFO1 400.1524711 MHz
NUC1 1H
P1 12.00 usec
PLW1 18.00000000 W

F2 - Processing parameters
SI 65536
SF 400.1500000 MHz
WDW EM
SSB 0
LB 0.30 Hz
GB 0
PC 1.00

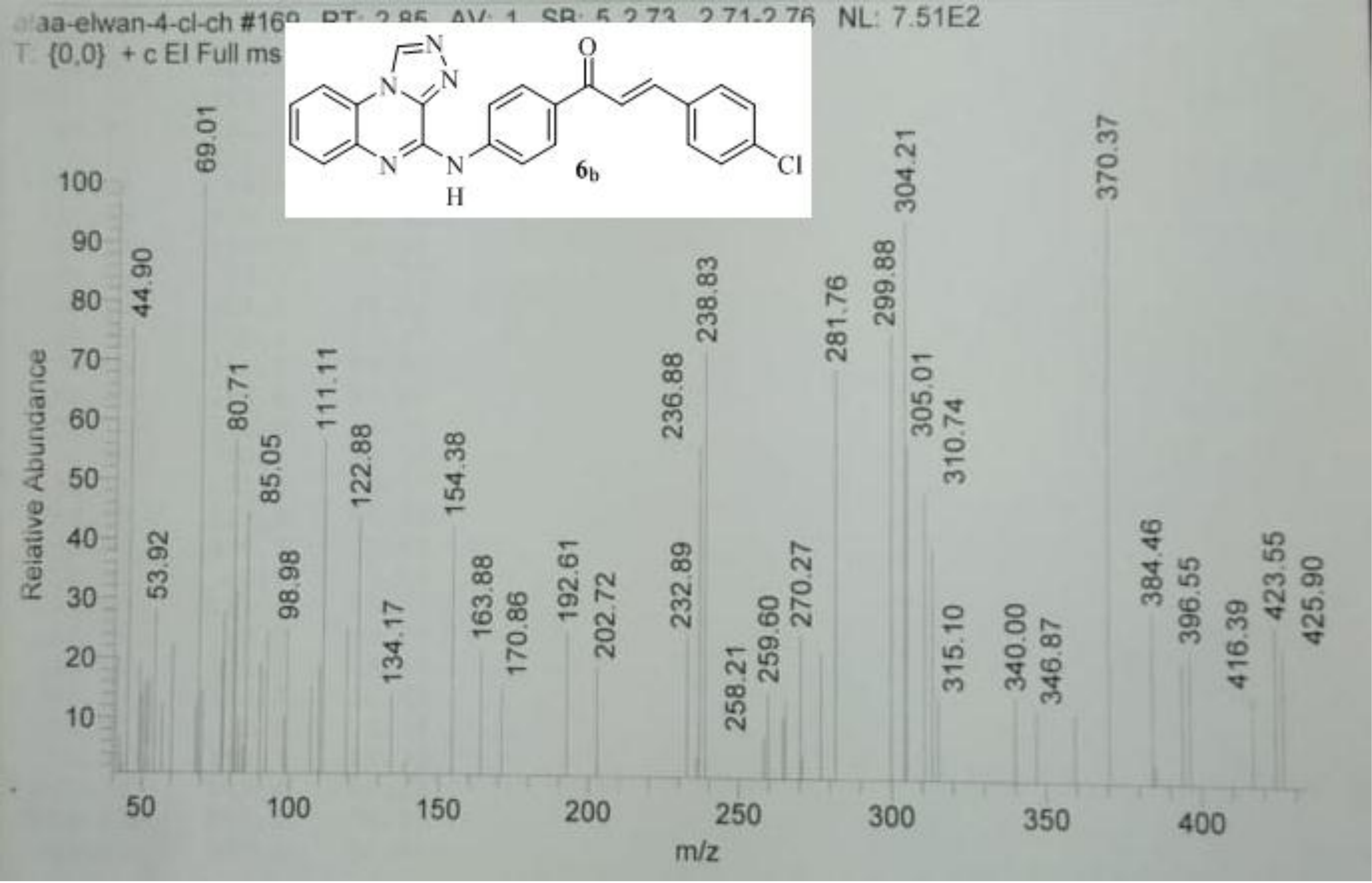

aa-elwan-4-cl-ch
NL: 7.51E2
T: {0,0} + c EI Full ms
6b
Cl
Relative Abundance
m/z
69.01
44.90
53.92
80.71
85.05
98.98
111.11
122.88
134.17
154.38
163.88
170.86
192.61
202.72
232.89
236.88
238.83
258.21
259.60
270.27
281.76
299.88
304.21
305.01
310.74
315.10
340.00
346.87
370.37
384.46
396.55
416.39
423.55
425.90

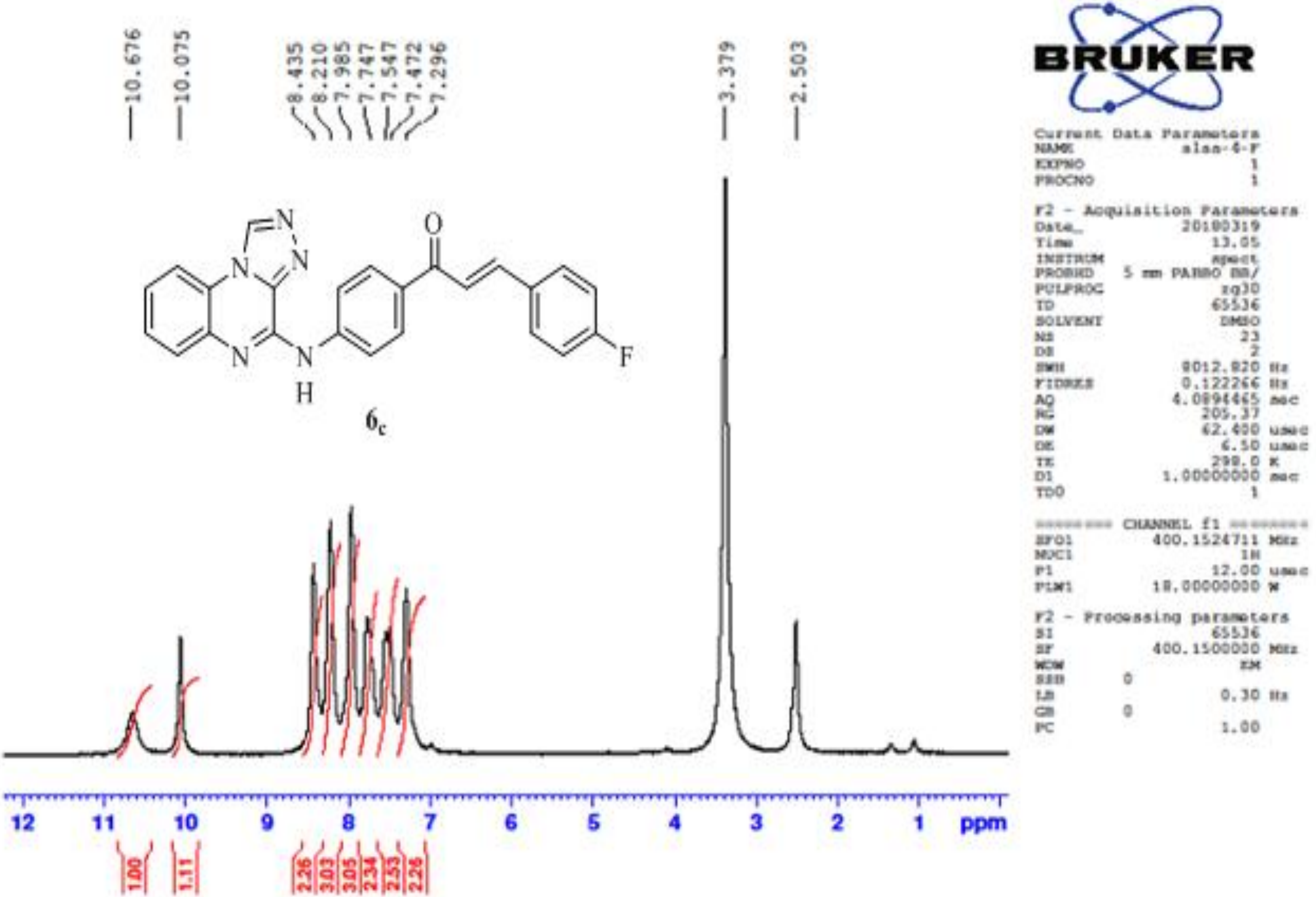

10.676
10.075
8.435
8.210
7.985
7.747
7.547
7.472
7.296
3.379
2.503
6c
12 11 10 9 8 7 6 5 4 3 2 1 ppm
1.00 1.11 2.26 3.03 3.05 2.34 2.53 2.26
BRUKER
Current Data Parameters
NAME alaa-4-F
EXPNO 1
PROCNO 1
F2 - Acquisition Parameters
Date_ 20180319
Time 13.05
INSTRUM spect
PROBHD 5 mm PABBO BB/
PULPROG zg30
TD 65536
SOLVENT DMSO
NS 23
DS 2
SWH 8012.820 Hz
FIDRES 0.122266 Hz
AQ 4.0894465 sec
RG 205.37
DW 62.400 usec
DE 6.50 usec
TE 298.0 K
D1 1.00000000 sec
TD0 1
======== CHANNEL f1 ========
SFO1 400.1524711 MHz
NUC1 1H
P1 12.00 usec
PLW1 18.00000000 W
F2 - Processing parameters
SI 65536
SF 400.1500000 MHz
WDW EM
SSB 0
LB 0.30 Hz
GB 0
PC 1.00

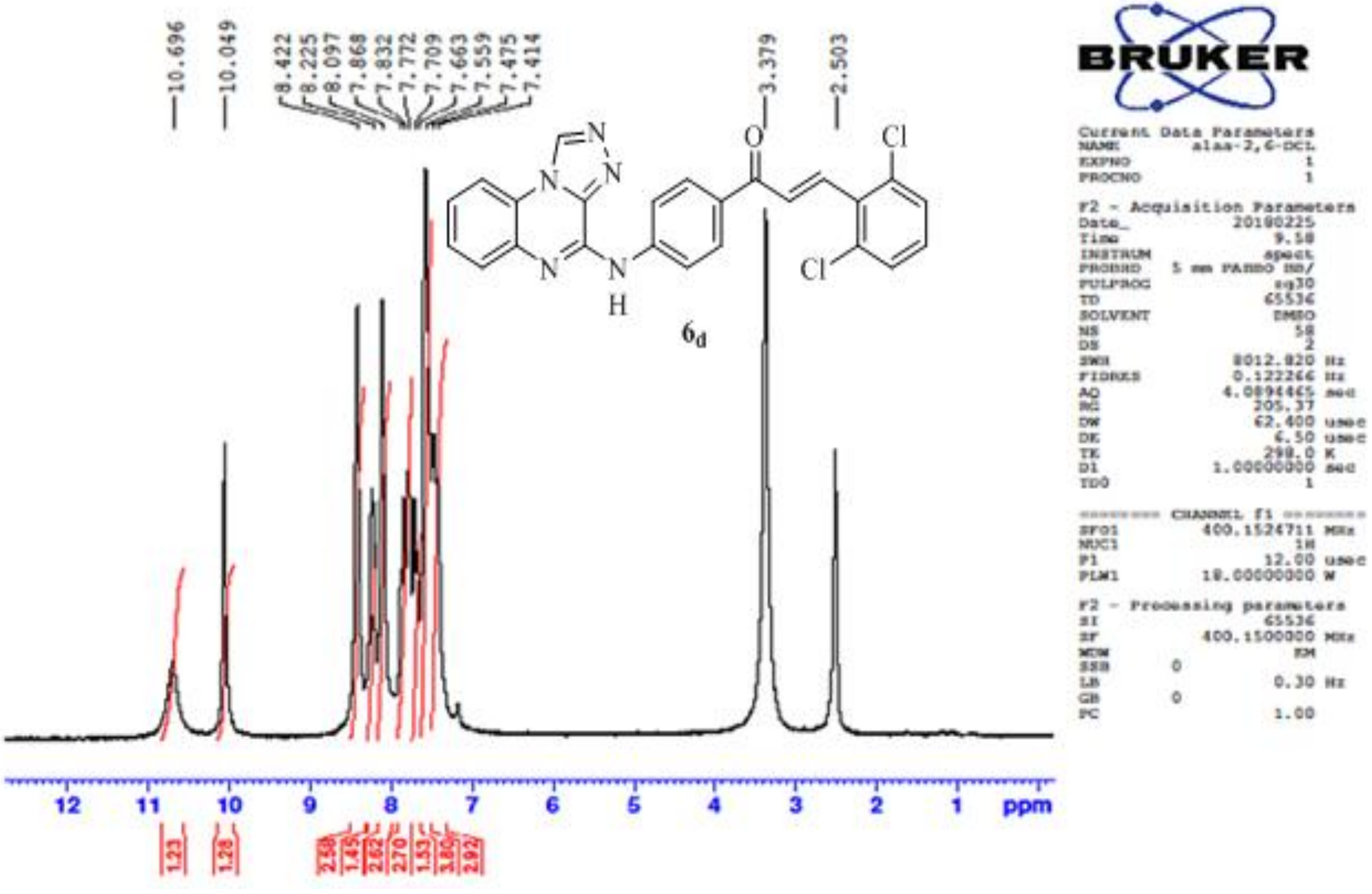
10.696
10.049
8.422
8.225
8.097
7.868
7.832
7.772
7.709
7.663
7.559
7.475
7.414
3.379
2.503
6_d
ppm
1.21
1.28
2.38
1.45
2.62
2.70
1.53
3.80
2.92
Current Data Parameters
NAME alaa-2,6-DCL
EXPNO 1
PROCNO 1
F2 - Acquisition Parameters
Date_ 20180225
Time 9.58
INSTRUM spect
PROBHD 5 mm PABBO BB/
PULPROG zg30
TD 65536
SOLVENT DMSO
NS 58
DS 2
SWH 8012.820 Hz
FIDRES 0.122266 Hz
AQ 4.0894465 sec
RG 205.37
DW 62.400 usec
DE 6.50 usec
TE 298.0 K
D1 1.00000000 sec
TD0 1
======== CHANNEL f1 ========
SFO1 400.1524711 MHz
NUC1 1H
P1 12.00 usec
PLW1 18.00000000 W
F2 - Processing parameters
SI 65536
SF 400.1500000 MHz
WDW EM
SSB 0
LB 0.30 Hz
GB 0
PC 1.00

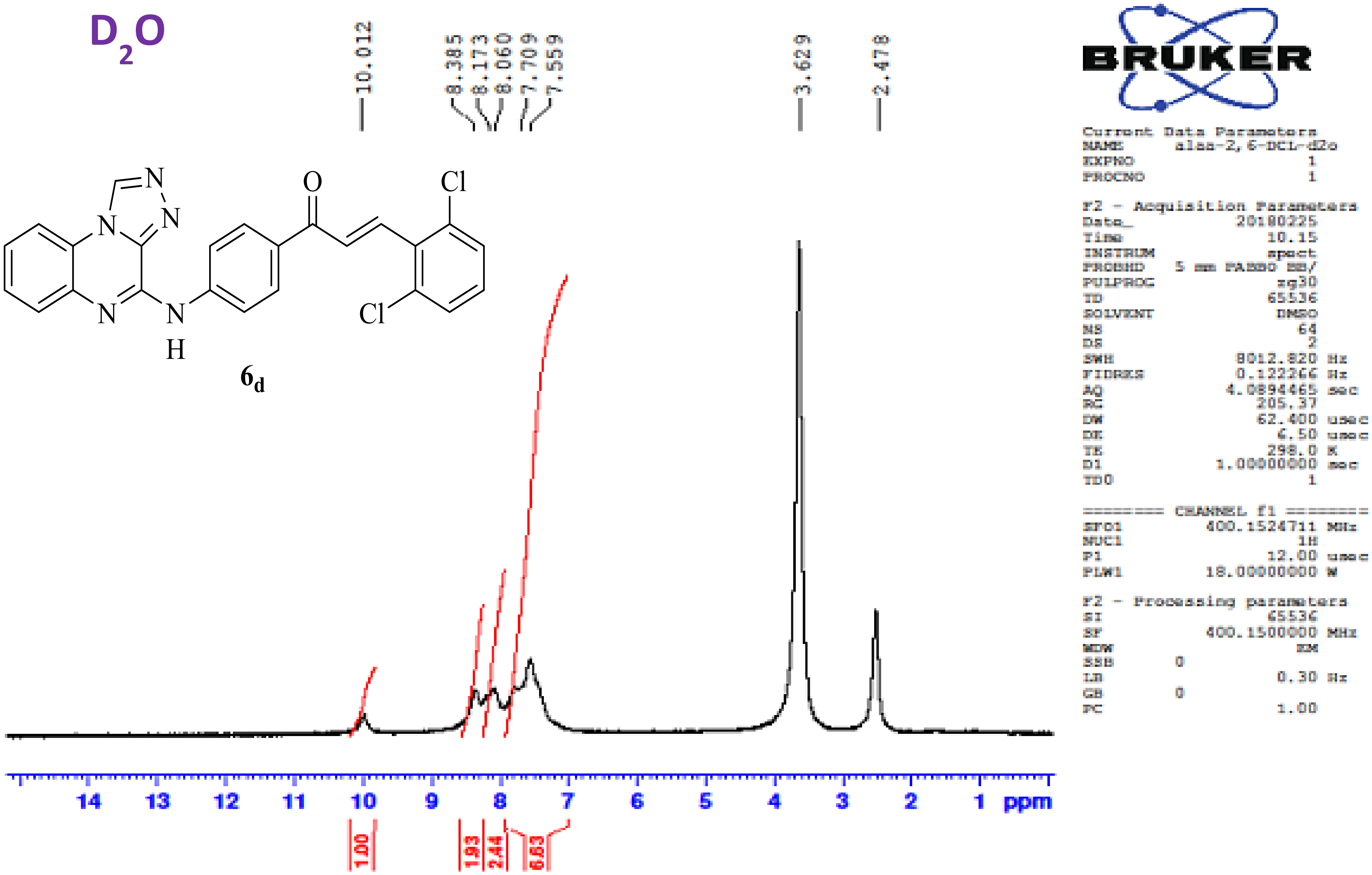
D2O
10.012
8.385
8.173
8.060
7.709
7.559
3.629
2.478
6d
Current Data Parameters
NAME alaa-2,6-DCL-d2o
EXPNO 1
PROCNO 1
F2 - Acquisition Parameters
Date_ 20180225
Time 10.15
INSTRUM spect
PROBHD 5 mm PABBO BB/
PULPROG zg30
TD 65536
SOLVENT DMSO
NS 64
DS 2
SWH 8012.820 Hz
FIDRES 0.122266 Hz
AQ 4.0894465 sec
RG 205.37
DW 62.400 usec
DE 6.50 usec
TE 298.0 K
D1 1.00000000 sec
TD0 1
======== CHANNEL f1 ========
SFO1 400.1524711 MHz
NUC1 1H
P1 12.00 usec
PLW1 18.00000000 W
F2 - Processing parameters
SI 65536
SF 400.1500000 MHz
WDW EM
SSB 0
LB 0.30 Hz
GB 0
PC 1.00
14 13 12 11 10 9 8 7 6 5 4 3 2 1 ppm
1.00
1.93
2.44
6.83

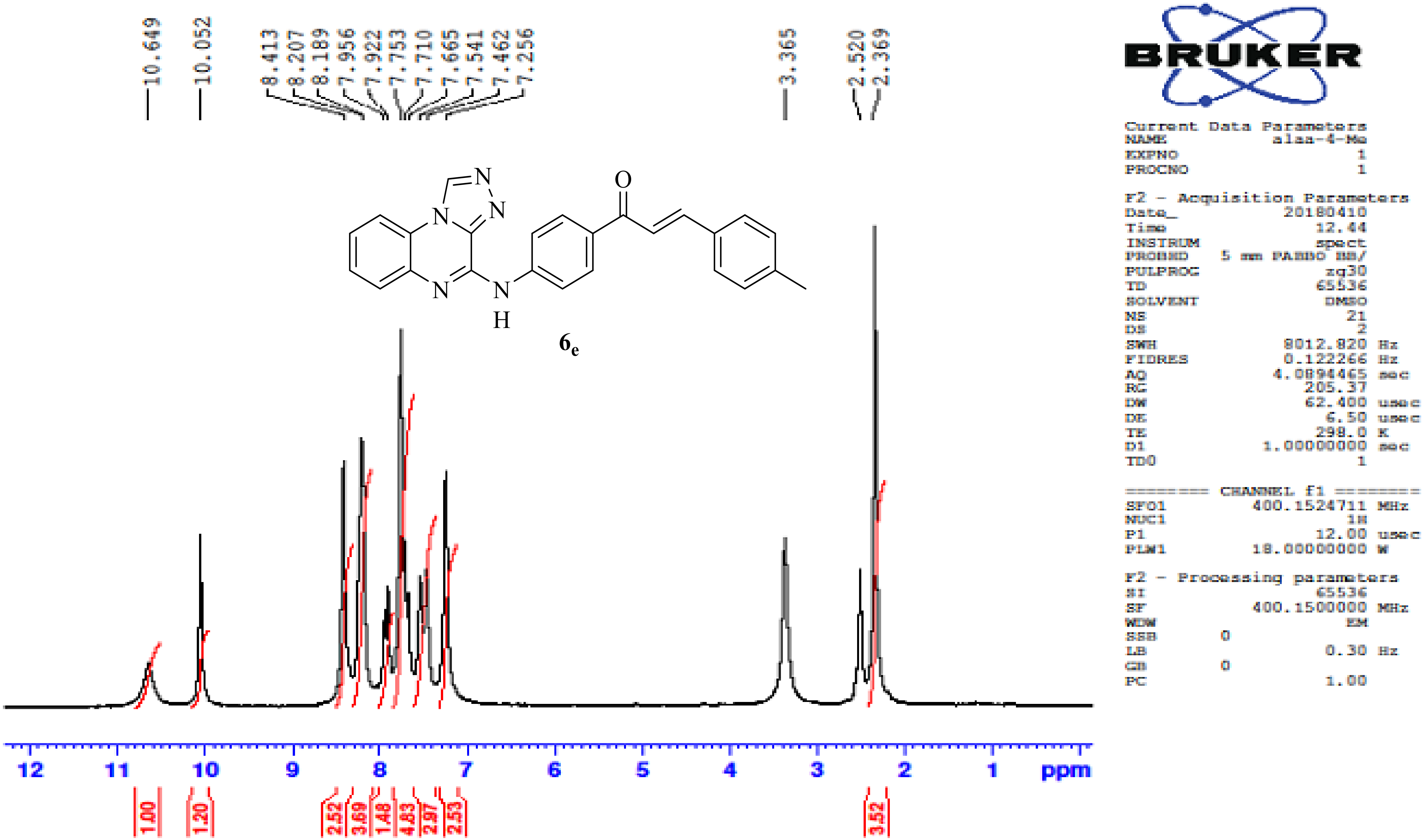
10.649
10.052
8.413
8.207
8.189
7.956
7.922
7.753
7.710
7.665
7.541
7.462
7.256
3.365
2.520
2.369
BRUKER
Current Data Parameters
NAME alaa-4-Me
EXPNO 1
PROCNO 1
F2 - Acquisition Parameters
Date_ 20180410
Time 12.44
INSTRUM spect
PROBHD 5 mm PABBO BB/
PULPROG zg30
TD 65536
SOLVENT DMSO
NS 21
DS 2
SWH 8012.820 Hz
FIDRES 0.122266 Hz
AQ 4.0894465 sec
RG 205.37
DW 62.400 usec
DE 6.50 usec
TE 298.0 K
D1 1.00000000 sec
TD0 1
======== CHANNEL f1 ========
SFO1 400.1524711 MHz
NUC1 1H
P1 12.00 usec
PLW1 18.00000000 W
F2 - Processing parameters
SI 65536
SF 400.1500000 MHz
WDW EM
SSB 0
LB 0.30 Hz
GB 0
PC 1.00
12 11 10 9 8 7 6 5 4 3 2 1 ppm
1.00
1.20
2.52
3.69
1.48
4.83
2.97
2.53
3.52

6e

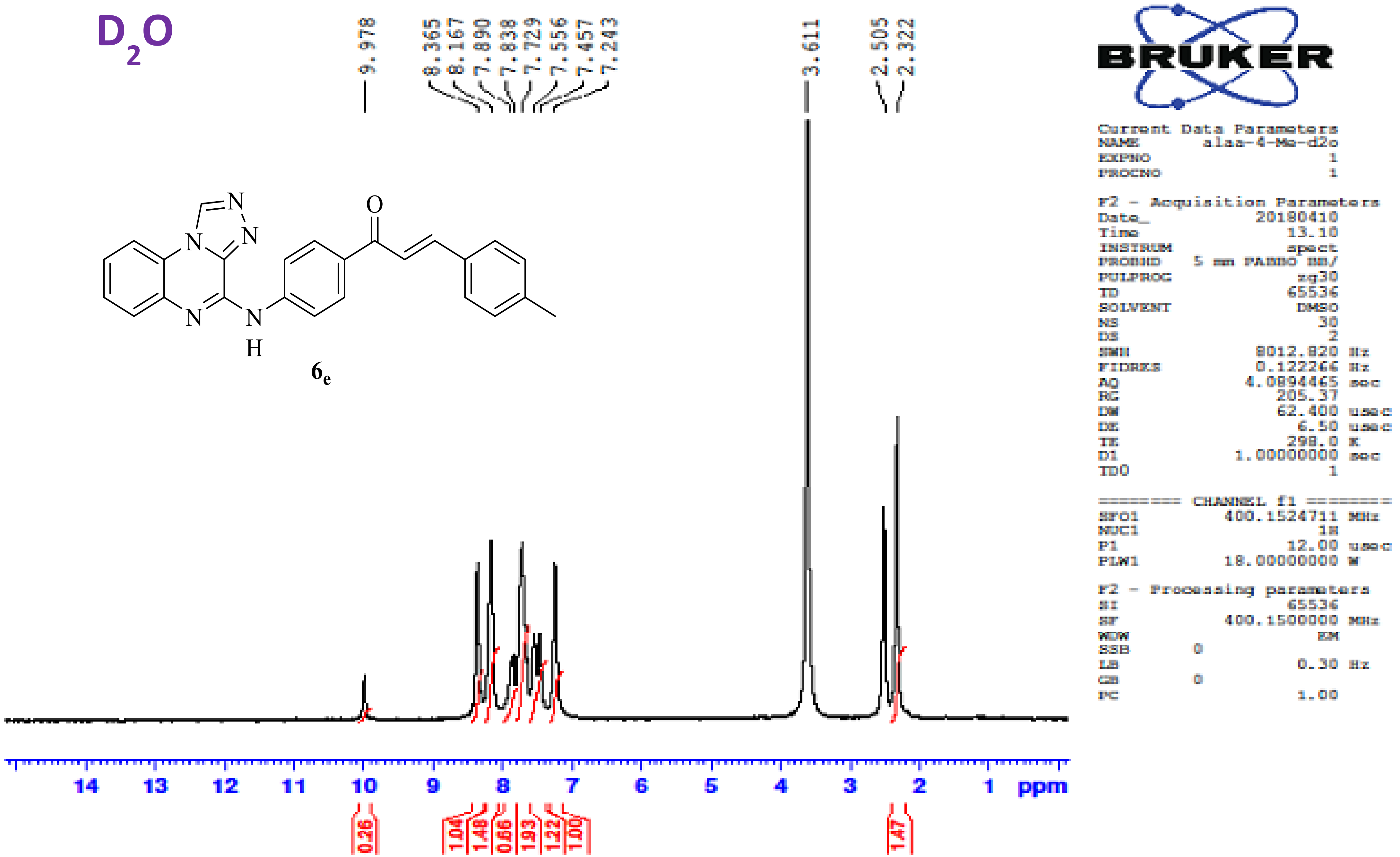

D2O
9.978
8.365
8.167
7.890
7.838
7.729
7.556
7.457
7.243
3.611
2.505
2.322
6e
14 13 12 11 10 9 8 7 6 5 4 3 2 1 ppm
0.26
1.04
1.48
0.66
1.93
1.22
1.00
1.47
BRUKER
Current Data Parameters
NAME alaa-4-Me-d2o
EXPNO 1
PROCNO 1
F2 - Acquisition Parameters
Date_ 20180410
Time 13.10
INSTRUM spect
PROBHD 5 mm PABBO BB/
PULPROG zg30
TD 65536
SOLVENT DMSO
NS 30
DS 2
SWH 8012.820 Hz
FIDRES 0.122266 Hz
AQ 4.0894465 sec
RG 205.37
DW 62.400 usec
DE 6.50 usec
TE 298.0 K
D1 1.00000000 sec
TD0 1
======== CHANNEL f1 ========
SFO1 400.1524711 MHz
NUC1 1H
P1 12.00 usec
PLW1 18.00000000 W
F2 - Processing parameters
SI 65536
SF 400.1500000 MHz
WDW EM
SSB 0
LB 0.30 Hz
GB 0
PC 1.00

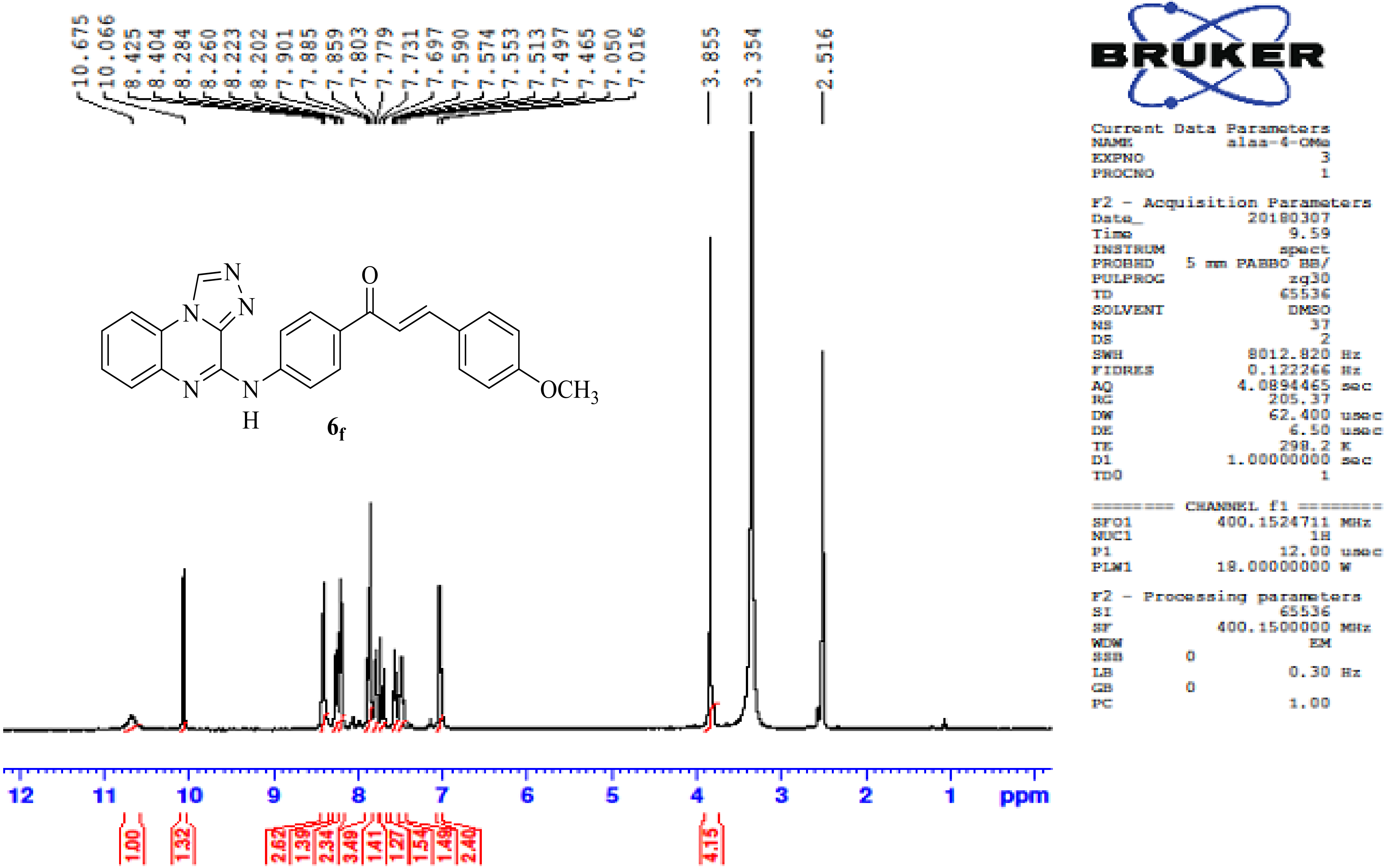
10.675
10.066
8.425
8.404
8.284
8.260
8.223
8.202
7.901
7.885
7.859
7.803
7.779
7.731
7.697
7.590
7.574
7.553
7.513
7.497
7.465
7.050
7.016
3.855
3.354
2.516
N
N
N
N
N
H
O
OCH3
6f
BRUKER
Current Data Parameters
NAME alaa-4-OMe
EXPNO 3
PROCNO 1
F2 - Acquisition Parameters
Date_ 20180307
Time 9.59
INSTRUM spect
PROBHD 5 mm PABBO BB/
PULPROG zg30
TD 65536
SOLVENT DMSO
NS 37
DS 2
SWH 8012.820 Hz
FIDRES 0.122266 Hz
AQ 4.0894465 sec
RG 205.37
DW 62.400 usec
DE 6.50 usec
TE 298.2 K
D1 1.00000000 sec
TD0 1
======== CHANNEL f1 ========
SFO1 400.1524711 MHz
NUC1 1H
P1 12.00 usec
PLW1 18.00000000 W
F2 - Processing parameters
SI 65536
SF 400.1500000 MHz
WDW EM
SSB 0
LB 0.30 Hz
GB 0
PC 1.00
12
11
10
9
8
7
6
5
4
3
2
1
ppm
1.00
1.32
2.62
1.39
2.34
3.49
1.41
1.27
1.54
1.49
2.40
4.15

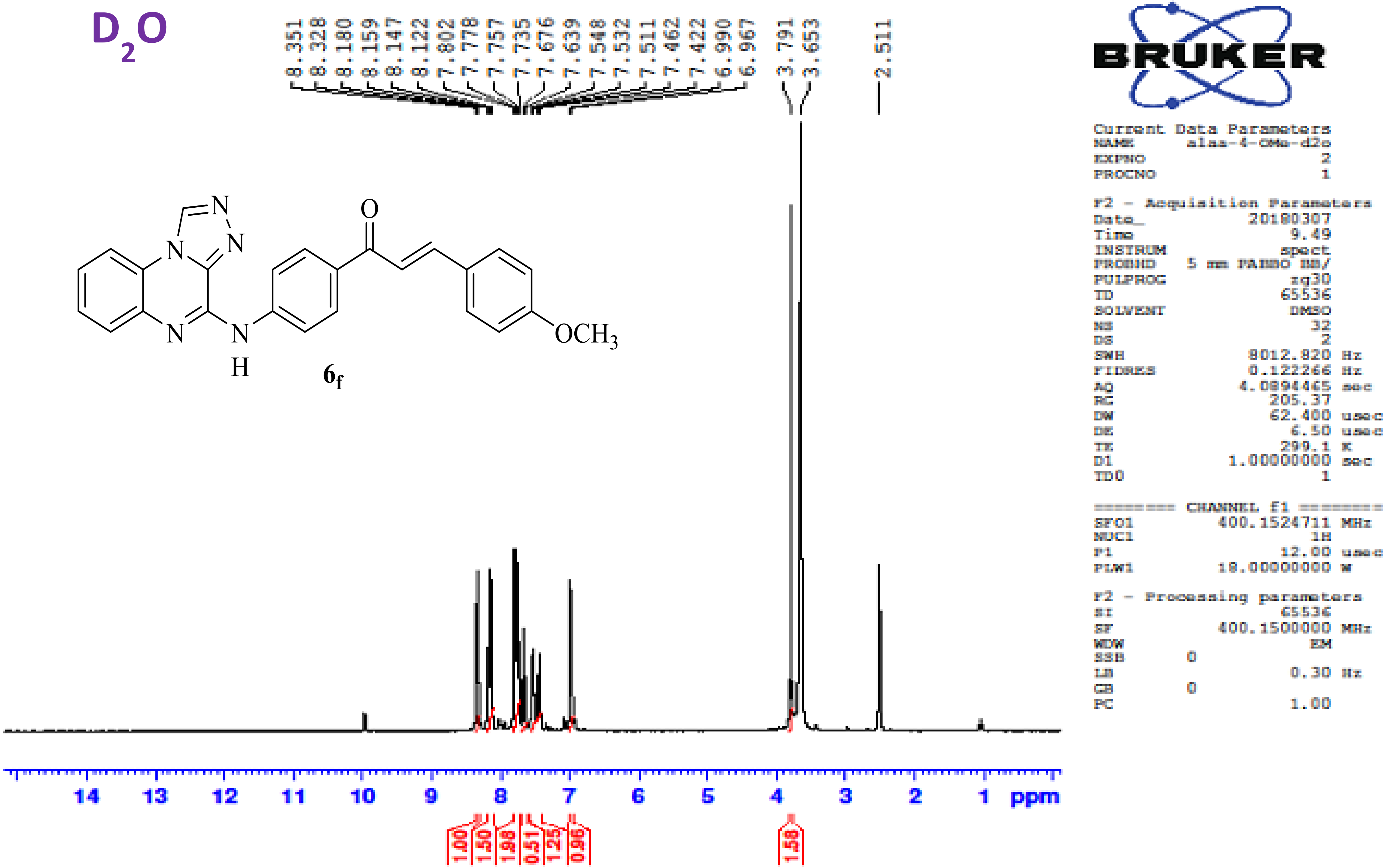

D$_2$O
8.351 8.328 8.180 8.159 8.147 8.122 7.802 7.778 7.757 7.735 7.676 7.639 7.548 7.532 7.511 7.462 7.422 6.990 6.967
3.791 3.653
2.511
OCH$_3$
6$_f$
BRUKER
Current Data Parameters
NAME alaa-4-OMe-d2o
EXPNO 2
PROCNO 1
F2 - Acquisition Parameters
Date_ 20180307
Time 9.49
INSTRUM spect
PROBHD 5 mm PABBO BB/
PULPROG zg30
TD 65536
SOLVENT DMSO
NS 32
DS 2
SWH 8012.820 Hz
FIDRES 0.122266 Hz
AQ 4.0894465 sec
RG 205.37
DW 62.400 usec
DE 6.50 usec
TE 299.1 K
D1 1.00000000 sec
TD0 1
======== CHANNEL f1 ========
SFO1 400.1524711 MHz
NUC1 1H
P1 12.00 usec
PLW1 18.00000000 W
F2 - Processing parameters
SI 65536
SF 400.1500000 MHz
WDW EM
SSB 0
LB 0.30 Hz
GB 0
PC 1.00
14 13 12 11 10 9 8 7 6 5 4 3 2 1 ppm
1.00 1.50 1.98 0.51 1.25 0.96
1.58

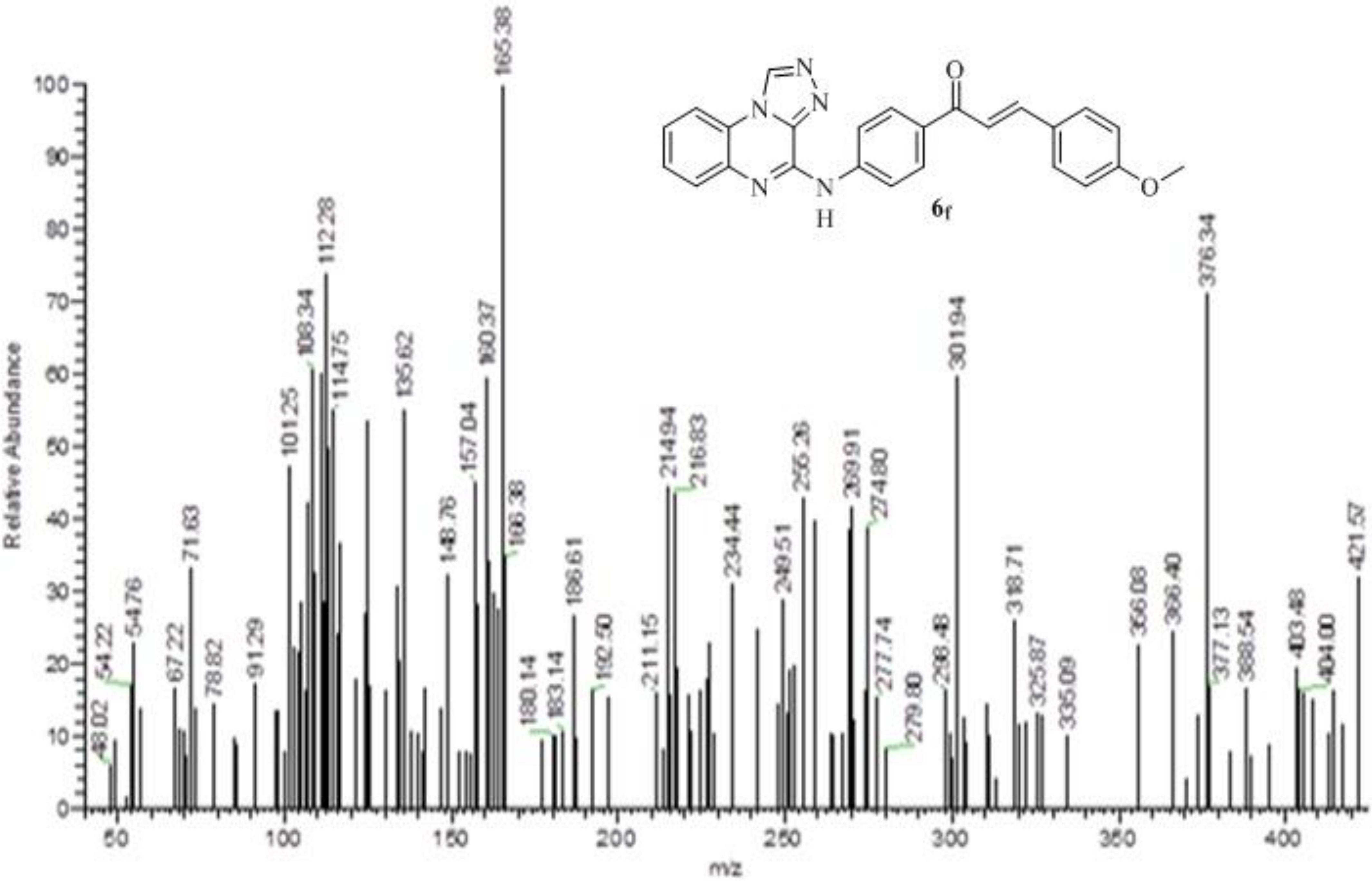
Relative Abundance
m/z
6f
48.02
54.22
54.76
67.22
71.63
78.82
91.29
101.25
108.34
112.28
114.75
135.62
148.76
157.04
160.37
165.38
166.38
180.14
183.14
186.61
192.50
211.15
214.94
216.83
234.44
249.51
256.26
269.91
274.80
277.74
279.80
298.48
301.94
318.71
325.87
335.09
356.08
366.40
376.34
377.13
388.54
403.48
404.00
421.57

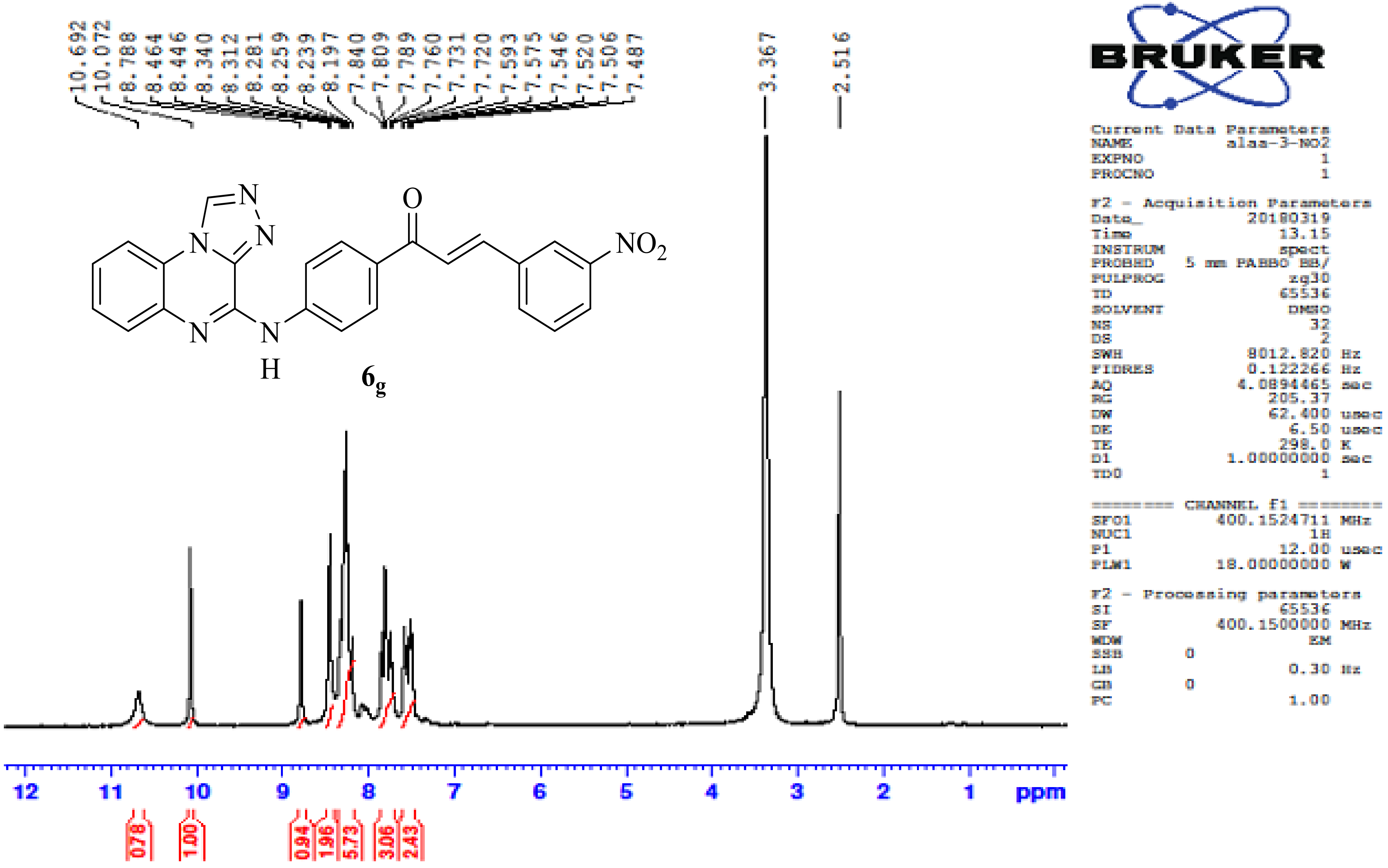
10.692
10.072
8.788
8.464
8.446
8.340
8.312
8.281
8.259
8.239
8.197
7.840
7.809
7.789
7.760
7.731
7.720
7.593
7.575
7.546
7.520
7.506
7.487
3.367
2.516
N
N
N
N
N
H
O
NO2
6g
BRUKER
Current Data Parameters
NAME alaa-3-NO2
EXPNO 1
PROCNO 1
F2 - Acquisition Parameters
Date_ 20180319
Time 13.15
INSTRUM spect
PROBHD 5 mm PABBO BB/
PULPROG zg30
TD 65536
SOLVENT DMSO
NS 32
DS 2
SWH 8012.820 Hz
FIDRES 0.122266 Hz
AQ 4.0894465 sec
RG 205.37
DW 62.400 usec
DE 6.50 usec
TE 298.0 K
D1 1.00000000 sec
TD0 1
======== CHANNEL f1 ========
SFO1 400.1524711 MHz
NUC1 1H
P1 12.00 usec
PLW1 18.00000000 W
F2 - Processing parameters
SI 65536
SF 400.1500000 MHz
WDW EM
SSB 0
LB 0.30 Hz
GB 0
PC 1.00
12
11
10
9
8
7
6
5
4
3
2
1
ppm
0.78
1.00
0.94
1.96
5.73
3.06
2.43

Alaa ElWan_C_3-NO2-Ch

Microanalytical Unit - FOPCU - NMR laboratory
www.pharma.cu.edu.eg dir-mau.fopcu@pharma.cu.edu.eg

BRUKER

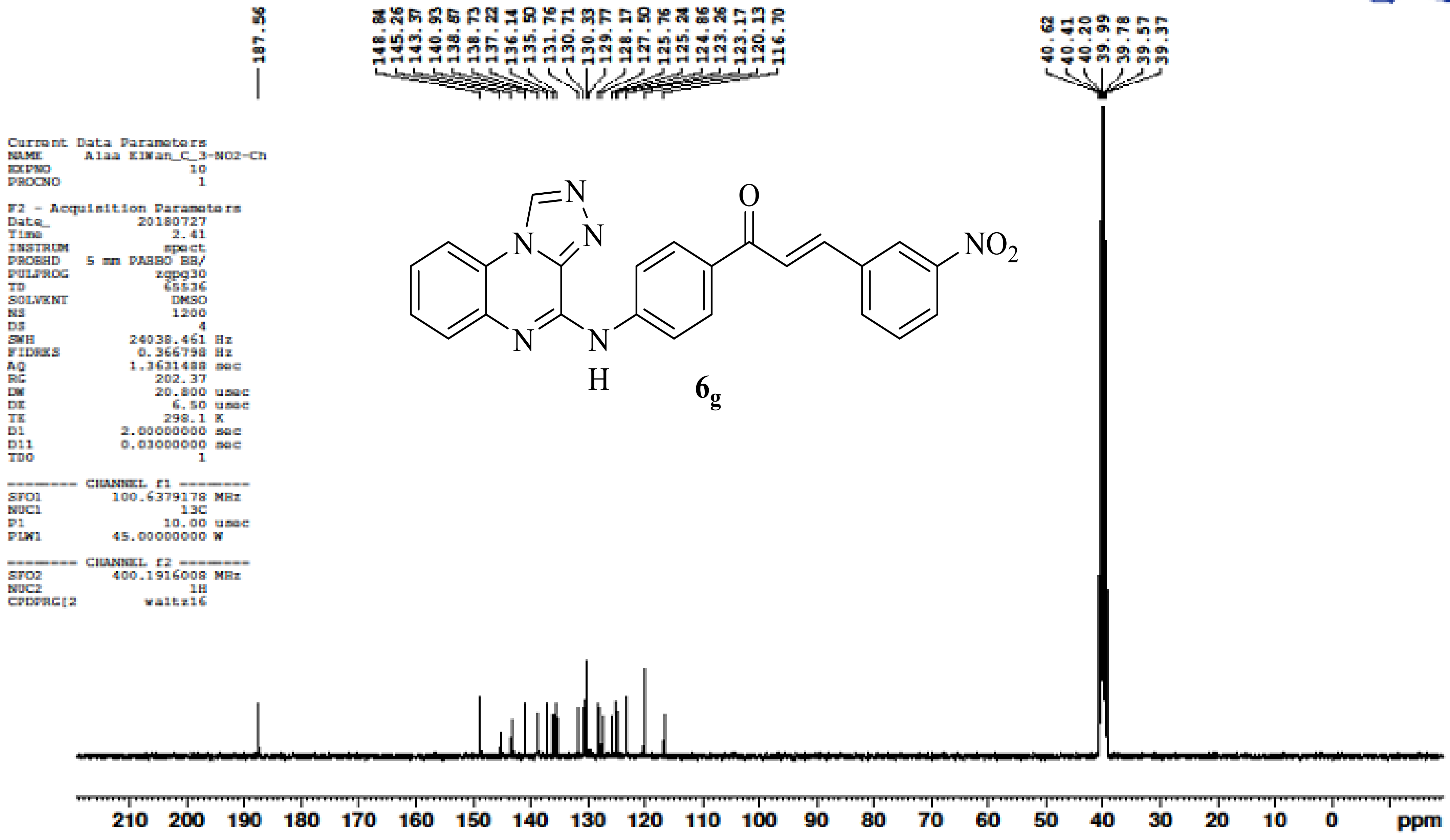

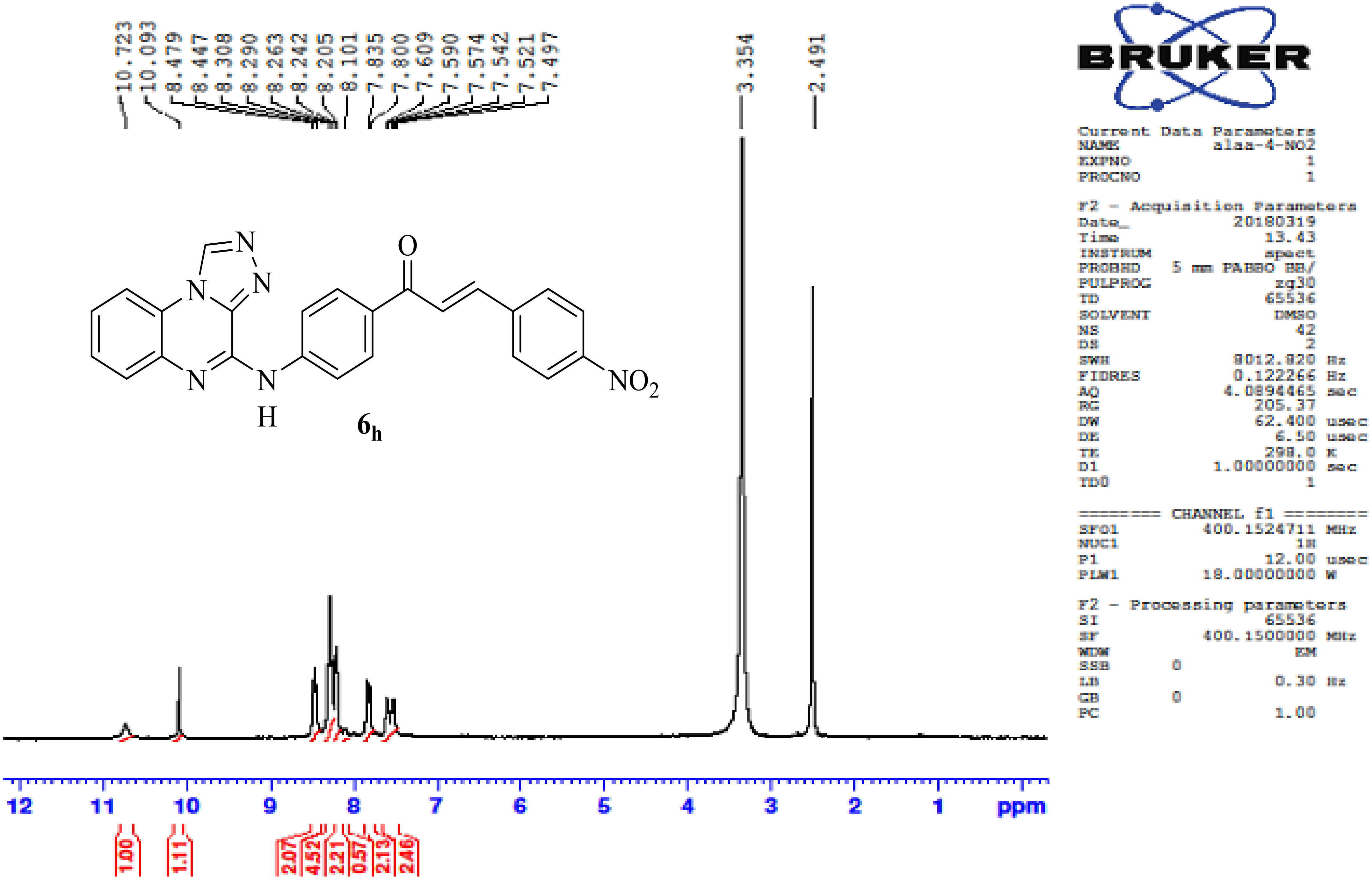

10.723
10.093
8.479
8.447
8.308
8.290
8.263
8.242
8.205
8.101
7.835
7.800
7.609
7.590
7.574
7.542
7.521
7.497
3.354
2.491
N
N
N
O
N
N
H
NO2
6h
BRUKER
Current Data Parameters
NAME alaa-4-NO2
EXPNO 1
PROCNO 1
F2 - Acquisition Parameters
Date_ 20180319
Time 13.43
INSTRUM spect
PROBHD 5 mm PABBO BB/
PULPROG zg30
TD 65536
SOLVENT DMSO
NS 42
DS 2
SWH 8012.820 Hz
FIDRES 0.122266 Hz
AQ 4.0894465 sec
RG 205.37
DW 62.400 usec
DE 6.50 usec
TE 298.0 K
D1 1.00000000 sec
TD0 1
======== CHANNEL f1 ========
SFO1 400.1524711 MHz
NUC1 1H
P1 12.00 usec
PLW1 18.00000000 W
F2 - Processing parameters
SI 65536
SF 400.1500000 MHz
WDW EM
SSB 0
LB 0.30 Hz
GB 0
PC 1.00
12
11
10
9
8
7
6
5
4
3
2
1
ppm
1.00
1.11
2.07
4.52
2.21
0.57
2.13
2.46

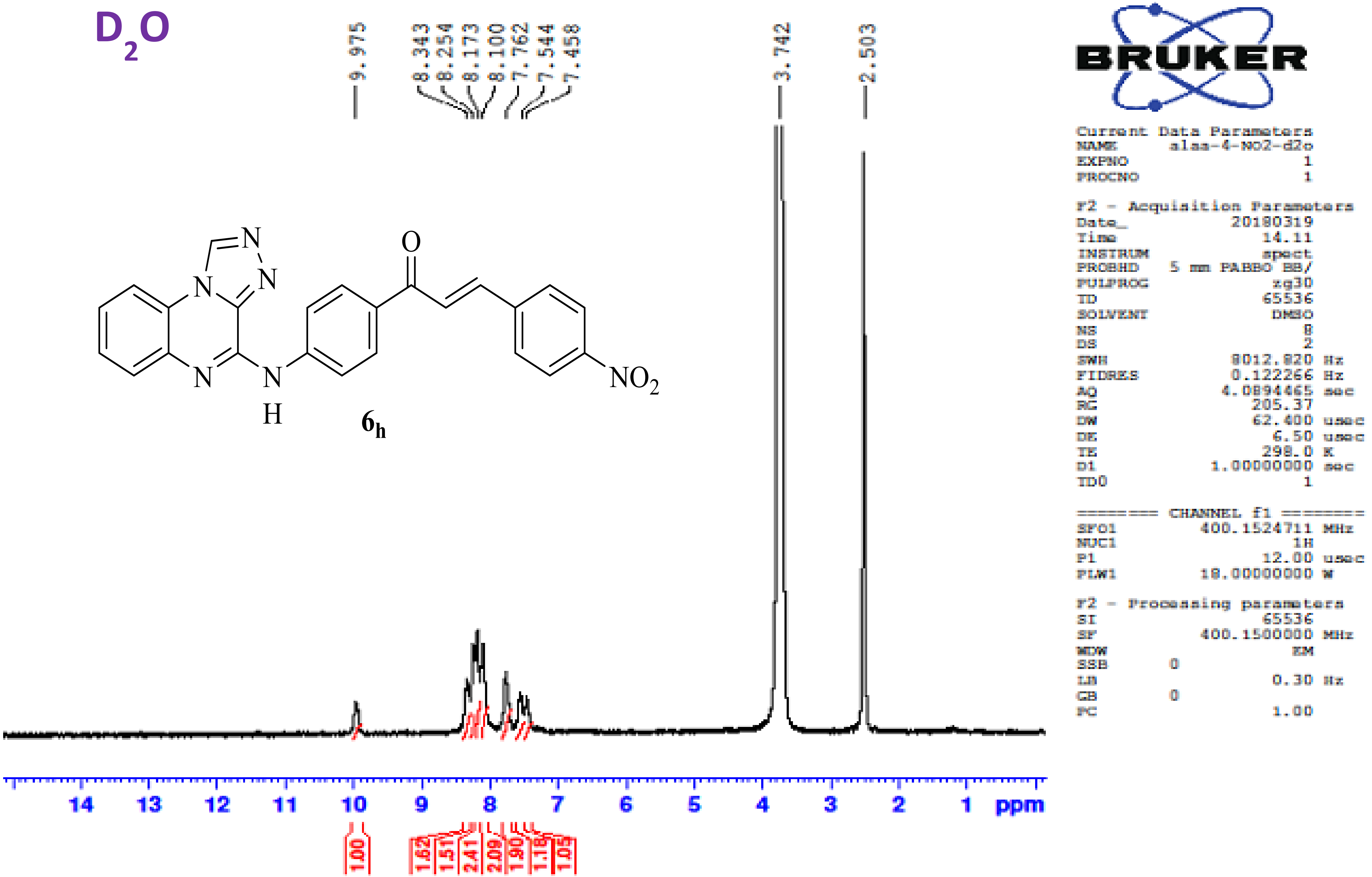

D2O
9.975
8.343
8.254
8.173
8.100
7.762
7.544
7.458
3.742
2.503
N
N
N
N
N
N
H
O
NO2
6h
14 13 12 11 10 9 8 7 6 5 4 3 2 1 ppm
1.00
1.62
1.51
2.41
2.09
1.90
1.18
1.05
BRUKER
Current Data Parameters
NAME alas-4-NO2-d2o
EXPNO 1
PROCNO 1
F2 - Acquisition Parameters
Date_ 20180319
Time 14.11
INSTRUM spect
PROBHD 5 mm PABBO BB/
PULPROG zg30
TD 65536
SOLVENT DMSO
NS 8
DS 2
SWH 8012.820 Hz
FIDRES 0.122266 Hz
AQ 4.0894465 sec
RG 205.37
DW 62.400 usec
DE 6.50 usec
TE 298.0 K
D1 1.00000000 sec
TD0 1
======== CHANNEL f1 ========
SFO1 400.1524711 MHz
NUC1 1H
P1 12.00 usec
PLW1 18.00000000 W
F2 - Processing parameters
SI 65536
SF 400.1500000 MHz
WDW EM
SSB 0
LB 0.30 Hz
GB 0
PC 1.00

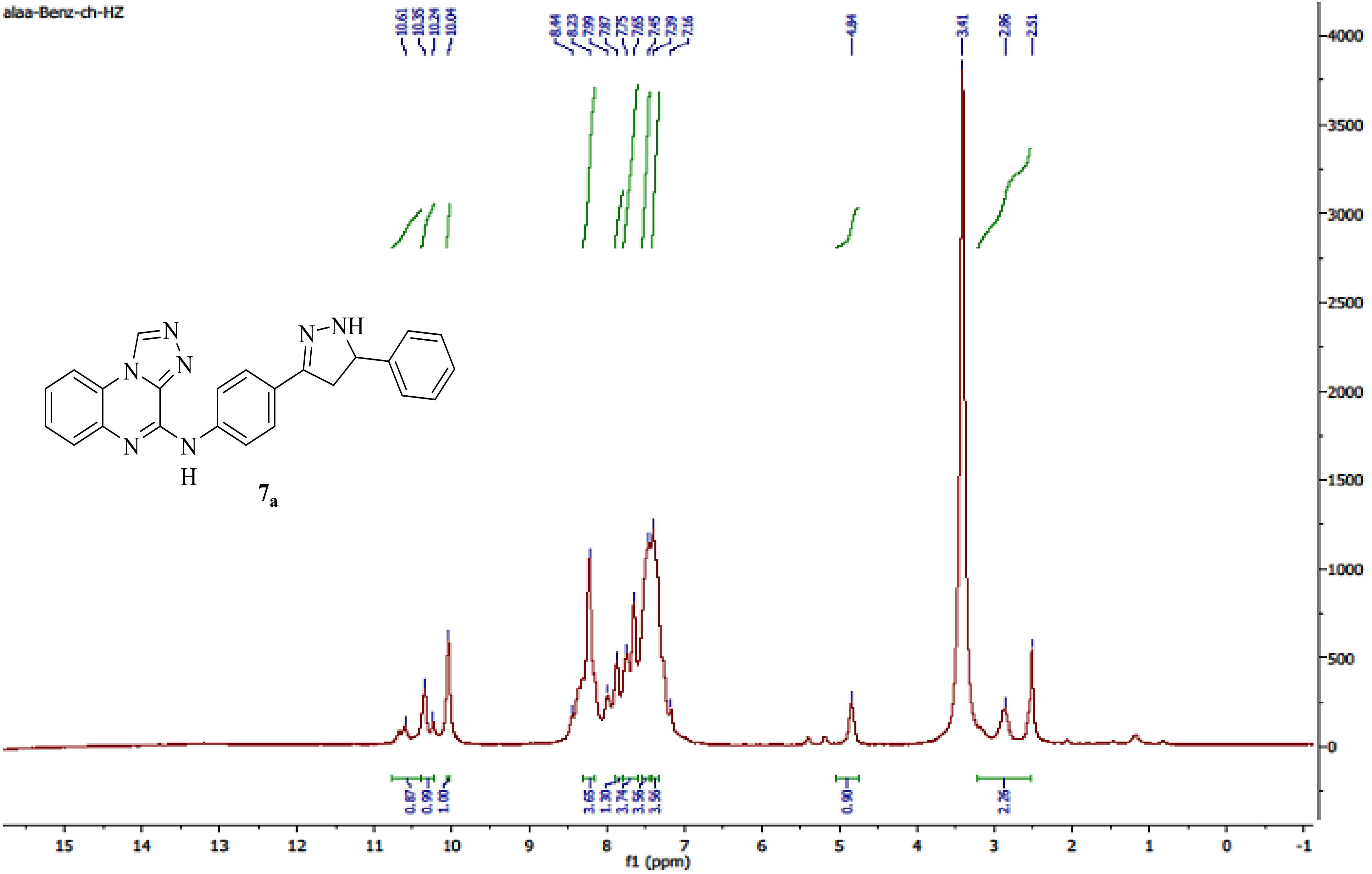

alaa-Benz-ch-HZ
10.61
10.35
10.24
10.04
8.44
8.23
7.99
7.87
7.75
7.65
7.45
7.39
7.16
4.84
3.41
2.86
2.51
0.87
0.99
1.00
3.65
1.30
3.74
3.56
3.56
0.90
2.26
f1 (ppm)
7a

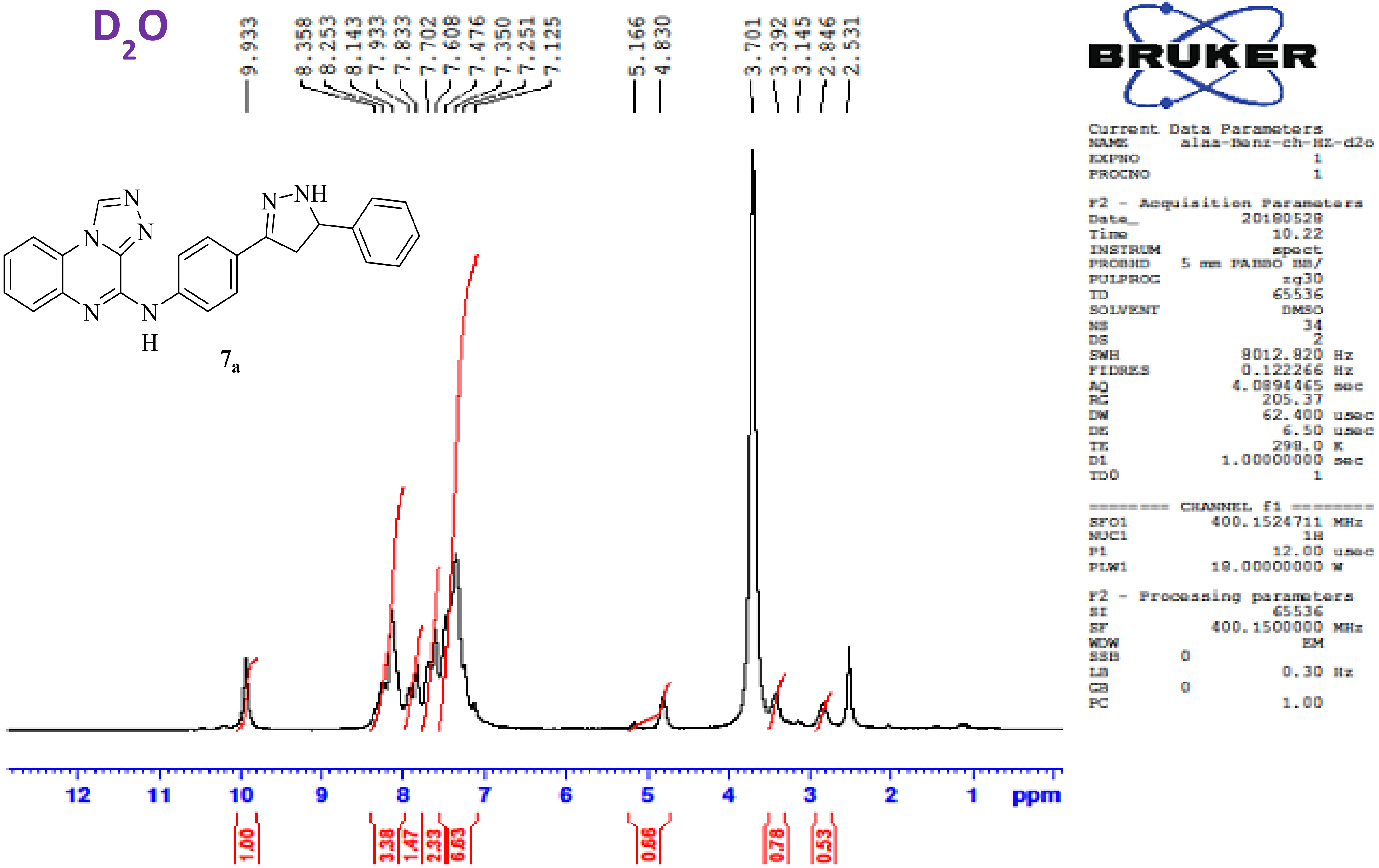

D2O
9.933
8.358
8.253
8.143
7.933
7.833
7.702
7.608
7.476
7.350
7.251
7.125
5.166
4.830
3.701
3.392
3.145
2.846
2.531
N
N
N
N
N
H
N
NH
7a
BRUKER
Current Data Parameters
NAME alaa-Benz-ch-HZ-d2o
EXPNO 1
PROCNO 1
F2 - Acquisition Parameters
Date_ 20180528
Time 10.22
INSTRUM spect
PROBHD 5 mm PABBO BB/
PULPROG zg30
TD 65536
SOLVENT DMSO
NS 34
DS 2
SWH 8012.820 Hz
FIDRES 0.122266 Hz
AQ 4.0894465 sec
RG 205.37
DW 62.400 usec
DE 6.50 usec
TE 298.0 K
D1 1.00000000 sec
TD0 1
======== CHANNEL f1 ========
SFO1 400.1524711 MHz
NUC1 1H
P1 12.00 usec
PLW1 18.00000000 W
F2 - Processing parameters
SI 65536
SF 400.1500000 MHz
WDW EM
SSB 0
LB 0.30 Hz
GB 0
PC 1.00
12
11
10
9
8
7
6
5
4
3
2
1
ppm
1.00
3.38
1.47
2.33
6.63
0.66
0.78
0.53

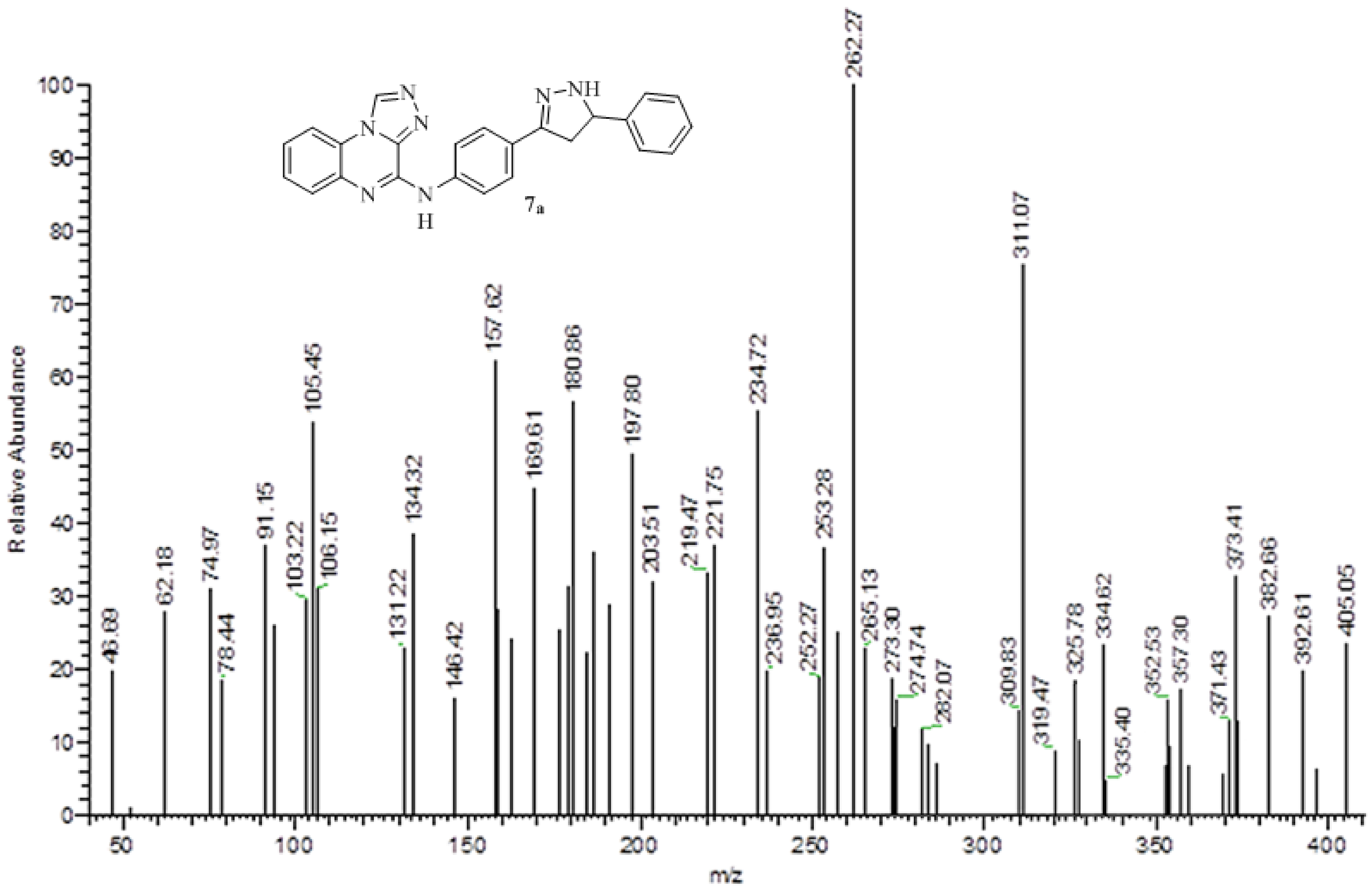
7a
Relative Abundance
m/z
262.27
311.07
157.62
180.86
105.45
234.72
169.61
197.80
134.32
253.28
221.75
91.15
203.51
219.47
373.41
106.15
103.22
382.66
74.97
62.18
265.13
131.22
334.62
405.05
46.69
78.44
236.95
252.27
273.30
274.74
146.42
282.07
309.83
319.47
325.78
335.40
352.53
357.30
371.43
392.61

alaa-elwan-4-cl-ch-hz #253 RT: 4.25 AV: 1 SB: 5 2.73 , 2.71-2.76 NL: 1.64E3
T: {0,0} + c EI Full ms [40.00-1000.00]

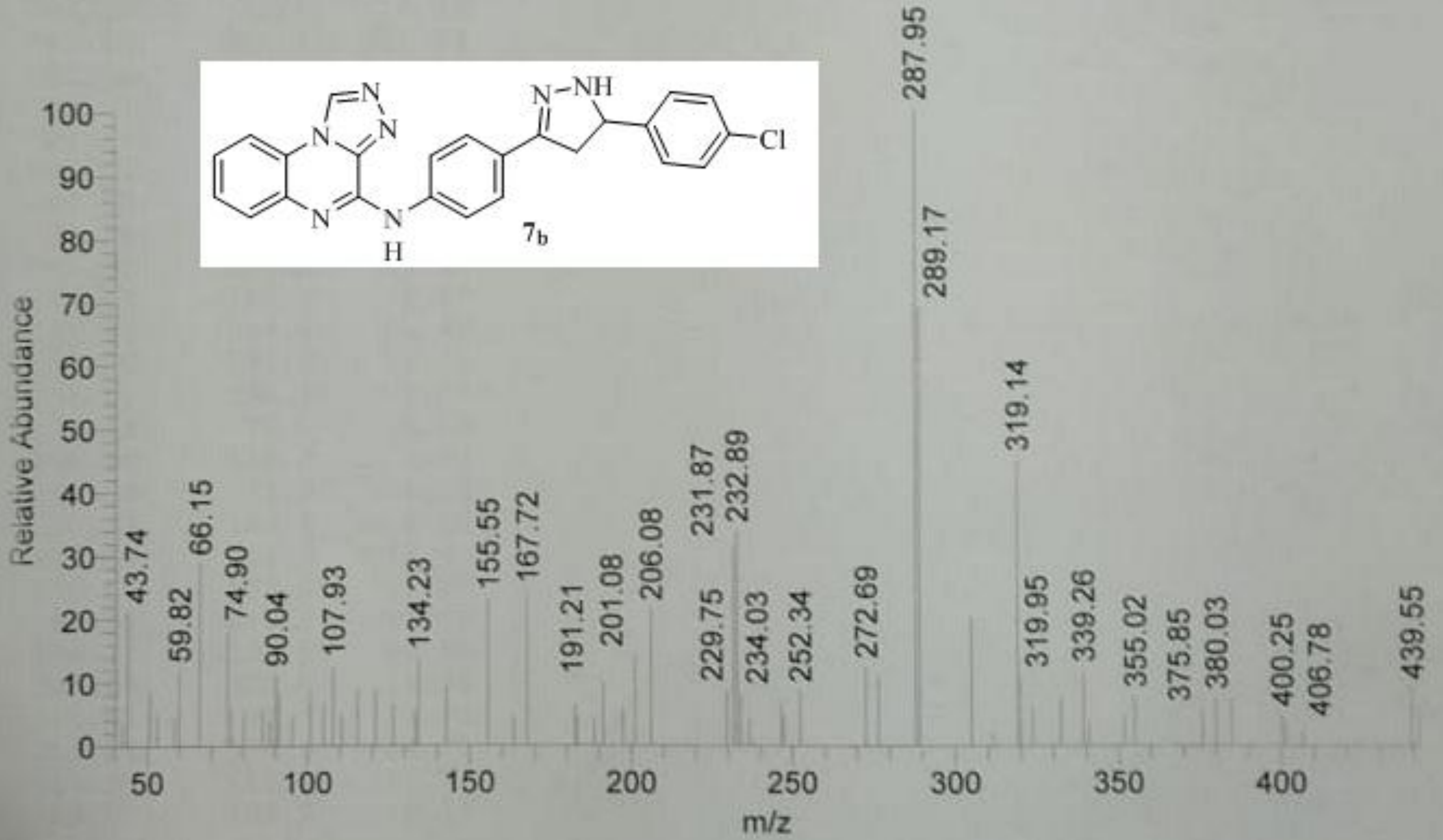

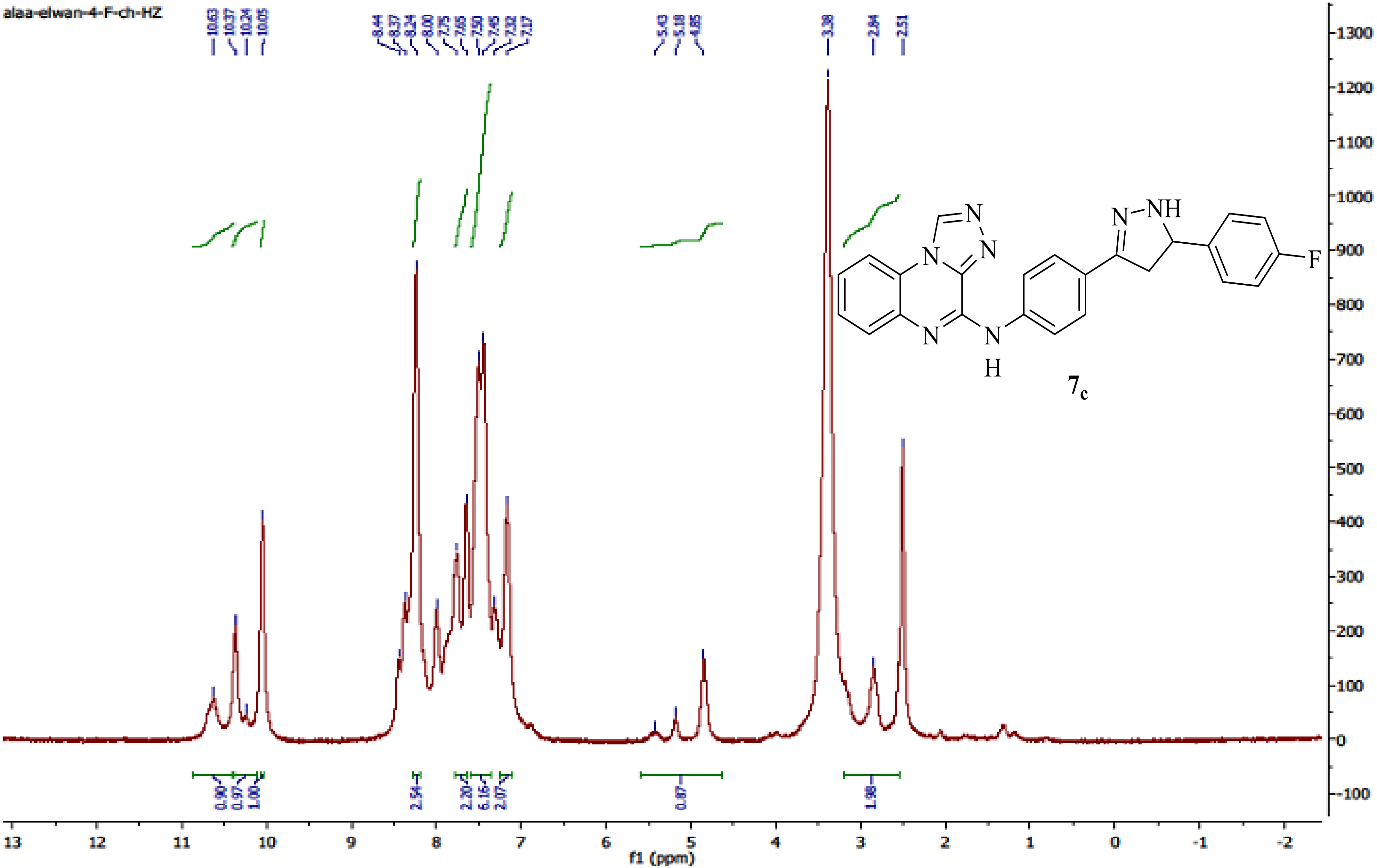
alaa-elwan-4-F-ch-HZ
10.63
10.37
10.24
10.05
8.44
8.37
8.24
8.00
7.75
7.65
7.50
7.45
7.32
7.17
5.43
5.18
4.85
3.38
2.84
2.51
0.90
0.97
1.00
2.54
2.20
6.16
2.07
0.87
1.98
N
N
N
N
N
N
NH
H
F
7c
f1 (ppm)

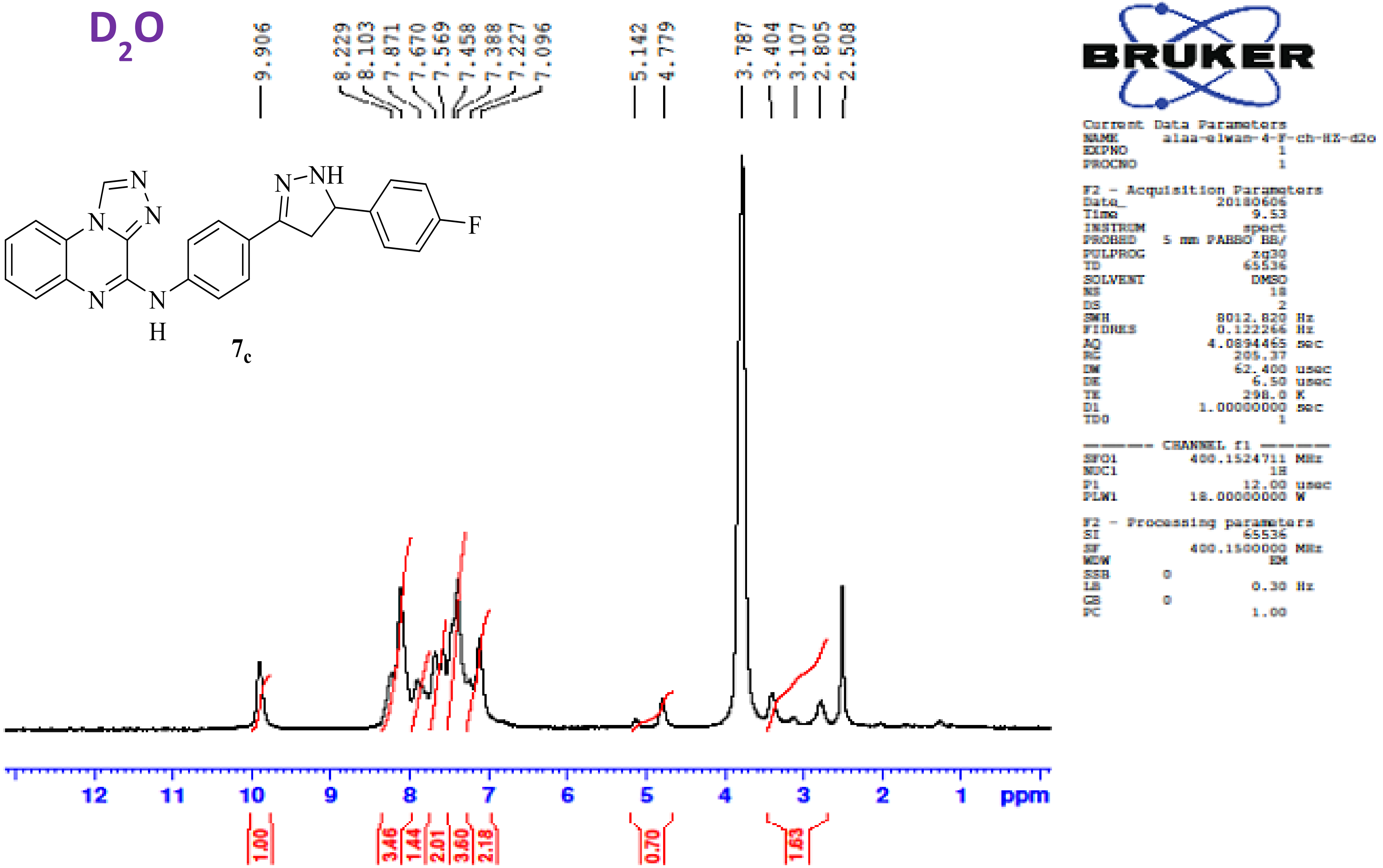
D2O
9.906
8.229
8.103
7.871
7.670
7.569
7.458
7.388
7.227
7.096
5.142
4.779
3.787
3.404
3.107
2.805
2.508
N
N
N
N
N
H
N
NH
F
7c
BRUKER
Current Data Parameters
NAME alaa-elwan-4-F-ch-HZ-d2o
EXPNO 1
PROCNO 1
F2 - Acquisition Parameters
Date_ 20180606
Time 9.53
INSTRUM spect
PROBHD 5 mm PABBO BB/
PULPROG zg30
TD 65536
SOLVENT DMSO
NS 18
DS 2
SWH 8012.820 Hz
FIDRES 0.122266 Hz
AQ 4.0894465 sec
RG 205.37
DW 62.400 usec
DE 6.50 usec
TE 298.0 K
D1 1.00000000 sec
TD0 1
CHANNEL f1
SFO1 400.1524711 MHz
NUC1 1H
P1 12.00 usec
PLW1 18.00000000 W
F2 - Processing parameters
SI 65536
SF 400.1500000 MHz
WDW EM
SSB 0
LB 0.30 Hz
GB 0
PC 1.00
12
11
10
9
8
7
6
5
4
3
2
1
ppm
1.00
3.46
1.44
2.01
3.60
2.18
0.70
1.63

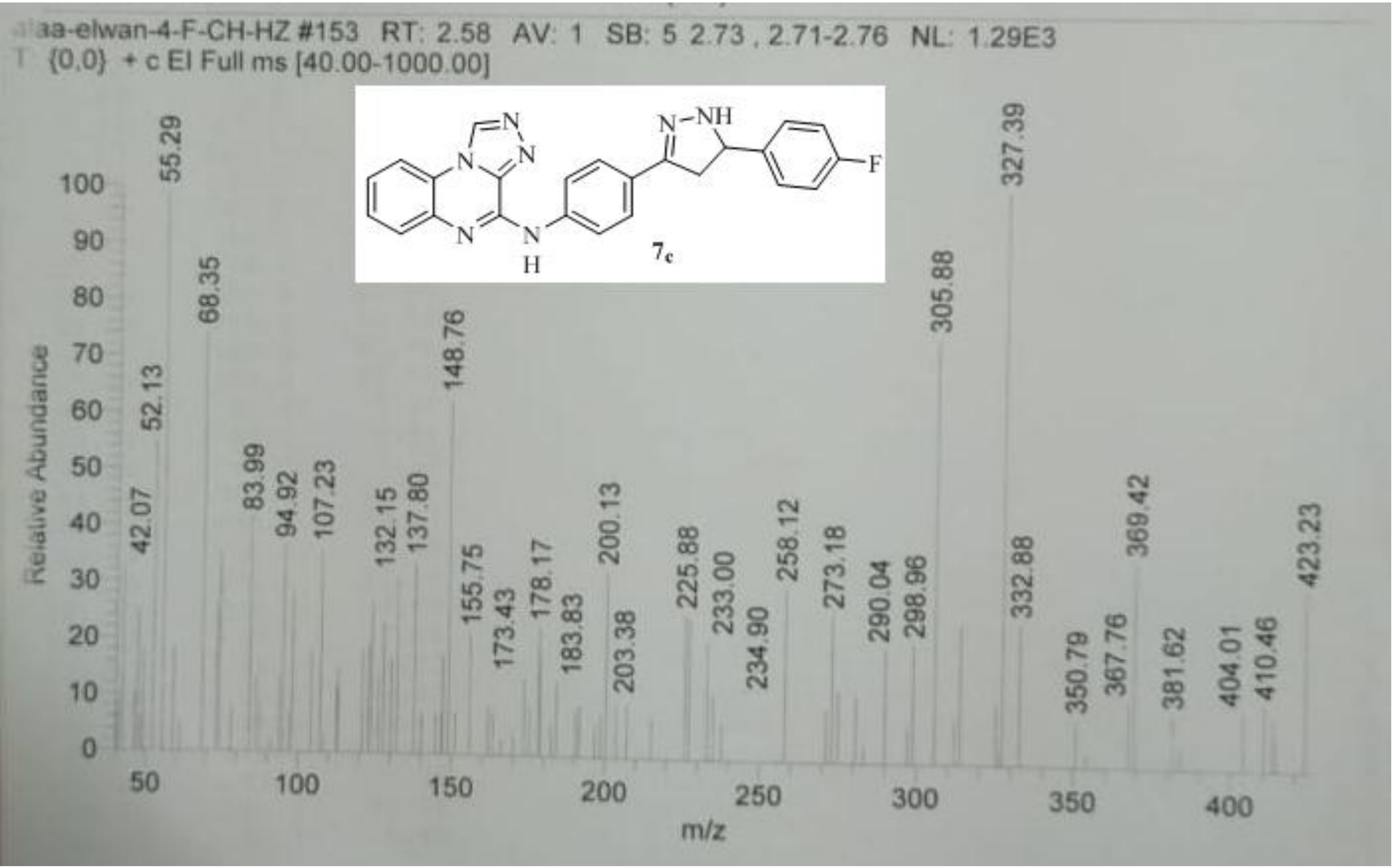
aa-elwan-4-F-CH-HZ #153 RT: 2.58 AV: 1 SB: 5 2.73 , 2.71-2.76 NL: 1.29E3
{0,0} + c EI Full ms [40.00-1000.00]
N
N
N
N
N
H
N-NH
F
7c
Relative Abundance
m/z
55.29
52.13
42.07
68.35
83.99
94.92
107.23
132.15
137.80
148.76
155.75
173.43
178.17
183.83
200.13
203.38
225.88
233.00
234.90
258.12
273.18
290.04
298.96
305.88
327.39
332.88
350.79
367.76
369.42
381.62
404.01
410.46
423.23

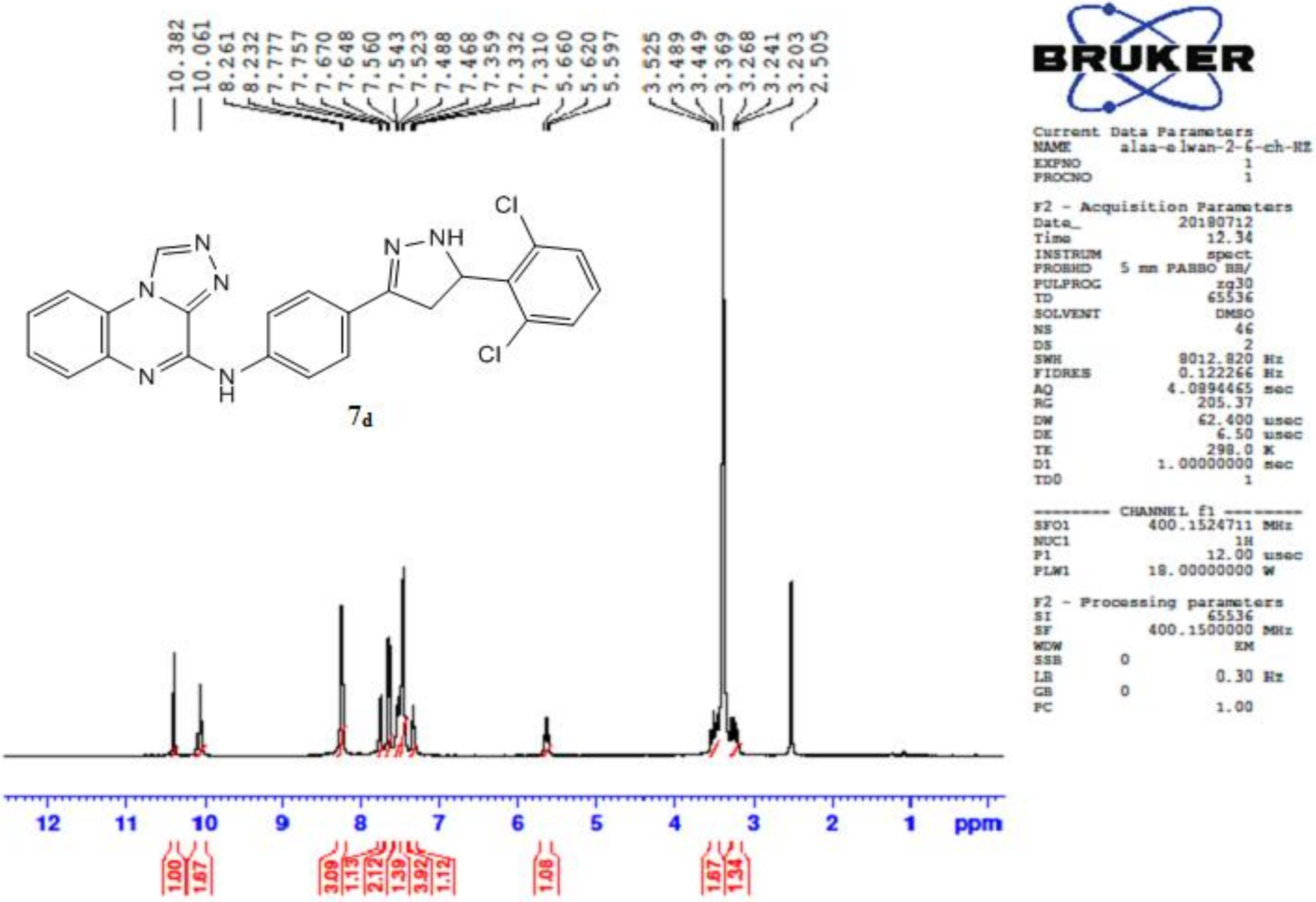

10.382 10.061 8.261 8.232 7.777 7.757 7.670 7.648 7.560 7.543 7.523 7.488 7.468 7.359 7.332 7.310 5.660 5.620 5.597
3.525 3.489 3.449 3.369 3.268 3.241 3.203 2.505
Cl
N
NH
N
N
N
Cl
N
N
H
7d
BRUKER
Current Data Parameters
NAME alaa-elwan-2-6-ch-HZ
EXPNO 1
PROCNO 1
F2 - Acquisition Parameters
Date_ 20180712
Time 12.34
INSTRUM spect
PROBHD 5 mm PABBO BB/
PULPROG zg30
TD 65536
SOLVENT DMSO
NS 46
DS 2
SWH 8012.820 Hz
FIDRES 0.122266 Hz
AQ 4.0894465 sec
RG 205.37
DW 62.400 usec
DE 6.50 usec
TE 298.0 K
D1 1.00000000 sec
TD0 1
======== CHANNEL f1 ========
SFO1 400.1524711 MHz
NUC1 1H
P1 12.00 usec
PLW1 18.00000000 W
F2 - Processing parameters
SI 65536
SF 400.1500000 MHz
WDW EM
SSB 0
LB 0.30 Hz
GB 0
PC 1.00
12 11 10 9 8 7 6 5 4 3 2 1 ppm
1.00 1.67 3.09 1.13 2.12 1.39 3.92 1.12 1.08 1.67 1.34

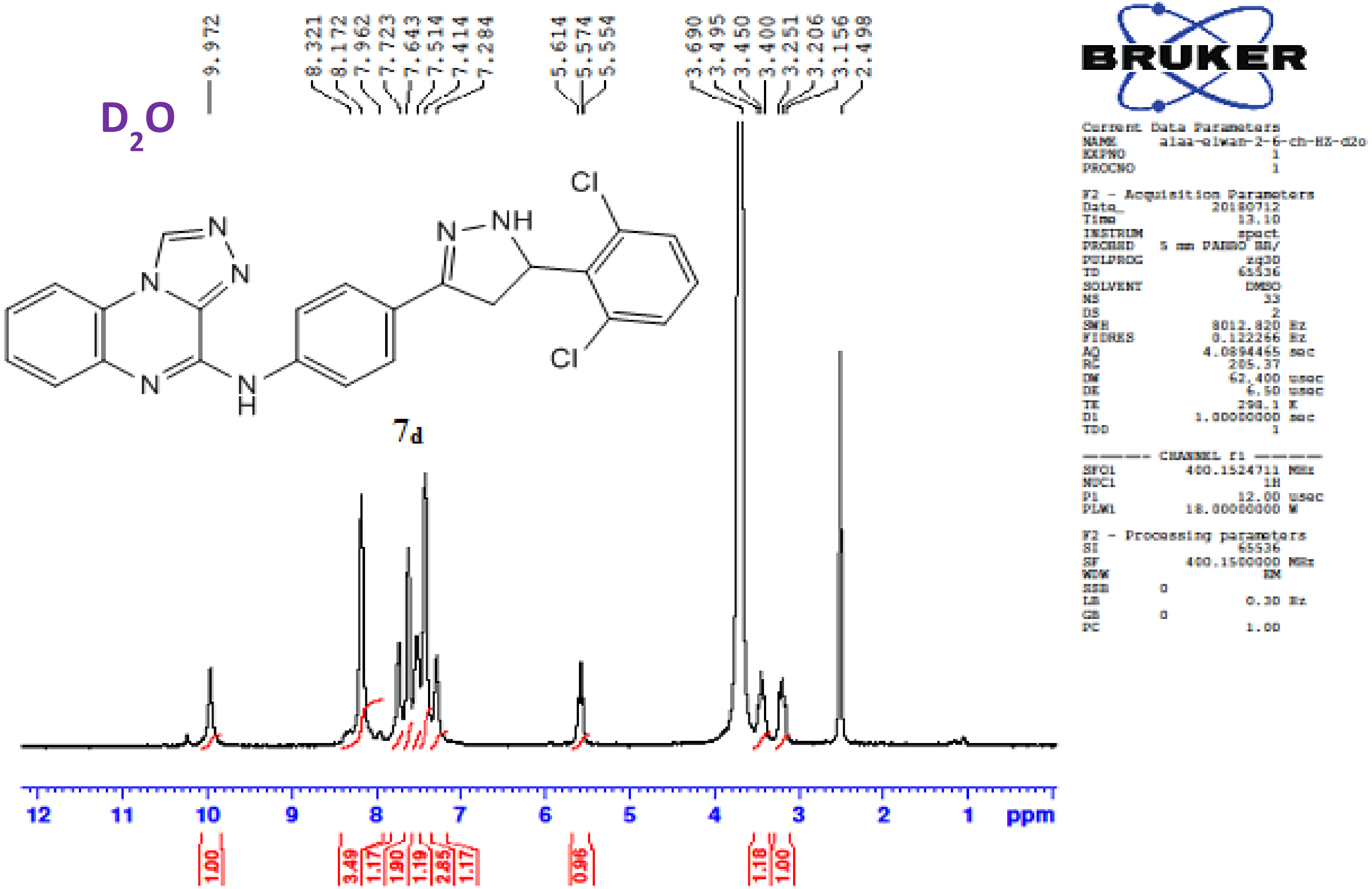
D2O
9.972
8.321 8.172 7.962 7.723 7.643 7.514 7.414 7.284
5.614 5.574 5.554
3.690 3.495 3.450 3.400 3.251 3.206 3.156 2.498
Cl
N—NH
N
N
N
N
N
H
Cl
7d
12 11 10 9 8 7 6 5 4 3 2 1 ppm
1.00
3.49 1.17 1.90 1.19 2.85 1.17
0.96
1.18 1.00
BRUKER
Current Data Parameters
NAME alaa-alwan-2-6-ch-HZ-d2o
EXPNO 1
PROCNO 1
F2 - Acquisition Parameters
Date_ 20180712
Time 13.10
INSTRUM spect
PROBHD 5 mm PABBO BB/
PULPROG zg30
TD 65536
SOLVENT DMSO
NS 33
DS 2
SWH 8012.820 Hz
FIDRES 0.122266 Hz
AQ 4.0894465 sec
RG 205.37
DW 62.400 usec
DE 6.50 usec
TE 298.1 K
D1 1.00000000 sec
TD0 1
CHANNEL f1
SFO1 400.1524711 MHz
NUC1 1H
P1 12.00 usec
PLW1 16.00000000 W
F2 - Processing parameters
SI 65536
SF 400.1500000 MHz
WDW EM
SSB 0
LB 0.30 Hz
GB 0
PC 1.00

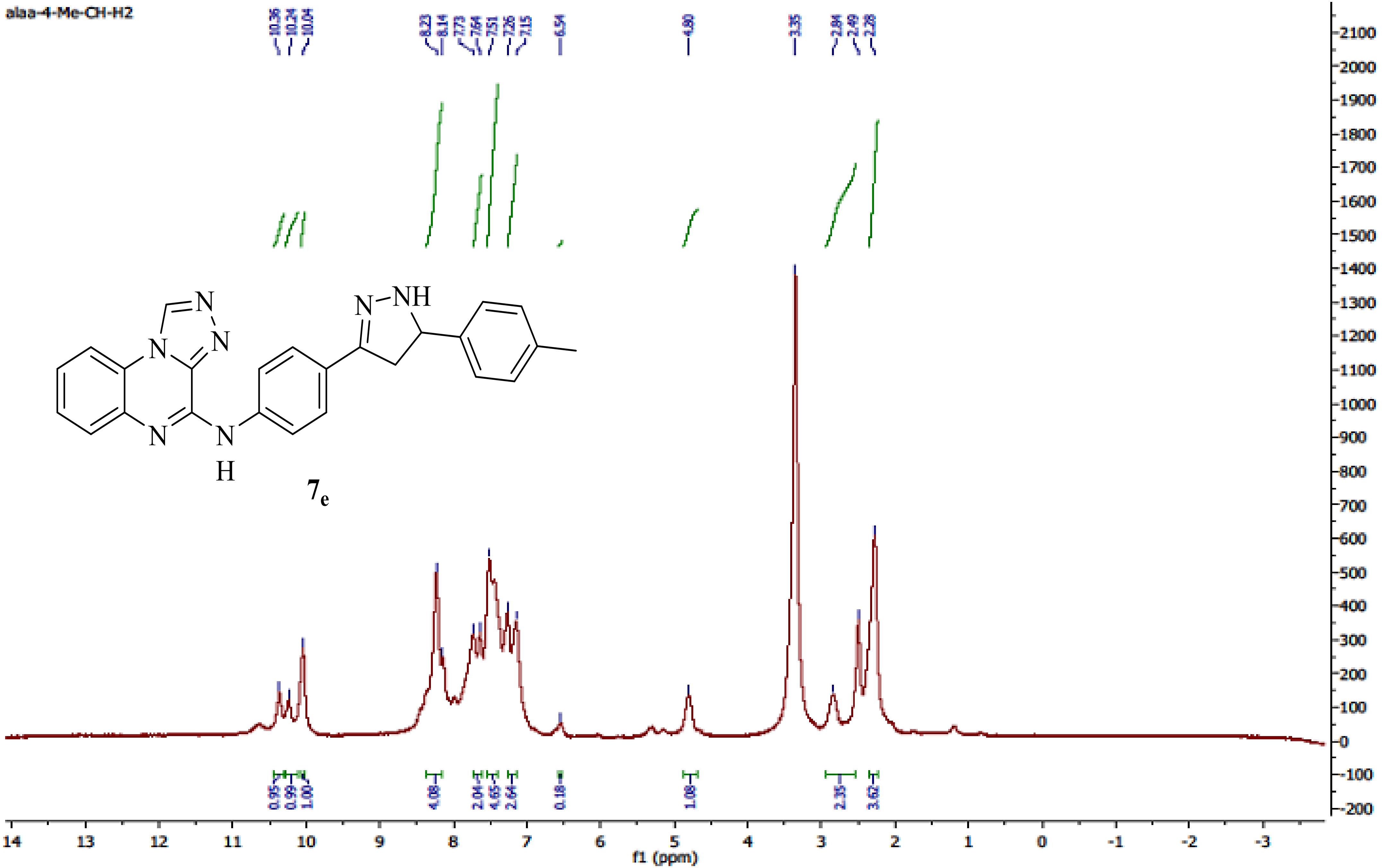
alaa-4-Me-CH-H2
10.36
10.24
10.04
8.23
8.14
7.73
7.64
7.51
7.26
7.15
6.54
4.80
3.35
2.84
2.49
2.28
N
N
N
N
N
H
N
NH
7_e
0.95
0.99
1.00
4.08
2.04
4.65
2.64
0.18
1.08
2.35
3.62
f1 (ppm)

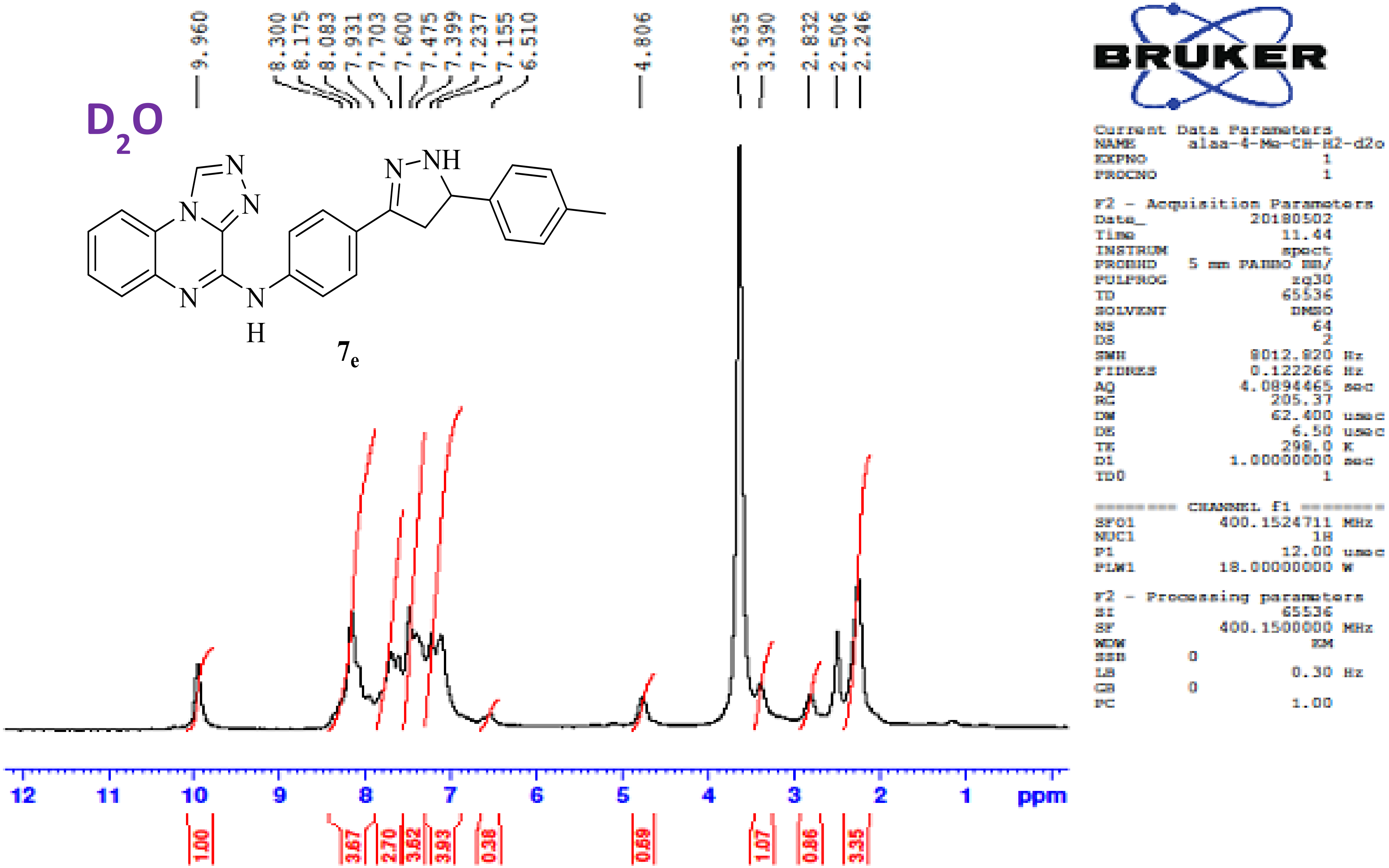

D2O
7e
9.960
8.300
8.175
8.083
7.931
7.703
7.600
7.475
7.399
7.237
7.155
6.510
4.806
3.635
3.390
2.832
2.506
2.246
12 11 10 9 8 7 6 5 4 3 2 1 ppm
1.00
3.67
2.70
3.62
3.93
0.38
0.69
1.07
0.86
3.35
BRUKER
Current Data Parameters
NAME alaa-4-Me-CH-H2-d2o
EXPNO 1
PROCNO 1
F2 - Acquisition Parameters
Date_ 20180502
Time 11.44
INSTRUM spect
PROBHD 5 mm PABBO BB/
PULPROG zg30
TD 65536
SOLVENT DMSO
NS 64
DS 2
SWH 8012.820 Hz
FIDRES 0.122266 Hz
AQ 4.0894465 sec
RG 205.37
DW 62.400 usec
DE 6.50 usec
TE 298.0 K
D1 1.00000000 sec
TD0 1
CHANNEL f1
SFO1 400.1524711 MHz
NUC1 1H
P1 12.00 usec
PLW1 18.00000000 W
F2 - Processing parameters
SI 65536
SF 400.1500000 MHz
WDW EM
SSB 0
LB 0.30 Hz
GB 0
PC 1.00

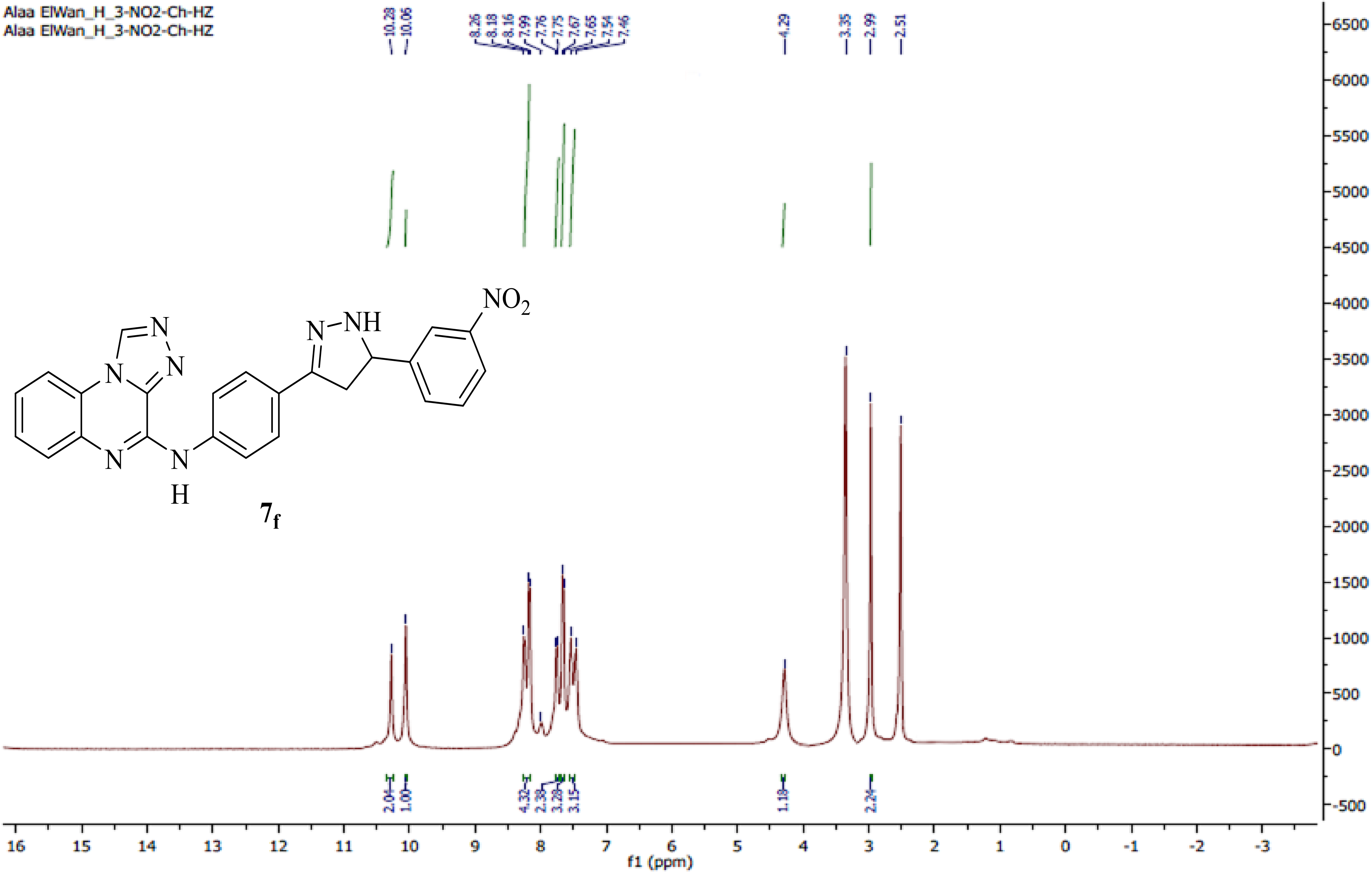
Alaa ElWan_H_3-NO2-Ch-HZ
Alaa ElWan_H_3-NO2-Ch-HZ
10.28
10.06
8.26
8.18
8.16
7.99
7.76
7.75
7.67
7.65
7.54
7.46
4.29
3.35
2.99
2.51
NO2
N-NH
N
N
N
N
N
H
7f
2.04
1.00
4.32
2.38
3.28
3.15
1.18
2.24
f1 (ppm)

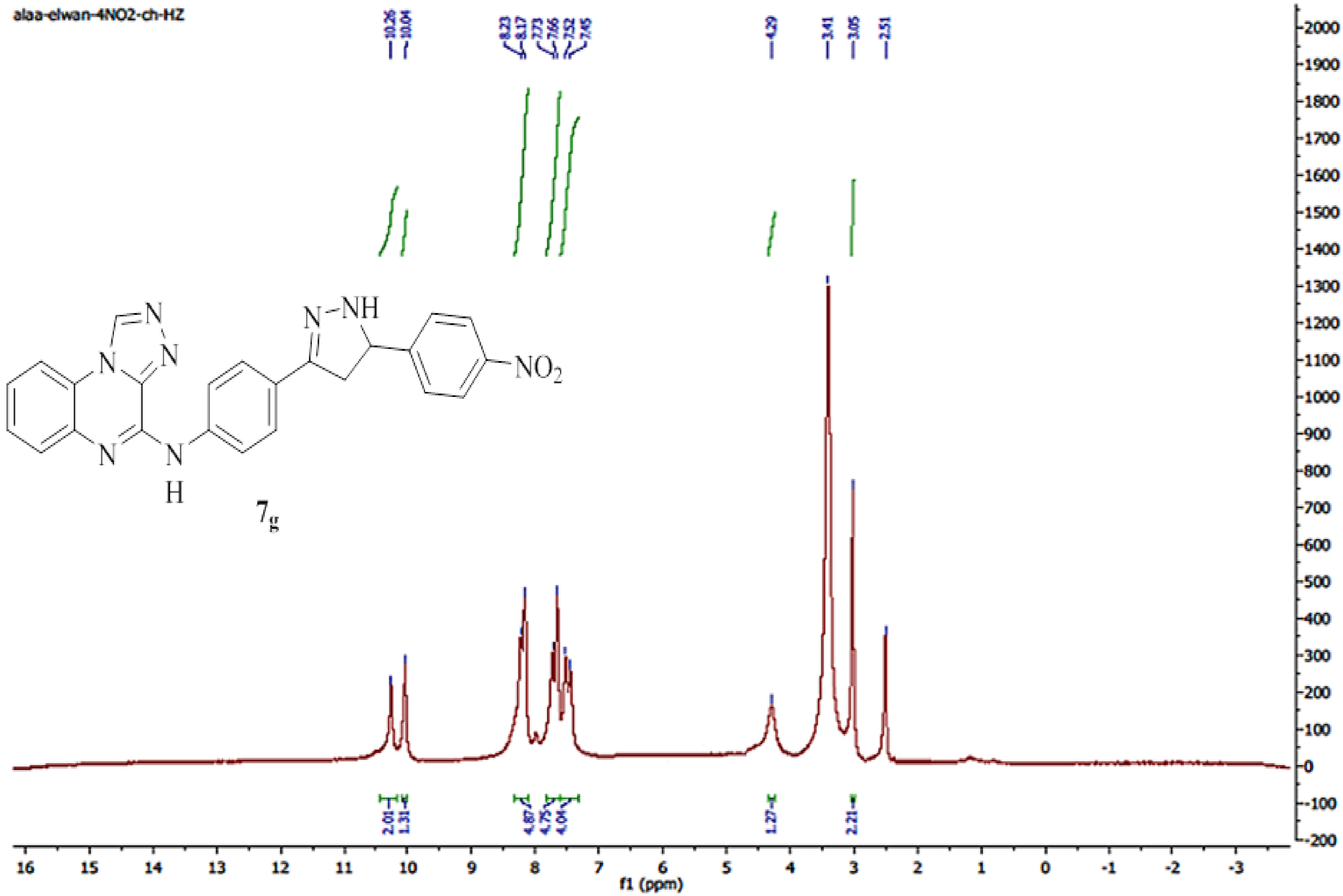

alaa-elwan-4NO2-ch-HZ
10.26
10.04
8.23
8.17
7.73
7.66
7.52
7.45
4.29
3.41
3.05
2.51
N
NH
N
N
N
N
N
H
NO_2
7_g
2.01
1.31
4.87
4.75
4.04
1.27
2.21
f1 (ppm)

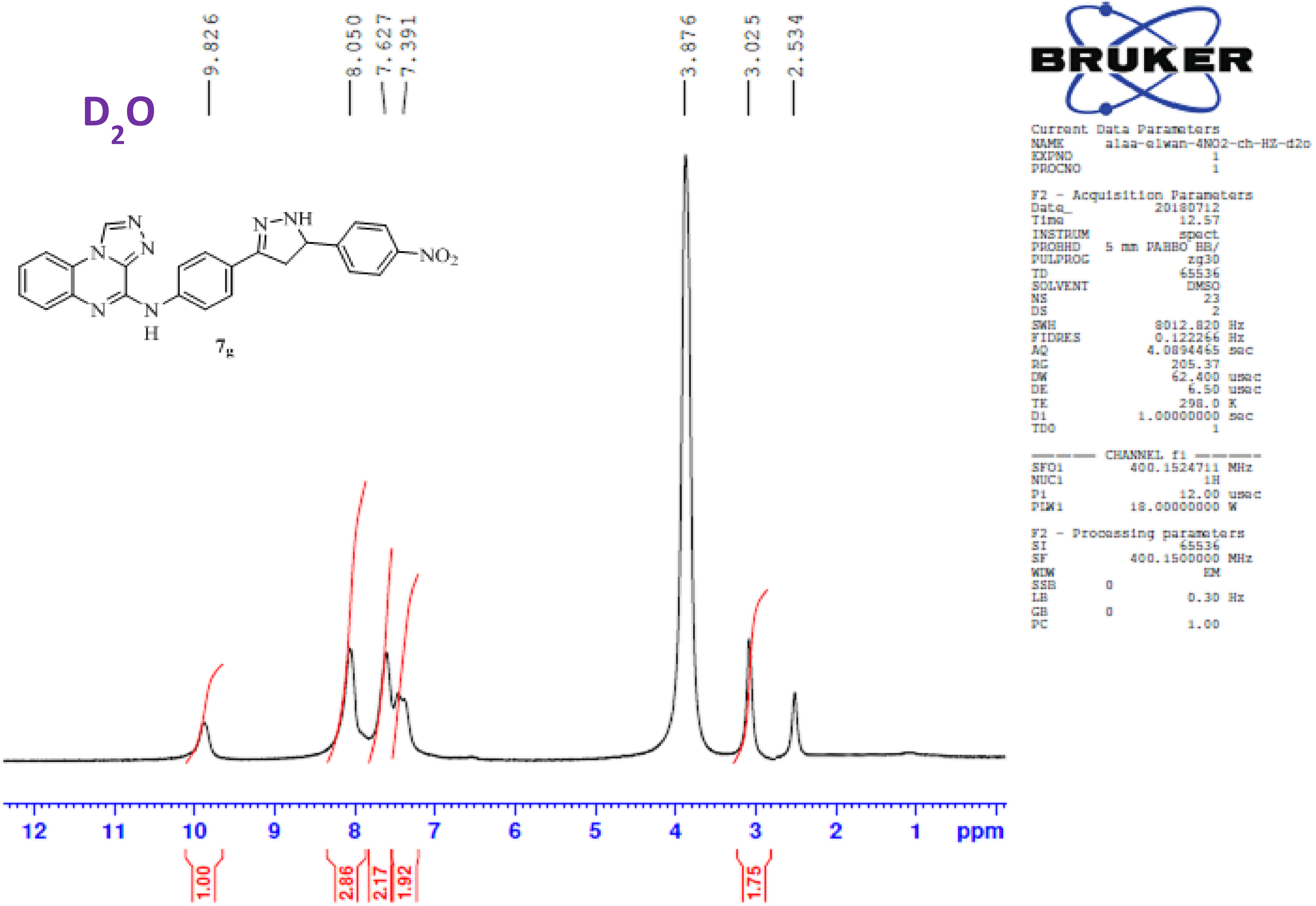

D2O
9.826
8.050
7.627
7.391
3.876
3.025
2.534
N
N
N
N
N
H
N
NH
NO2
7g
BRUKER
Current Data Parameters
NAME alaa-alwan-4NO2-ch-HZ-d2o
EXPNO 1
PROCNO 1
F2 - Acquisition Parameters
Date_ 20180712
Time 12.57
INSTRUM spect
PROBHD 5 mm PABBO BB/
PULPROG zg30
TD 65536
SOLVENT DMSO
NS 23
DS 2
SWH 8012.820 Hz
FIDRES 0.122266 Hz
AQ 4.0894465 sec
RG 205.37
DW 62.400 usec
DE 6.50 usec
TE 298.0 K
D1 1.00000000 sec
TD0 1
CHANNEL f1
SFO1 400.1524711 MHz
NUC1 1H
P1 12.00 usec
PLW1 18.00000000 W
F2 - Processing parameters
SI 65536
SF 400.1500000 MHz
WDW EM
SSB 0
LB 0.30 Hz
GB 0
PC 1.00
12 11 10 9 8 7 6 5 4 3 2 1 ppm
1.00
2.86
2.17
1.92
1.75
